# Identification of mono-ADP-ribose readers using well-defined photoaffinity-based probes

**DOI:** 10.1039/d5cb00176e

**Published:** 2025-11-28

**Authors:** Femke L. A. M. van der Heijden, Suzanne A. Weijers, Spyridoula Kondyli, Onno Bleijerveld, Michiel Vermeulen, Dmitri V. Filippov

**Affiliations:** a Leiden Institute of Chemistry, Leiden University, Einsteinweg 55 2333 CC Leiden The Netherlands filippov@chem.leidenuniv.nl; b Division of Molecular Genetics, The Netherlands Cancer Institute Plesmanlaan 121 1066 CX Amsterdam The Netherlands mi.vermeulen@nki.nl; c Department of Molecular Biology, Faculty of Science, Radboud Institute for Molecular Life Sciences, Oncode Institute, Radboud University Geert Grooteplein 28 6525 GA Nijmegen The Netherlands michiel.vermeulen@ru.nl

## Abstract

Adenosine diphosphate ribosylation is a significant post-translational modification implicated in various cellular processes and diseases, yet identifying its mono-ADP-ribose readers has posed considerable challenges. Previous proteomic screenings have predominantly focused on poly-ADP-ribose, resulting in the oversight of mono-ADP-ribose readers due to undefined ADP-ribose structures with randomly placed photo-crosslinking moieties. This study introduces novel, well-defined mono-ADP-ribose photoaffinity-based probes featuring distinct diazirine and benzophenone photo-crosslinkers aimed at selectively identifying mono-ADP-ribose readers. Using human HeLa protein extracts, these probes were employed in an interactomics screening, successfully uncovering numerous known and putative mono-ADP-ribose readers, including MACROD1. This study highlights the potential of these novel probes as powerful tools for exploring the mono-ADP-ribose interactome, thereby enhancing the understanding of ADP-ribosylation signaling within cellular contexts. Proteomics data are available *via* ProteomeXchange with identifier PXD065574.

## Abstract 2

This study introduces new photoaffinity-based probes to identify mono-ADP-ribose readers. Using human cell extracts, it detects both known and potential readers, thereby improving our understanding of mono-ADP-ribosylation.

## Graphical abstract

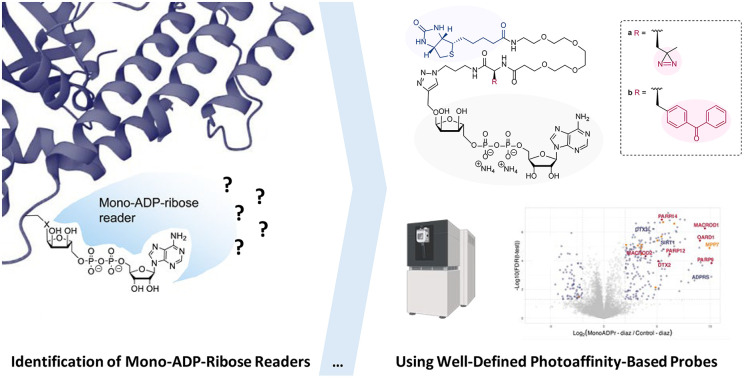

## Introduction

Adenosine diphosphate ribosylation (ADP-ribosylation) is an important post-translational modification that plays a crucial role in various cellular processes and diseases, including cancer and viral infections.^1–12^ This modification exists in several structural forms: mono-ADP-ribose (where one ADP-ribose unit is attached to the target), linear poly-ADP-ribose (where multiple ADP-ribose units are attached in a linear chain), and branched poly-ADP-ribose (where multiple ADP-ribose units form a branched chain).^13,14^ Proteins that interact with ADP-ribose are known as “readers,” and understanding the specific interactions between these readers and the various structural forms of ADP-ribose is essential for comprehending the mechanisms of ADP-ribosylation in both health and disease as well as for developing effective drugs that target the ADP-ribose signaling pathway.^15^

Although previous studies have attempted to identify readers of ADP-ribose using proteomic screenings, several limitations exist, especially in terms of identifying mono-ADP-ribose readers.^16–18^ For example, in the study conducted by Dasovich *et al.*,^16^ ADP-ribose readers were identified using biotinylated 8- and 40-mer poly-ADP-ribose photoaffinity-based probes. However, mono-ADP-ribose readers may have been missed due to the use of poly-ADP-ribose structures. Moreover, the ADP-ribose structures of these probes were not clearly defined as well; the enzymatically generated poly-ADP-ribose could consist of either mixed linear or branched chains, and the position of the benzophenone photo-crosslinker was random. As a result, the readers identified in this screening cannot be precisely linked to their corresponding (poly-)ADP-ribose structure. Furthermore, the results may have been affected by the random placement of the photo-crosslinker. In the study by Lam *et al.*,^17^ a bifunctional NAD^+^ compound with a diazirine photo-crosslinker and an azide click handle was used to generate poly-ADP-ribose enzymatically for proteomic screening of ADP-ribose readers. Similarly, due to the nature of the enzymatically generated poly-ADP-ribose, this approach shares the same limitations as the previously mentioned study by Dasovich *et al.*^16^ In the proteomic screening performed by Kliza *et al.*,^18^ ADP-ribose readers were identified using well-defined biotinylated mono-, di-, and linear tri-ADP-ribose affinity-based probes. This method successfully distinguished readers selectively among different ADP-ribose structures, including those interacting with mono-ADP-ribose. However, the absence of a photo-crosslinker in these probes limited the ability to differentiate between direct ADP-ribose readers and indirect protein–protein interactors.

**Fig. 1:**
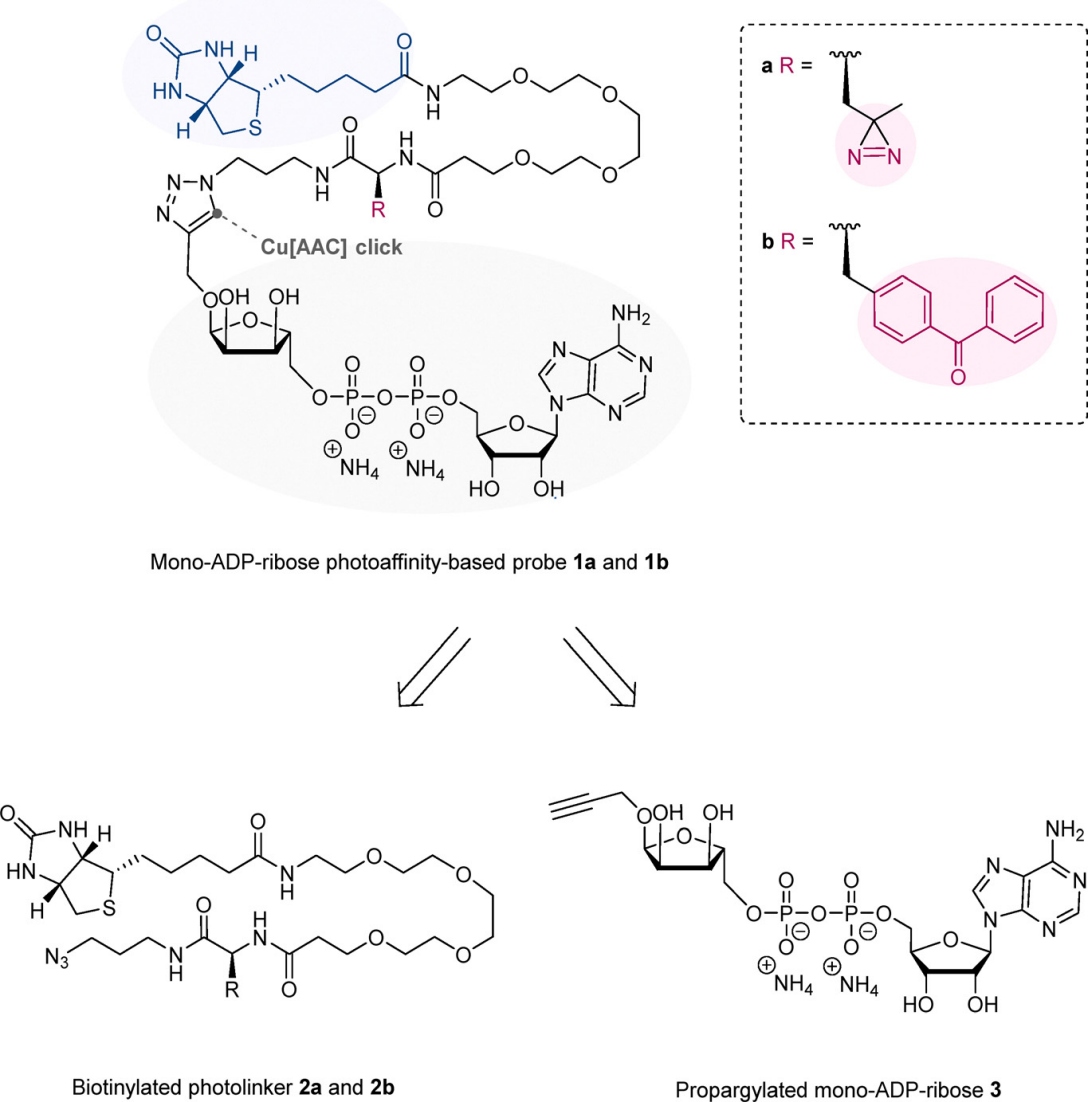
Molecular structures and retrosynthetic design of mono-ADP-ribose photoaffinity-based probes 1a and 1b. Mono-ADP-ribose structure is highlighted in grey, photo-crosslinkers are highlighted in pink and biotin handle is highlighted in blue. Mono-ADP-ribose probes 1a and 1b can be synthesized through a copper-catalyzed azide–alkyne cycloaddition (CuAAC) reaction, using propargylated mono-ADP-ribose 3.^18,19^ The synthesis further utilizes a versatile azide-containing biotinylated photolinker as one of the starting materials, which can include either a distinct diazirine (2a) or benzophenone (2b) for photo-crosslinking. Moreover, the biotinylated photo-crosslinkers 2a and 2b can serve as supplementary controls in the proteomic screening for mono-ADP-ribose readers, allowing for the filtration of proteins that preferentially bind to the linker component rather than the mono-ADP-ribose structure.

This work addresses the abovementioned limitations in identifying mono-ADP-ribose readers by developing novel, well-defined mono-ADP-ribose photoaffinity-based probes. Our approach features a biotinylated mono-ADP-ribose photoaffinity-based probe, which incorporates either a distinct diazirine (1a) or benzophenone (1b) photo-crosslinker to selectively identify mono-ADP-ribose readers (Fig. 1). The ability of these photoaffinity-based probes to covalently trap interacting proteins likely also facilitates the capture of transient or lower-affinity associations that would otherwise be lost during enrichment.^20^ Such interactions have been described for several ADPr-associated proteins that dynamically engage within biomolecular condensates formed by liquid–liquid phase separation, including DNA-damage foci and stress granules.^21^ These assemblies rely on weak, multivalent interactions that are typically disrupted under biochemical purification conditions, suggesting that photo-crosslinking may aid in capturing such transient associations. We applied these novel photoaffinity-based probes in an interactomics screening using human HeLa protein extracts and successfully identified numerous known and putative readers for mono-ADP-ribose, including proteins such as MACROD1 with an annotated ADP-ribose binding domain. Together, these results validate the photoaffinity mono-ADP-ribose probes as potent tools for proteome-wide capture of *bona fide* mono-ADP-ribose readers, thereby enabling our understanding of ADP-ribosylation signaling in greater detail.

## Results and discussion

### Synthesis of mono-ADP-ribose photoaffinity-based probes

Two novel photoaffinity-based probes were designed to selectively identify mono-ADP-ribose readers: probe 1a and probe 1b. Probe 1a contains a mono-ADP-ribose combined with a biotinylated linker and a diazirine as a photo-crosslinker, while probe 1b incorporates the same components but uses benzophenone as the photo-crosslinking group (Fig. 1). To synthesize the probes, a copper-catalyzed azide–alkyne cycloaddition (CuAAC) click reaction can be employed using propargylated mono-ADP-ribose 3^18,19^ and an azide-containing biotinylated photo-crosslinker; 2a for the incorporation of the diazirine moiety and 2b for the benzophenone. This approach not only allows for the straightforward generation of novel photoaffinity-based probes due to the use of the previously published propargylated mono-ADP-ribose structure^18,19^ but also offers several more advantages. First, the positioning of the photo-crosslinker within the biotinylated linker preserves the natural mono-ADP-ribose substrate, thus maximizing binding affinity with the readers. Second, biotinylated photo-crosslinkers 2a and 2b can serve as additional controls in proteomic screenings, allowing the exclusion of any proteins that preferentially bind to the linker rather than the mono-ADP-ribose structure.

**Scheme 1:**
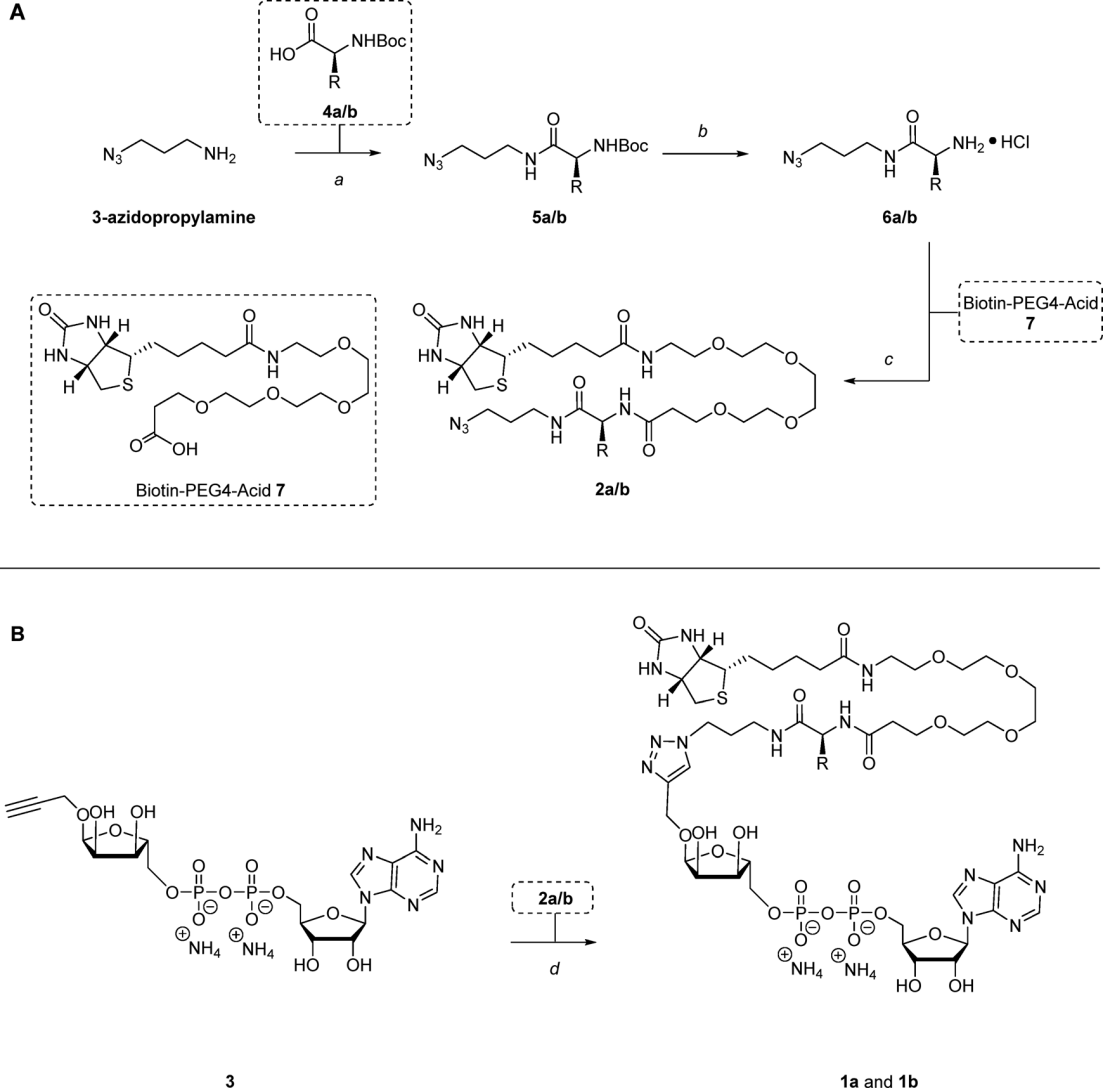
(A) Synthesis of biotinylated linkers 2a and 2b. Reagents and conditions: (a) carboxylic acid 4a/b, HOBt·H_2_O, EDC·HCl, DCM, rt, overnight, 59% for 5a, 74% for 5b. (b) HCl, dioxane, rt, 1–3 h. (c) Biotin-PEG4-acid 7, DiPEA, HOBt·H_2_O, EDC·HCl, DCM, rt, overnight, 55% for 2a, 79% for 2b. (B) Synthesis of mono-ADP-ribose photoaffinity-based probes 1a and 1b through an efficient CuAAC click reaction. Reagents and conditions: (d) 2a/b, CuSO_4_, sodium ascorbate, THPTA, H_2_O/ACN, rt, 1–19 h, 22% for 1a, 18% for 1b.

To synthesize photoaffinity-based probes 1a and 1b, the biotinylated linkers 2a and 2b were prepared first. This was achieved through an efficient three-step procedure, starting from commercially available 3-azidopropylamine (Scheme 1A). In the first step, an EDC-mediated amide coupling was carried out with HOBt to introduce the photo-crosslinker into the linker. This reaction used commercially available amino acids 4a and 4b as readily accessible and reactive carboxylic acid building blocks. Alternatively, compound 4a (Boc-l-photoleucine) may be synthesized following the procedures outlined by Yang *et al.*, starting from Boc-l-aspartic acid 1-methyl ester (Scheme S1).^22^ To include the photo-crosslinking diazirine moiety, Boc-l-photo-leucine 4a was applied in the amide coupling with 3-azidopropylamine using EDC·HCl and HOBt·H_2_O, yielding intermediate 5a with 59%. The benzophenone group was installed following the same coupling procedure using Boc-4-benzoyl-l-phenylalanine 4b as carboxylic acid, generating intermediate 5b with 74%. With the photo-crosslinker in place, the second step involved the deprotection of the Boc groups using HCl in dioxane, resulting in the formation of amines 6a and 6b, which were used in the next step without further purification. Lastly, the biotin tag and PEG4-linker were incorporated through another EDC-mediated amide coupling using HOBt·H_2_O and commercially available biotin-PEG4-acid 7, producing compounds 2a in 55% and 2b in 79% in the third step.

With compounds 2a and 2b prepared, probes 1a and 1b could be synthesized next (Scheme 1B). To this end, 1-*O*-propargyl-α-mono-ADP-ribose 3 was synthesized according to the methods outlined by Liu *et al.*^18,19^ The generation of mono-ADP-ribose probes 1a and 1b was achieved through a CuAAC click reaction, using 1-*O*-propargyl-α-mono-ADP-ribose 3 along with the corresponding biotinylated linkers 2a and 2b in the presence of CuSO_4_, sodium ascorbate, and THPTA in a mixture of H_2_O and ACN, yielding probe 1a in 22% and 1b in 18%.

### Identification of mono-ADP-ribose-binding proteins

**Fig. 2:**
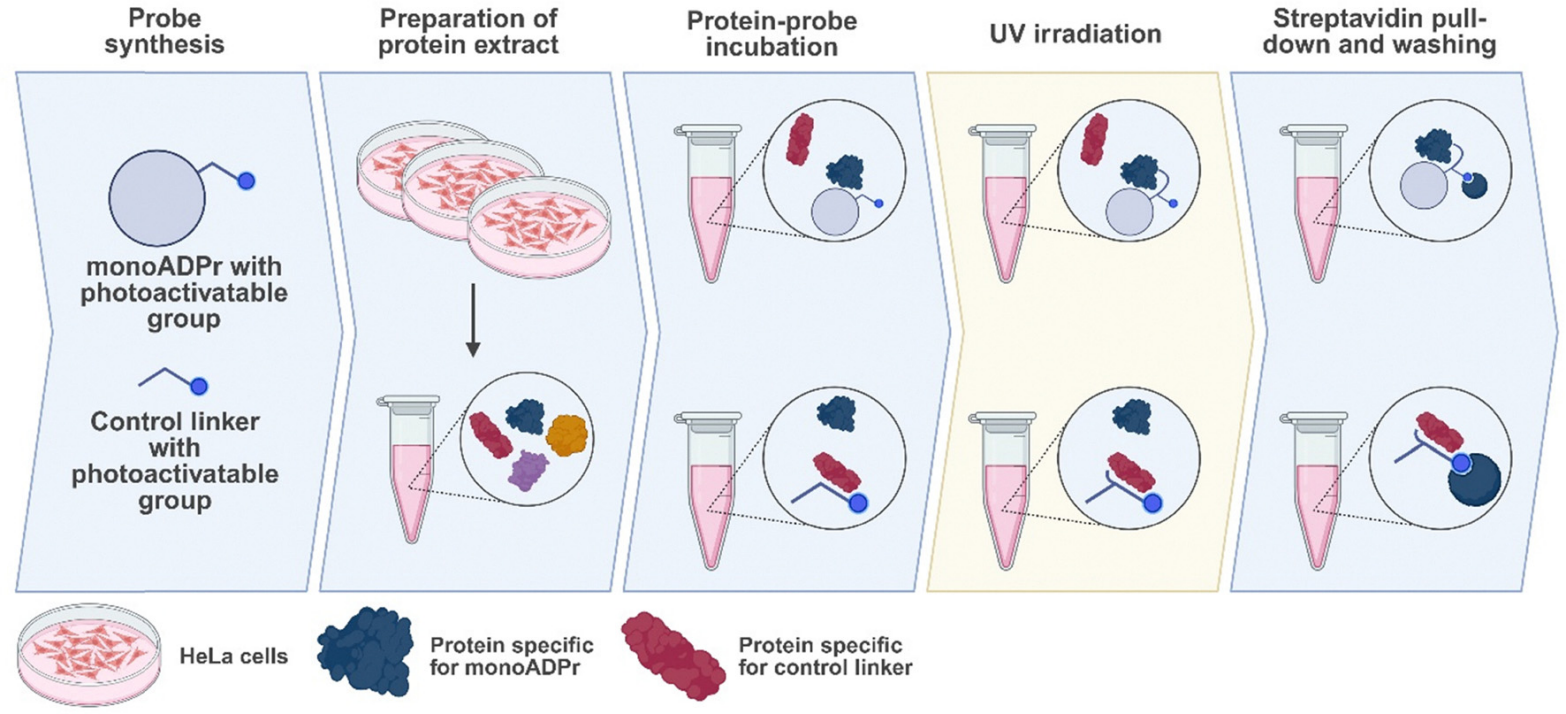
Workflow to identify candidate direct interactors of mono-ADP-ribose. Mono-ADP-ribose (compounds 1a and 1b) and the control linker (compounds 2a and 2b) are each incubated in protein extract prepared from HeLa cells. After UV irradiation where interacting proteins become covalently bound to each probe, the probe-protein complexes are pulled down by streptavidin beads and background proteins (not covalently bound to the probe) are washed away. Enriched proteins are subjected to on-bead digestion and then measured with a mass spectrometer. Image created using Biorender.com.

Following the synthesis of these compounds, the compounds were incubated in protein extract and subjected to UV irradiation. Covalently bound proteins were enriched *via* a streptavidin pull-down, washed under denaturing conditions to remove any background proteins, and ultimately detected using mass spectrometry (Fig. 2). To identify proteins specifically interacting with the mono-ADP-ribose moiety, compounds 2a and 2b are used as controls to enrich for proteins that interact only with the diazirine, benzophenone, or biotin-linker.

**Fig. 3:**
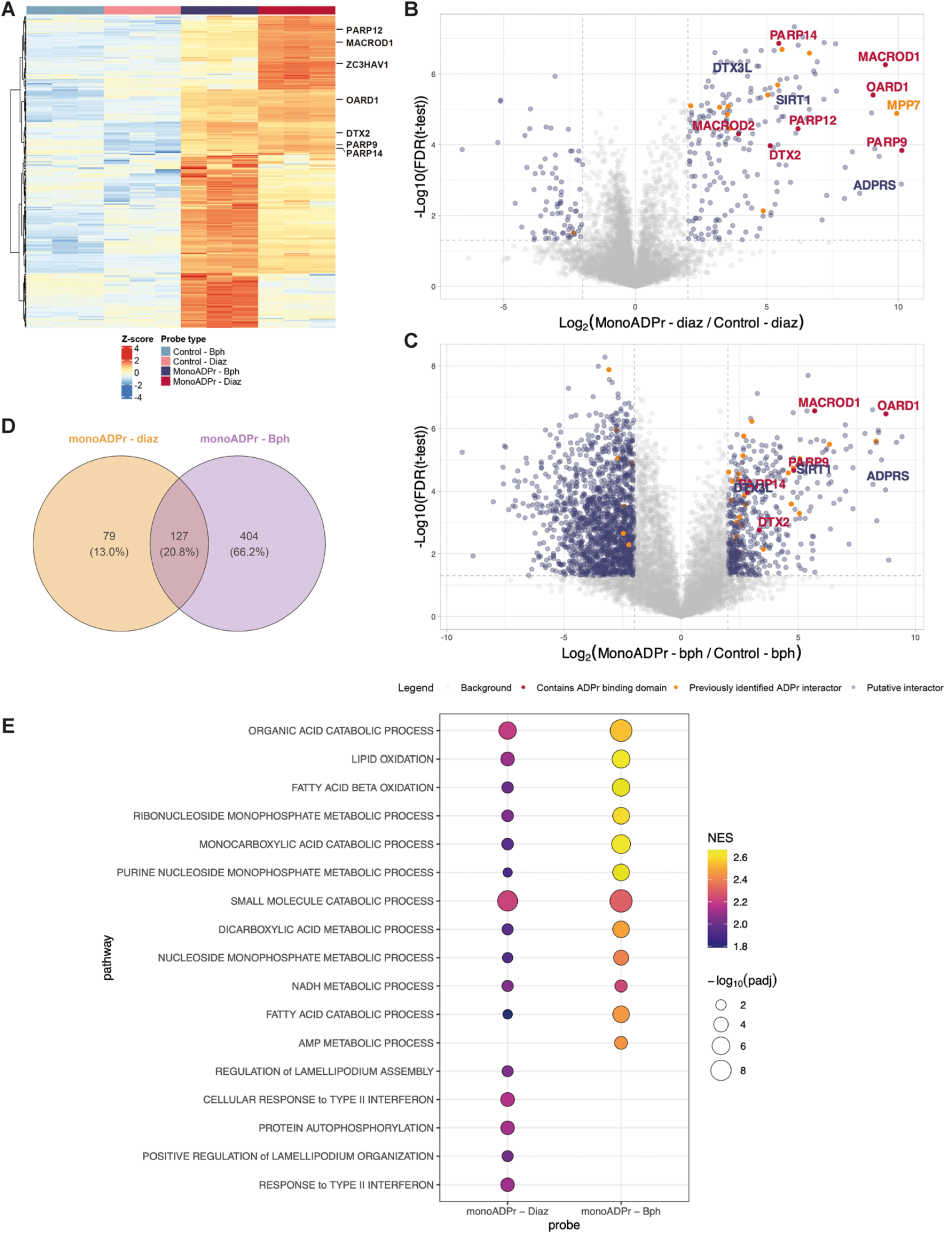
Affinity pull-down with compounds 2b (control – Bph), 2a (control – Diaz), 1b (mono-ADP-ribose – Bph), and 1a (mono-ADP-ribose – Diaz). (A) Hierarchical clustering of significantly enriched proteins (ANOVA, Benjamini–Hochberg correction, FDR 0.05). Proteins displayed with an annotated ADP-ribose binding domain. (B) and (C) Volcano plots depicting preferential binding of proteins to compound 1a*versus* compound 2a, or compound 1b*versus* compound 2b, respectively. The statistical cutoffs in the *t*-test are as follows: FDR < 0.05 and FC ≥ 2. Proteins containing an ADP-ribose binding domain are indicated in red, proteins that have been identified by at least 2 interactomics methods are indicated in orange.^16–18,23^ (B) and (C) share the same legend. (D) Venn diagram depicting the significantly enriched proteins to either compound 1a or 1b when compared to their respective controls (determined in the differential analyses: FDR < 0.05 and log 2 fold change (FC) > 2). (E) Gene set enrichment analysis (GSEA) for proteins significantly enriched by the mono-ADP-ribose photoaffinity probes. Proteins enriched for compound 1a and 1b against their respective control proteins were ranked by log 2 fold change and tested against Gene Ontology Biological Process Terms. The union of the top positively enriched pathways from each probe is displayed; pathways with an adjusted *p*-value >0.05 were excluded. Bubble size indicates −log_10_ (padj), and color represents the normalized enrichment score (NES).

Hierarchical clustering of significantly enriched proteins revealed distinct probe-dependent interaction profiles, with clear separation between the mono-ADP-ribose probes and their respective controls (Fig. 3A). Several proteins identified in the screen contain annotated ADP-ribose binding domains, including macrodomains (MACROD1, OARD1, PARP9/14) and WWE domains (DTX2, PARP12/14, ZC3HAV1). These proteins exhibited minimal enrichment to the control compounds (both benzophenone- and diazirine-based), but were consistently enriched by both mono-ADP-ribose probes. Notably, MACROD1, PARP12, and ZC3H1AV1 grouped together in the hierarchical clustering and showed preferential enrichment with the mono-ADP-ribose – diazirine probe, suggesting a shared binding behavior under these conditions. In contrast, other interactors such as DTX2, OARD1, and PARP9/14 displayed a more balanced binding to both crosslinkers, potentially indicating subtle crosslinker-dependent binding preferences.

To further dissect the interaction profiles of both probes, pairwise differential analyses were performed to compare each mono-ADP-ribose probe to its corresponding control (Fig. 3B and C). The diazirine-based probe yielded a more sharply defined set of significantly enriched proteins compared to its benzophenone analogue (Fig. 3D). This difference likely reflects the distinct photochemical properties of both crosslinkers: benzophenones form relatively long-lived diradical intermediates that increase the likelihood of non-specific labeling, whereas diazirines generate short-lived carbene intermediates that can be quenched rapidly by solvent molecules, reducing background at the cost of somewhat lower labeling efficiency.^22,24^ Despite these differences in labeling behavior, both probes consistently captured known monoADPr interactors, confirming that their enrichment profiles primarily reflect ADPr-specific binding rather than crosslinker artifacts. Among these, DTX3L and PARP9, previously reported as a mono-ADP-ribose recognition pair, were co-enriched.^25^ The ADP-ribose “eraser” ARH3 (ADP-ribosylhydrolase 3) was likewise enriched by both probes, reinforcing their capacity to enrich for functionally relevant ADP-ribose interactors. To contextualize these findings to previous ADP-ribose interactomics approaches, proteins also identified in least two other methods are highlighted in the plots, as recurrent identification across independent mass spectrometry datasets increases confidence that these represent *bona fide* ADPr interactors rather than dataset- or method-specific findings. Interestingly, proteins previously associated with poly-ADP-ribose were identified, such as MPP7, suggesting that mono-ADPr-binding and poly-ADPr-binding proteins can partly overlap, potentially reflecting shared structural features of ADPr recognition.

We additionally examined whether the differences in significantly enriched proteins between compounds 1a and 1b would also translate into distinct biological functions. Therefore, gene set enrichment analysis was performed (Fig. 3E). Despite the larger number of interactors enriched to the benzophenone probe, enrichment profiles for both probes converged on nucleotide-metabolic pathways, including purine, nucleobase and nucleoside-phosphate metabolism, processes that have previously been connected to PARP activity and ADP-ribose signaling.^26^ Concordant enrichment of lipid-oxidation signatures was also observed, consistent with reports that several PARP family members modulate fatty-acid beta-oxidation and broader lipid homeostasis.^27^ These findings suggest that while the diazirine-based probe yields a more selective set of enriched proteins and the benzophenone-based probe captures a broader spectrum, the biological pathways represented by each interactor are largely conserved. This highlights the robustness of the mono-ADP-ribose-protein interaction network and further supports the conclusion that both compounds effectively enrich for core ADP-ribose-associated proteins.

To further assess the reproducibility of these results, an independent biological replicate was performed under a modified setup, in which the pull-down was carried out in a filter plate format to reduce reaction volumes, including the amounts of protein extract and probe. Despite the reduced input in the replicate experiment, enrichment of canonical ADPr readers such as MACROD1 and OARD1 was confirmed, with the diazirine-based probe showing markedly higher overlap between replicates (29.9%) compared to the benzophenone probe (8.2%) (Fig. S1A–D). Comparison of Log_2_ fold changes between biological replicates further highlighted this difference, showing strong correlation for the diazirine-based probe, whereas replicate agreement for the benzophenone-based probe was minimal (Fig. S1E). Together with the sharper enrichment profile observed for the diazirine in biological replicate 1 (Fig. 3C and D), these data indicate that the diazirine-based probe yields more consistent enrichment under the tested conditions.

Because probe architecture was still considered a potential source of bias, and additional comparison with a previously synthesized mono-ADP-ribose probe was performed, which features a shorter linker and lacks a diazirine group (Fig. S2A).^18^ Compound 1a was selected given the more selective enrichment profile and smaller photoactivatable group compared to compound 1b. The experimental setup for this comparison reflected the original pull-down conditions suitable for that probe, therefore no UV irradiation was performed and the samples were only mildly washed. Compound 1a yielded a substantial overlap in interactome with the non-photoactivatable mono-ADP-ribose probe, confirming that it retains core specificity for canonical ADP-ribose readers such as MACROD1 and PARP9/DTX3L (Fig. S2B–D). In addition, compound 1a enriched a greater number of interactors and exhibited higher Log_2_ fold enrichment values, including proteins previously linked to poly-ADP-ribose binding. These findings suggest that the slightly extended linker tethering the diazirine improves probe-protein docking and, upon photoactivation, enables covalent stabilization of lower-affinity interactions. The ability to stabilize such weak, short-lived associations is particularly relevant for ADPr-associated proteins that participate in dynamic, condensate-like assemblies within the cell.^21^

**Fig. 4:**
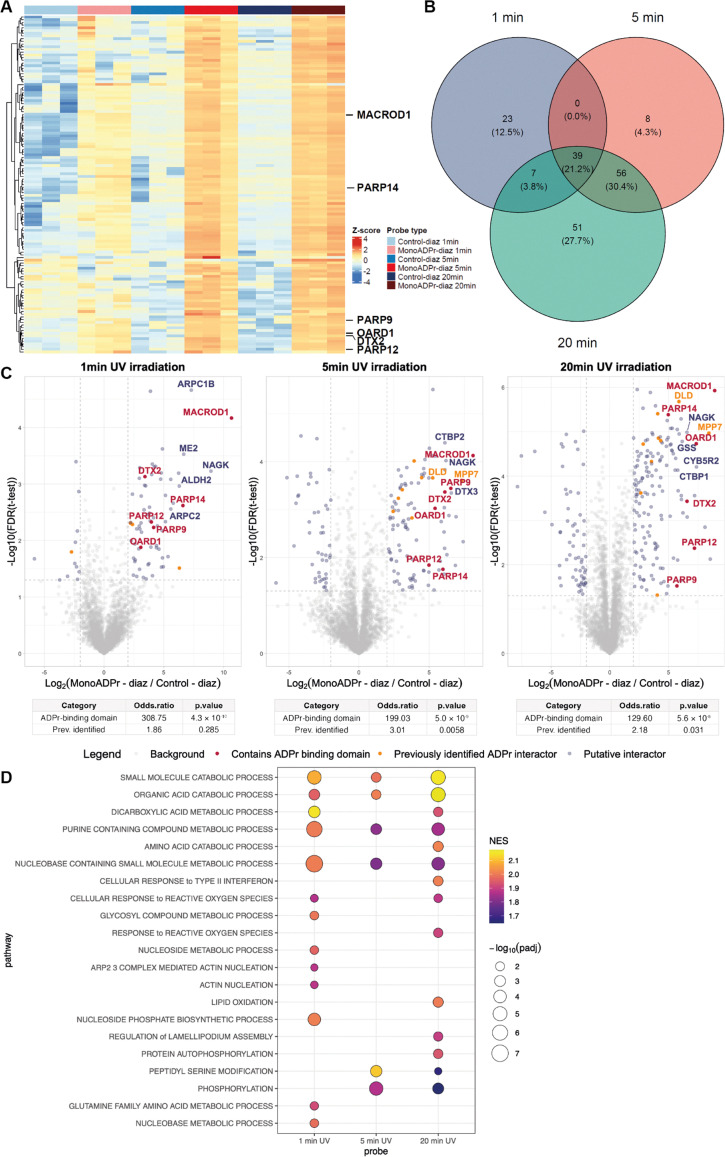
Optimization of UV irradiation times for compound 1a when compared against compound 2a. (A) Hierarchical clustering of significantly enriched hits (ANOVA, Benjamini-Hochberg correction, FDR 0.05). The proteins displayed next to the heatmap contain an annotated ADP-ribose binding domain. (B) Venn diagram depicting the overlap in significantly enriched proteins to compound 1a when compared against compound 2a (determined in the differential analyses: FDR < 0.05 and log 2 fold change (FC) > 2). (C) Volcano plots depicting proteins preferentially enriched by compound 1a over 2a following 1, 5, or 20 min of UV irradiation. Proteins containing an ADP-ribose binding domain are indicated in red, proteins that have been identified by at least 2 interactomics methods are indicated in orange.^16–18,23^ All volcano plots share the same legend. Below each plot, Fisher's exact test results summarize the enrichment of proteins with annotated ADP-ribose binding domains or previously reported ADPr interactors among the significantly enriched proteins (FDR < 0.05 and FC > 2). (D) Gene set enrichment analysis of enriched proteins by compound 1a compared to its control across different UV irradiation times. Shown are the top GO:BP pathways with positive normalized enrichment scores (NES), selected from the union of pathways that are significant (*p*_adj_ < 0.05) in at least one timepoint. Bubble color represents the normalized enrichment score (NES), and size corresponds to −log_10_(*p*_adj_).

Because the efficiency and specificity of photo-crosslinking depend on irradiation parameters, we next evaluated how UV exposure time influenced probe performance.^28^ The carbene intermediate generated upon diazirine photoactivation has a lifetime on the nanosecond scale in polar environments, suggesting that shorter irradiation times could already suffice for efficient crosslinking. We therefore varied UV exposure (1, 5, or 20 min) while comparing the enrichment of proteins to compound 1a*versus*2a. Hierarchical clustering of significantly enriched proteins revealed that UV exposure time strongly affected labeling efficiency (Fig. 4A). At 1 minute of irradiation, only modest separation between probe and control was observed. In contrast, 5 minutes of exposure yielded a robust enrichment profile, especially for canonical ADP-ribose readers such as MACROD1, PARP14, PARP9, OARD1, DTX2 and PARP12. Extending irradiation to 20 minutes resulted in an enrichment pattern that closely resembled the 5-minute profile, without further improvement for canonical interactors.

To assess the overlap between enrichment profiles, differential analyses for each condition were compared (Fig. 4B and C). 39 proteins (21.1%) were shared across all three irradiation times, which included all canonical ADPr readers identified in this experiment. An additional 51 proteins were uniquely enriched after 20 minutes, suggesting that prolonged irradiation increases total yield but also the likelihood of non-specific labeling. In such cases, competition labeling where excess unmodified monoADPr is included during the pull-down can help distinguish specific ADPr-dependent interactions from background labeling. Although not performed in the present study, this approach could further validate the specificity of identified interactors in future applications. To quantitatively assess the selectivity achieved under each condition, Fisher's exact tests were performed, revealing a strong and significant enrichment of proteins containing annotated ADPr-binding domains across all timepoints (odds ratios ranging from 129–308, *p* < 10^−7^–10^−10^), whereas enrichment of previously reported ADPr interactors was modest and only significant at 5- and 20-minutes UV irradiation (odd ratios ranging from 2–3, *p* ≤ 0.03). Together, these results indicate that 5 minutes of UV irradiation provides an optimal balance between specificity and coverage.

Finally, to examine whether UV exposure time influenced the biological processes recovered, GSEA was performed across all three datasets (Fig. 4D). At 1 minute, enrichment was dominated by diverse metabolic pathways, including nucleobase- and organic-acid-containing small-molecule metabolism, consistent with rapid labeling of abundant enzymes. After 5 minutes, the significant pathways shifted toward signaling-related processes such as protein autophosphorylation, peptidyl-serine modification, and phosphorylation, while metabolic terms diminished. Prolonged irradiation (20 minutes) largely reinstated the metabolic signature observed at 1 minute, together with a modest enrichment of phosphorylation-related categories. These results indicate that short and prolonged exposures primarily capture metabolic enzymes, some of which may engage ADPr through NAD-binding folds or positively charged surface regions that enable electrostatic interactions with the ADPr phosphate backbone.^15^ In contrast, a 5-minute exposure provides an optimal balance between specificity and coverage by preferentially labeling *bona fide* ADPr-associated signaling proteins.

To define a high-confidence set of putative mono-ADPr interactors, enrichment profiles from non-crosslinked and photo-crosslinked pull-downs (Fig. 3, 4 and Fig. S1, S2) were combined, selecting proteins reproducibly enriched across both datasets (Table S1). The dataset comprises both canonical ADPr binding proteins and proteins without annotated ADPr domains. Among the former are MACROD1, OARD1, and PARP9/12/14. Several metabolic enzymes, including DLD, LDHA, and OGDH, together with NAD^+^-dependent regulators SIRT1 and SIRT4, were reproducibly enriched. These associations are consistent with previous reports linking ADP-ribosylation to metabolic and stress-responsive pathways, and may reflect indirect recognition or regulation by ADPr signaling, consistent with prior reports linking ARTD activity to metabolic processes.^13,29,30^ The transcriptional corepressors CTBP1 and CTBP2 were also identified, in line with their reported functional interplay with PARP1 in transcriptional regulation and DNA damage responses.^31,32^ Collectively, the data reveal a monoADPr-associated protein network encompassing both known readers and previously characterized candidates. While the precise molecular basis of these interactors remains to be defined, these proteins represent strong candidates for subsequent biochemical validation.

**Fig. 5:**
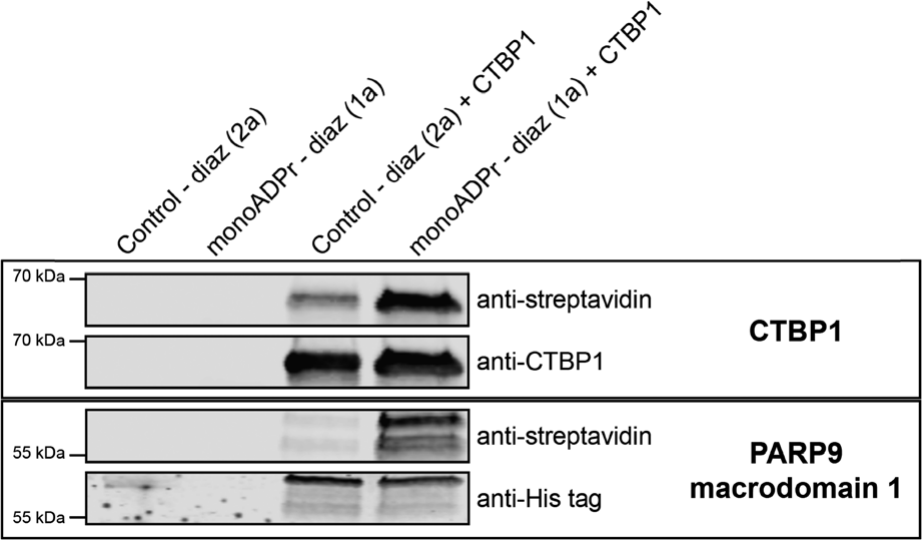
Validation of direct mono-ADP-ribose interaction by CTPB1. Recombinant CTBP1 and PARP9 macrodomain 1 (positive control) were incubated with either the control probe (2a) or the mono-ADPr-diazirine probe (1a). After UV irradiation, samples were separated by SDS-PAGE and subjected to immunoblotting. Probe-protein conjugates were detected with anti-streptavidin, and protein loading was verified using anti-CTBP1 or anti-His-tag antibodies. Lanes containing probe-only served as negative controls.

To experimentally validate direct mono-ADP-ribose interactions for selected candidates, we next carried out photo-crosslinking experiments using purified recombinant proteins followed by immunoblot detection. CTBP1 was selected as a representative “group 2” protein since it lacks an annotated ADPr-binding domain yet was reproducibly enriched by the mono-ADPr probe. As a reference, the macrodomain 1 of PARP9 was included, representing a canonical ADPr-binding protein (group 1) previously shown to associate with ADP-ribose in the PARP9-DTX3L complex.^33^ Following UV activation with the mono-ADPr photoaffinity probe (compound 1a) or the corresponding control probe (2a), probe-protein conjugates were detected by streptavidin immunoblotting, while anti-CTBP1 and anti-His-tag blots verified equal protein loading (Fig. 5 and Fig. S3). Both for CTBP1 and PARP9 macrodomain 1 a markedly stronger streptavidin signal was found when incubated with the monoADPr probe compared to the control probe, indicating preferential crosslinking mediated by the ADPr moiety. Together, these results demonstrate that both proteins can directly interact with compound 1a, validating CTBP1 as a *bona fide* mono-ADPr-binding protein consistent with its classification of Table S1. Although the observed crosslinking confirms a direct physical interaction between CTBP1 and mono-ADPr, the current experiments do not address whether this binding contributes to CTBP1's regulatory function. Future studies will be required to determine the physiological relevance of this interaction.

The ability of CTBP1 to directly bind mono-ADP-ribose suggests that ADP-ribosylation could influence its oligomeric state and transcriptional activity. CTBP1 functions as an NADH-dependent dimer or tetramer, and disruption of this multimeric assembly is known to counteract its repressive function.^34^ Non-covalent binding of ADPr may compete with NAD(H) at the Rossmann-like fold or alter its conformation, thereby weakening its assembly. Alternatively, mono-ADP-ribosylation of CTBP1 itself, as reported previously, may directly interfere with NADH binding or dimer stabilization.^35^ Both mechanisms would be expected to transiently destabilize CTBP1 multimerization and promote its dissociation from chromatin, providing a possible means by which ADP-ribosylation modulates CTBP1-mediate gene regulation.

## Conclusions

In conclusion, this work outlines the efficient synthesis of new mono-ADP-ribose photoaffinity-based probes 1a and 1b, highlighting their application in a proteomic screening to selectively identify the readers of mono-ADP-ribose. The probes were synthesized using an efficient CuAAC click reaction, which combined propargylated mono-ADP-ribose structure 3 with novel biotinylated photo-crosslinkers 2a and 2b. This straightforward synthetic strategy facilitated the rapid creation of new probes. Biotinylated linkers 2a and 2b are critical control compounds to filter out proteins that interact with the linker component rather than the mono-ADP-ribose structure itself. The novel photoaffinity-based probes were applied in an interactomics screening of human HeLa protein lysates, resulting in the identification of numerous known and novel candidate readers for mono-ADP-ribose. The diazirine-based probe (compound 1a) exhibited a more selective and reproducible enrichment profile than its benzophenone counterpart (compound 1b), while both captured functionally relevant ADP-ribose interactors and converged on similar biological processes in gene set enrichment analysis. Integration of comparative experiments refined this dataset into a high-confidence mono-ADP-ribose interactome and confirmed direct binding of selected candidate proteins to the mono-ADPr-based photoaffinity probe. Future work should focus on integrating this resource with functional and structural data to elucidate how the identified readers engage mono-ADP-ribose within specific biological contexts. Together, these findings and the developed probes establish a robust and reproducible framework for mapping the mono-ADP-ribose interactome and advancing the mechanistic understanding of ADP-ribosylation signaling.

## Author contributions

F. L. A. M. v. d. H.: investigation, methodology, conceptualisation, writing – original draft; S. A. W.: investigation, methodology, conceptualisation, writing – original draft; S. K.: investigation, resources; O. B.: investigation, resources; M. V.: conceptualisation, supervision, funding acquisition, writing – original draft, writing – review & editing. D. V. F.: conceptualisation, supervision, funding acquisition, writing – original draft, writing – review & editing.

## Conflicts of interest

There are no conflicts to declare.

## Supplementary Material

CB-007-D5CB00176E-s001

CB-007-D5CB00176E-s002

CB-007-D5CB00176E-s003

CB-007-D5CB00176E-s004

CB-007-D5CB00176E-s005

## Data Availability

The data supporting this article have been included as part of the supplementary information (SI). Supplementary information: detailed experimental procedures, NMR spectra of the probes and synthetic intermediates, supplementary figures. See DOI: https://doi.org/10.1039/d5cb00176e.
