# Supplementary figures and images for: Identification of mono-ADP-ribose readers using well-defined photoaffinity-based probes
Source: RSC Chem Biol. 2025 Nov 28;7(2):250–9. doi: 10.1039/d5cb00176e (PMC12679546; doi:10.1039/d5cb00176e)

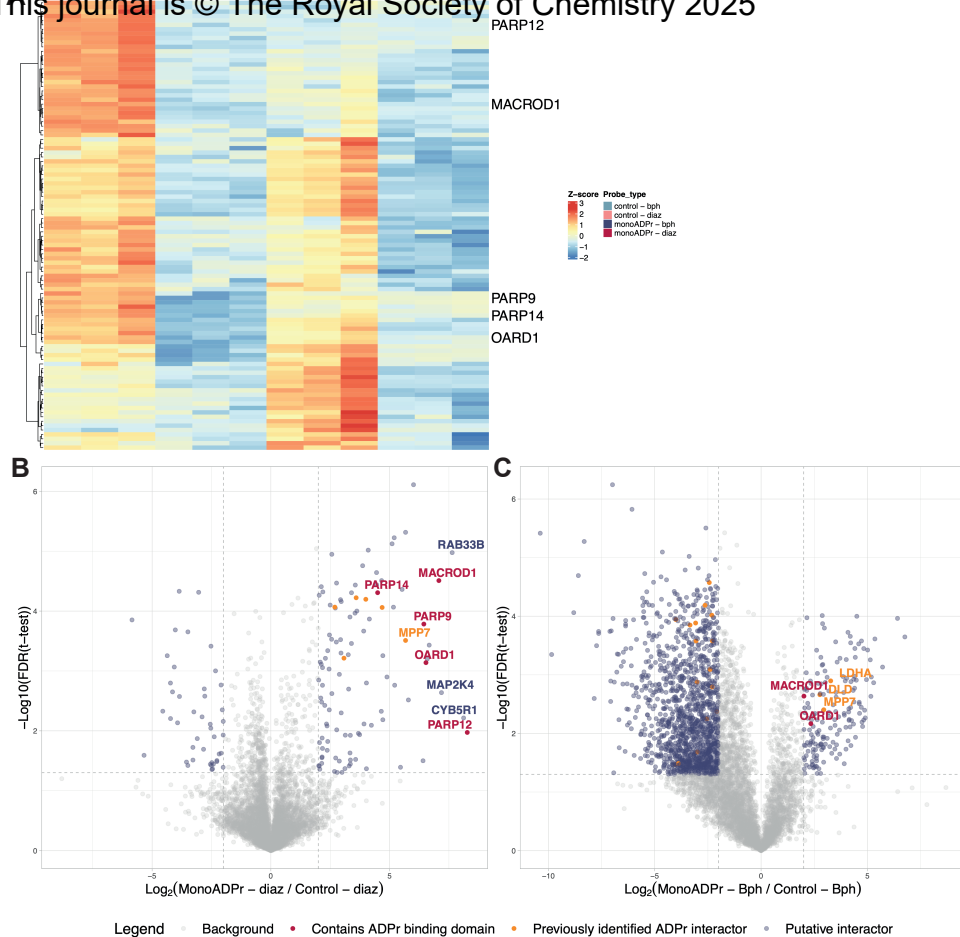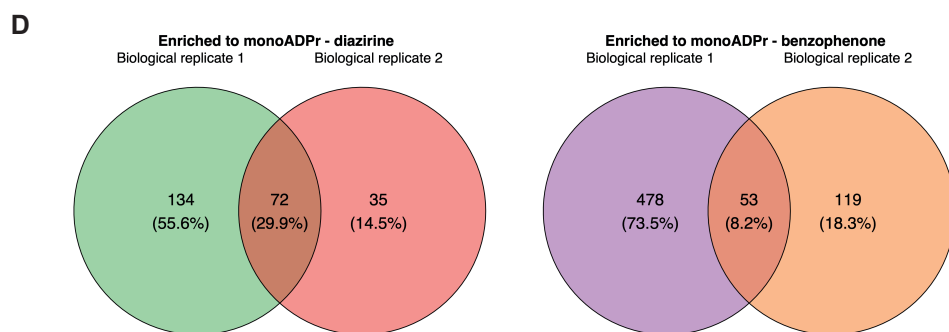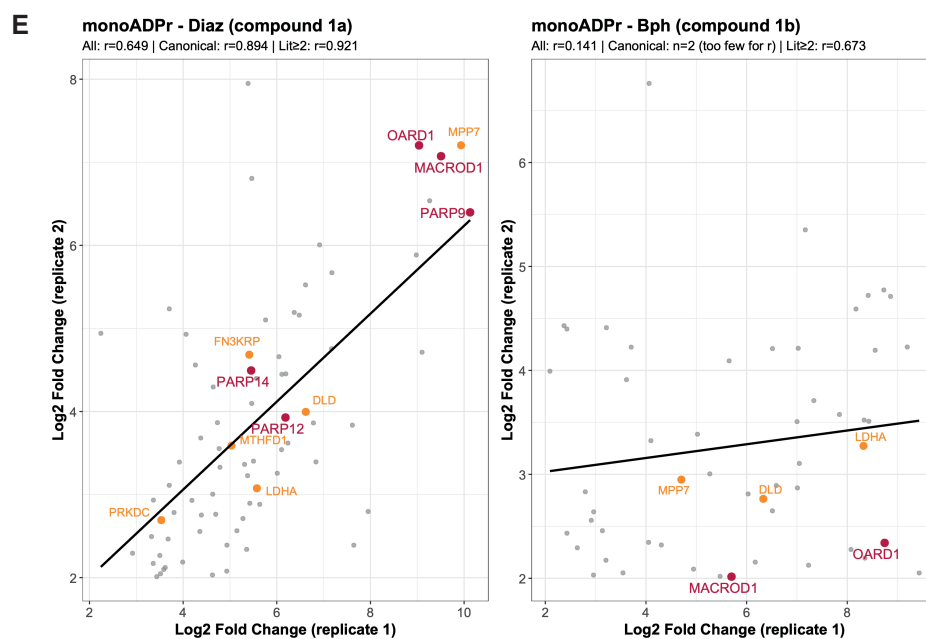

Supplement: CB-007-D5CB00176E-s002 [file CB-007-D5CB00176E-s002.pdf]
