# Supplementary material for: Identification of mono-ADP-ribose readers using well-defined photoaffinity-based probes
Source: RSC Chem Biol. 2025 Nov 28;7(2):250–9. doi: 10.1039/d5cb00176e (PMC12679546; doi:10.1039/d5cb00176e)
Supplement: CB-007-D5CB00176E-s005 [file CB-007-D5CB00176E-s005.pdf]

Supplementary Information (SI) for RSC Chemical Biology.
This journal is © The Royal Society of Chemistry 2025

Identification of Mono-ADP-Ribose Readers Using Well-Defined Photoaffinity-based Probes

Femke L. A. M. van der Heijden,[+a] *Suzanne A. Weijers,*[+b,c] *Spyridoula Kondyli,*[b] *Onno Bleijerveld,*[b] *Michiel Vermeulen,*[*b,c] *Dmitri V. Filippov*[*a]

[a] Leiden Institute of Chemistry, Leiden University, Einsteinweg 55, 2333 CC, Leiden (The Netherlands), E-mail: filippov@chem.leidenuniv.nl

[b] Division of Molecular Genetics, The Netherlands Cancer Institute, Plesmanlaan 121, 1066 CX, Amsterdam (The Netherlands), E-mail: mi.vermeulen@nki.nl

[c] Department of Molecular Biology, Faculty of Science, Radboud Institute for Molecular Life Sciences, Oncode Institute, Radboud University, Geert Grooteplein 28, 6525 GA, Nijmegen (The Netherlands), E-mail: michiel.vermeulen@ru.nl

Table of Contents

1. Additional Figures S2
2. Experimental Section S6
3. Characterization Data S17
4. Additional References S34

1. Additional Figures

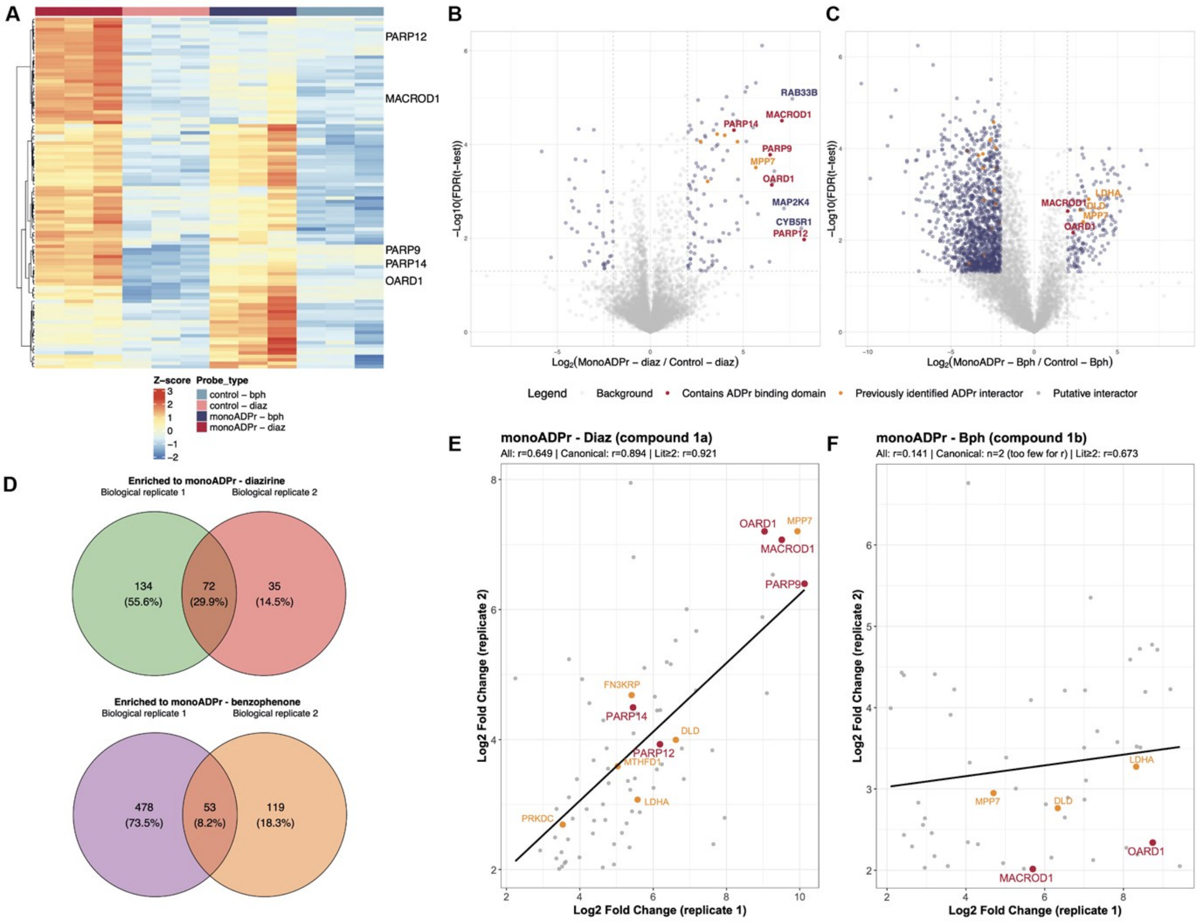

Figure S1. Reproducibility of interactomes with compounds **1a** and **1b** in an independent biological replicate. This replicate was performed using an alternative experimental setup (filterplate with reduced protein extract; see “2. Experimental Section: UV irradiation and subsequent streptavidin pull-down” for more details). (A) Hierarchical clustering of significantly enriched proteins (ANOVA, Benjamini-Hochberg correction, FDR 0.05). The displayed protein names contain an annotated ADP-ribose binding domain. (B-C) Volcano plots depicting preferential binding of proteins to compound **1a** versus compound **2a**, or compound **1b** versus compound **2b**, respectively. The statistical cut-offs are in the t-test as follows: FDR < 0.05 and FC ≥ 2. Proteins containing an ADP-ribose binding domain are indicated in red, proteins that have been identified by at least 2 interactomes are indicated in orange.[1–4] (B) and (C) share the same legend. (D) Venn diagrams showing the overlap in interactomes between the biological replicates for compound **1a** and compound **1b**, respectively. (E-F) Correlation plot comparing the Log_2 fold changes of significantly enriched proteins (FC > 2) between biological replicates for compound **1a** and compound **1b**, respectively. Proteins containing a canonical ADP-ribose binding domain are labelled in red (‘Canonical’) and proteins labelled orange are proteins that have been identified by at least 2 interactomics methods before (‘Lit’). Pearson correlation coefficients (r) are indicated for these, including for all enriched proteins (‘All’).

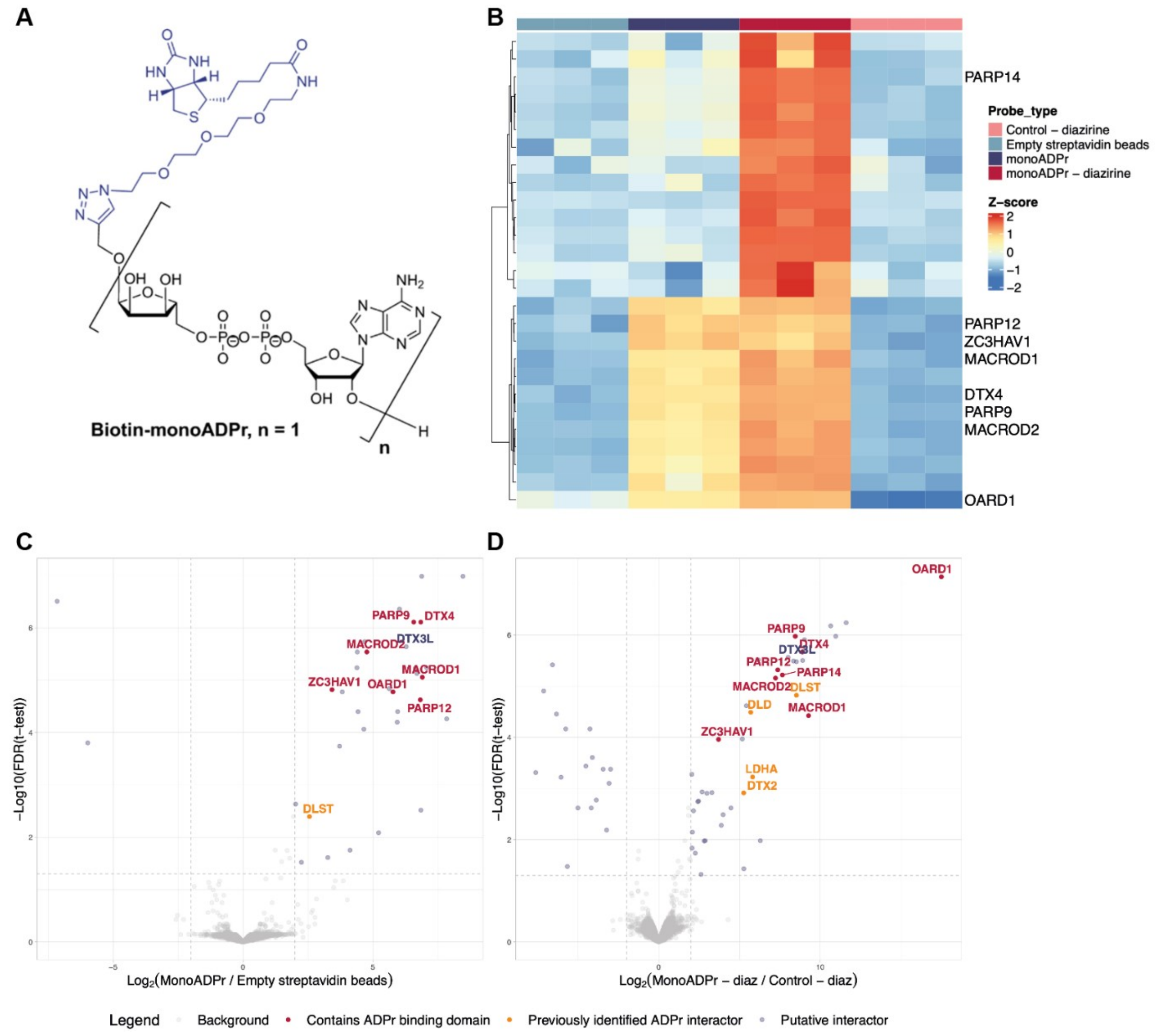

Figure S2. Direct comparison of compound **1a** (mono-ADP-ribose – diazirine) to the mono-ADP-ribose probe previously described.[1] (A) Molecular structure of the previously synthesized mono-ADP-ribose affinity probe. (B) Hierarchical clustering of significantly enriched proteins (ANOVA, Benjamini-Hochberg correction, FDR 0.05). Proteins displayed with an annotated ADP-ribose binding domain. (C-D) Volcano plots depicting preferential binding of proteins to the previously synthesized mono-ADP-ribose probe versus empty streptavidin beads (as control), or compound **1a** versus compound **2a**, respectively. The statistical cutoffs in the t-test are as follows: FDR < 0.05 and Log_2 fold change (FC) ≥ 2. Proteins containing an ADP-ribose binding domain are indicated in red, proteins that have been identified by at least 2 interactomics methods are indicated in orange.[1–4] (C) and (D) share the same legend.

Table S1. Probable mono-ADPr interactors identified by photo-affinity proteomics. Proteins were classified as probable mono-ADPr interactors by integrating enrichment data from non-crosslinked and photo-crosslinked pull-downs. “No UV” indicates proteins enriched with either the previously synthesized monoADPr probe (short linker, no diazirine) and/or the monoADPr-diazirine probe (compound 1a) under non-crosslinking conditions (FC > 1.5, see Figure S2). “1 min UV” and “20 min UV (rep)” refer to photo-affinity labelling with compound 1a irradiated for 1 or 20 minutes, respectively (FC > 3; for 20 minutes only those significantly enriched in both replicates were included).

Group	Gene	No UV	1 min UV	20 min UV (both replicates)	Annotation
Group 1 – Detected under all conditions (highest confidence)	DLD	✓	✓	✓	Reported in multiple ADPr studies
	MACROD1	✓	✓	✓	ADPr-binding domain
	ME2	✓	✓	✓	
	OARD1	✓	✓	✓	ADPr-binding domain
	PARP12	✓	✓	✓	ADPr-binding domain
	PARP14	✓	✓	✓	ADPr-binding domain
	PARP9	✓	✓	✓	ADPr-binding domain
Group 2 – Detected under both UV conditions	ACTR3		✓	✓	
	ADH5		✓	✓	
	ADPRS		✓	✓	
	ALDH2		✓	✓	
	ALDH5A1		✓	✓	
	CLPB		✓	✓	
	CTBP1		✓	✓	
	CTBP2		✓	✓	
	FAM20B		✓	✓	
	FN3K		✓	✓	
	MAP2K4		✓	✓	
	NAGK		✓	✓	
	NUDT9		✓	✓	
	PDE12		✓	✓	
	SIRT1		✓	✓	
Group 3 – Detected under one UV + non-UV condition	CYB5R1	✓		✓	
	DTX2	✓	✓		ADPr-binding domain + reported in multiple ADPr studies
	DTX3	✓		✓	
	DTX3L	✓	✓		

Group	Gene	No UV	1 min UV	20 min UV (both replicates)	Annotation
	GSS	✓	✓		
	LDHA	✓		✓	Reported in multiple ADPr studies
	OGDH	✓		✓	
	OGFR	✓		✓	
	SIRT4	✓		✓	

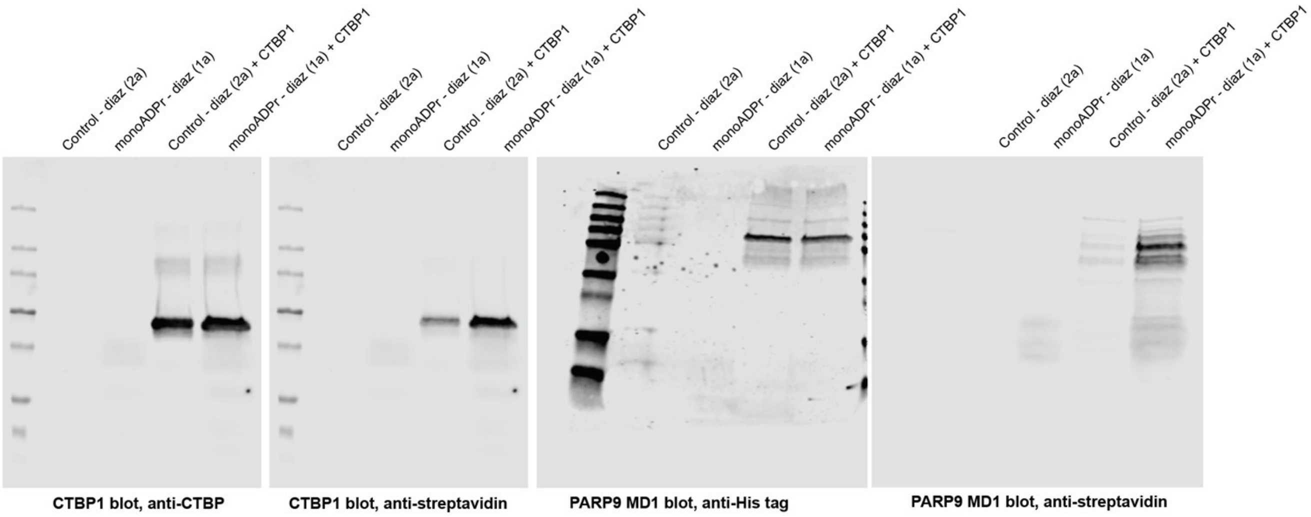

Figure S3 Validation of direct mono-ADP-ribose interaction by CTPB1. Recombinant CTBP1 and PARP9 macrodomain 1: uncropped blots.

2. Experimental Section

General

All chemicals were of reagent grade and were used without further purification unless stated otherwise. All reactions were performed under an N_2 atmosphere unless stated otherwise. Reactions were monitored by analytical thin layer chromatography (TLC) using Merck aluminum sheets pre-coated with silica gel 60Å with detection by UV-absorption (254 nm) and by spraying with a solution of $KMnO_4$ (20 g/L) and $K_{2b}O_3$ (10 g/L) in water, ninhydrin (0.75 g/L) in EtOH or $(NH_4)_6Mo_7O_{24}\cdot 4H_2O$ (25 g/L) and $(NH_4)_{4b}e(SO_4)_4\cdot 2H_2O$ (10 g/L) in 10% sulfuric acid followed by charring. Additional analysis with TLC-MS was used when needed. Column chromatography was performed manually using Macherey-Nagel silica gel 60Å (40-63 µm) or a Biotage IsoleraTM flash purification system using silica gel cartridges (Screening Device SilicaSep HP, particle size 15-40 µm, 60Å) in the indicated solvents. Size exclusion chromatography was performed using SephadexTM (LH-20, GE Healthcare Life Sciences) in MeOH:DCM (1:1, v:v). For HW-40 gel filtration purifications an ÄKTA explorer system equipped with a Superdex-30-HR column (16 mm x 100 cm, flow 1 mL/min) was used. For strong anion exchange chromatography purifications an ÄKTA pure system equipped with a Source-15Q column (16x100 mm, flow 4 mL/min) was used. For reversed-phase preparative HPLC purifications a Gilson HPLC system equipped with a C18 semi-preparative column (Gemini C18, 250x10 mm, 5 µm particle size, Phenomenex, flow 5 mL/min) was used.

NMR spectra were recorded on a Bruker AV-400, AV-500 or AV-600 spectrometer. Chemical shifts are given in ppm (*∂*) relative to the chloroform, dimethylsulfoxide, methanol or deuterium oxide residual solvent peak or tetramethylsilane (TMS) as an internal standard. Coupling constants (*J*) are given in Hz. All given ^{13}C-NMR spectra are proton decoupled. 2D NMR experiments (HSQC, COSY) were carried out to assign protons and carbons of the synthesized structures.

High resolution mass spectra (HRMS) of the new compounds were recorded with a Q-Exactive HF Orbitrap (Thermo Scientific) equipped with an electrospray ion source (ESI) and injection of 2 µl of a 1 µM solution via an Ultimate 3000 nano UPLC (Dionex) system with an external calibration (Thermo Scientific), source voltage of 3,5 kV, capillary temperature of 275 °C, no sheath gas, resolution R = 240.000 at m/z=400 (mass range m/z=160-2000 or until a maximum of 6000) and ACN:H_2O (1:1 v/v) supplemented with 0.1% formic acid as eluent. LC-MS analysis was performed on an LCQ Advantage Max (Thermo Finnigan) ion-trap spectrometer (ESI+) coupled to a Surveyor HPLC system (Thermo Finnigan) equipped with a C18 column (Gemini, 4.6 mm x 50 mm, 3 µM particle size, Phenomenex) equipped with buffers A: H_2O, B: acetonitrile (ACN), C: 1% TFA or on an Agilent technologies 1260 infinity LC-MS with a 6120 Quadrupole MS system equipped with buffers A: H_2O, B: acetonitrile (ACN) and C: 100 mM NH_4OAc.

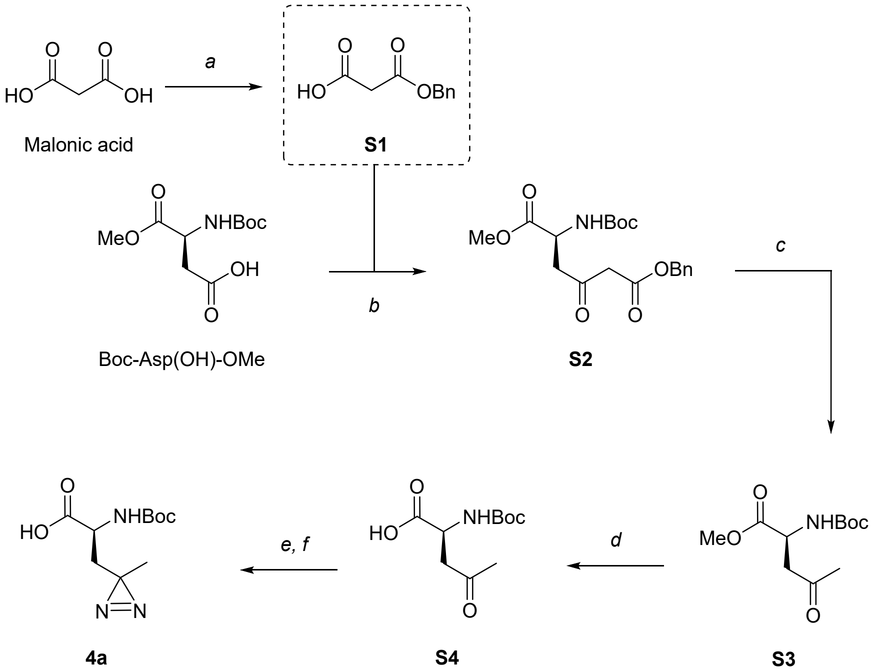

Scheme S1. Synthesis of Boc-L-photo-leucine **4a** according to the procedures of Yang *et al.*[5] Reagents and conditions: a) BnBr, Et_3N, ACN, reflux, 4.5 h, 35%. b) *i.* Boc-Asp(OH)-OMe, CDI, THF, rt, 3.5 h. *ii.* **S1**, iPrMgCl, THF, 0 °C → 50 °C, 1 h; *then* activated Boc-Asp(OH)-OMe anhydride, 0 °C → rt, overnight, 84%. c) Pd/C, H_2 (g), MeOH, rt, overnight; *then* 50 °C, 6 h, 55%. d) LiOH, H_2O/THF, rt, 1 h, quant. e) NH_3, -30 to 40 °C, 6 h; *then* hydroxylamine-*O*-sulfonic acid, MeOH, -50 ° C → -30 to -40 °C, overnight. f) Et_3N, I_2, MeOH, 0 °C → rt, 2 h, 12% over 2 steps.

3-(Benzyloxy)-3-oxopropanoic acid (S1) [5]

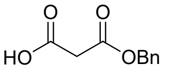

Malonic acid (6.55 g, 62.94 mmol) was dissolved in ACN (100 mL). Et_3N (5.6 mL, 62.94 mmol, 1.0 eq) and BnBr (5.0 mL, 62.94 mmol, 1.0 eq) were added and the reaction mixture was refluxed for 4.5 hours. After complete conversion, aq. HCl (1 M) was slowly added at 0 °C until pH 3-4 and the aqueous phase was extracted with EtOAc (3x). The combined organic layers were concentrated under reduced pressure and the resulting residue was diluted with sat. aq. $NaHCO_3$. Next, the aqueous phase was washed with Et_2O (3x). After complete removal of the dibenzyl malonate, aq. HCl (1 M) was slowly added at 0 °C until pH 3-4 and the aqueous phase was extracted with EtOAc (3x). The combined organic layers were dried over $MgSO_4$, filtered and concentrated under reduced pressure and crude β-keto acid **S1** (4.32 g, 22.25 mmol, 35%) was used in the next step without further purification. **^{1}H NMR (400 MHz, DMSO)** δ 7.42 – 7.30 (m, 5H, arom.), 5.14 (s, 2H, CH_2 Bn), 3.45 (s, 2H, $CH_2\alpha$). **^{13}C NMR (101 MHz, DMSO)** δ 168.1, 166.9 (CO carboxylic acid, CO ester), 135.9 (Cq. arom.), 128.5, 128.2, 128.0 (arom.), 66.1 (CH_2 Bn), 41.6 ($CH_2\alpha$).

(S)-6-Benzyl 1-methyl 2-((*tert*-butoxycarbonyl)amino)-4-oxohexanedioate (S2) [5]

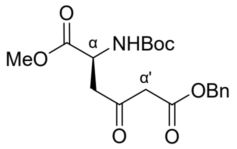

β-keto acid **S1** (1.73 g, 8.90 mmol, 1.1 eq) was dissolved in dry THF (6 mL) and the resulting solution was purged with Ar. iPrMgCl (2 M in THF, 8.9 mL, 17.80 mmol, 2.2 eq) was added dropwise at 0 °C and the reaction mixture was stirred at the same temperature for 30 minutes. Then, the reaction mixture was stirred at 50 °C for 30 minutes until complete conversion into the enolate. Boc-Asp(OH)-OMe (2.00 g, 8.09 mmol, 1.0 eq) was dissolved in dry THF (11 mL). CDI (1.44 g, 8.90 mmol, 1.1 eq) was added portionwise and the reaction mixture was stirred at room temperature for 3.5 hours. After complete conversion into the anhydride, the resulting mixture was slowly added to the flask containing the enolate at 0 °C and the reaction mixture was stirred at room temperature overnight. Then, the reaction was quenched with aq. HCl (0.1 M) and the aqueous phase was extracted with EtOAc (3x). The combined organic layers were washed with brine (1x), dried over

$MgSO_4$, filtered and concentrated under reduced pressure. Purification by silica gel column chromatography (0% → 30% EtOAc in pentane) afforded β-keto ester **S2** as a white solid (2.57 g, 6.78 mmol, 84%). **^{1}H NMR (400 MHz, $CDCl_3$)** δ 7.37 – 7.34 (m, 5H, arom.), 5.47 (d, *J* = 8.5 Hz, 1H, NH), 5.17 (s, 2H, CH_2 Bn), 4.53 (dt, *J* = 8.6, 4.4 Hz, 1H, CHα), 3.70 (s, 3H, CH_3 OMe), 3.51 (s, 2H, CH_2α'), 3.25 (AB, *J* = 18.3, 4.5 Hz, 1H, CH_2), 3.08 (AB, *J* = 18.3, 4.4 Hz, 1H, CH_2), 1.44 (s, 9H, CH_3 Boc). **^{13}C NMR (101 MHz, $CDCl_3$)** δ 200.8 (CO ketone), 171.6 (CO OMe ester), 166.5 (CO OBn ester), 155.5 (CO Boc), 135.2 (Cq. arom.), 128.7, 128.6, 128.5 (arom.), 80.2 (Cq. Boc), 67.4 (CH_2 Bn), 52.8 (CH_3 OMe), 49.4 (CHα), 49.1 (CH_2α'), 45.0 (CH_2), 28.4 (CH_3 Boc). Spectral data were in accordance with those reported in the literature. [5]

(S)-Methyl 2-((*tert*-butoxycarbonyl)amino)-4-oxopentanoate (S3) [5]

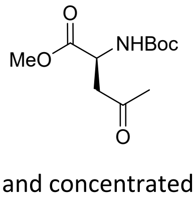

β-keto ester **S2** (2.57 g, 6.78 mmol) was dissolved in MeOH (135 mL). Pd/C (10 wt%, 0.257 g, 0.24 mmol, 0.035 eq.) was added and the reaction mixture was stirred at room temperature under constant bubbling of H_2 (g) for 5 hours until complete removal of the benzyl ester. Then, the reaction mixture was stirred at room temperature overnight followed by for 6 hours at 50 °C until complete conversion into the ketone. The reaction mixture was filtered over Celite and concentrated under reduced pressure. Purification by silica gel column chromatography (10% → 30% EtOAc in pentane) afforded ketone **S3** as an oil (0.92 g, 3.73 mmol, 55%). **^{1}H NMR (400 MHz, $CDCl_3$)** δ 5.50 (d, *J* = 8.6 Hz, 1H, NH), 4.49 (dt, *J* = 8.6, 4.2 Hz, 1H, CHα), 3.73 (s, 3H, CH_3 OMe), 3.22 – 3.16 (m, 1H, CH_2), 2.96 (AB, *J* = 18.3, 4.3 Hz, 1H, CH_2), 2.17 (s, 3H, CH_3 Me), 1.45 (s, 9H, CH_3 Boc). **^{13}C NMR (101 MHz, $CDCl_3$)** δ 206.7 (CO ketone), 172.0 (CO OMe ester), 155.6 (CO Boc), 80.1 (Cq. Boc), 52.7 (CH_3 OMe), 49.5 (CHα), 45.5 (CH_2), 30.0 (CH_3 Me), 28.4 (CH_3 Boc). Spectral data were in accordance with those reported in the literature. [5]

(S)-2-((*Tert*-butoxycarbonyl)amino)-4-oxopentanoic acid (S4) [5]

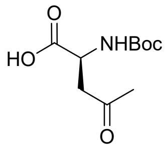

Ester **S3** (0.92 g, 3.73 mmol) was dissolved in THF (9.4 mL). Aq. LiOH (1 M, 7.5 mL, 7.47 mmol, 2.0 eq) was slowly added and the reaction mixture was stirred at room temperature for 1 hour. After complete conversion, aq. HCl (0.1 M) was slowly added at 0 °C until pH 3-4 and the aqueous phase was extracted with EtOAc (5x). The combined organic layers were washed with brine (1x), dried over $MgSO_4$, filtered and concentrated under reduced pressure and crude carboxylic acid **S4** (0.90 g, 3.73 mmol, quantitative) was used in the next step without further purification. **^{1}H NMR (400 MHz, $CDCl_3$)** δ 9.76 (bs, 1H, OH carboxylic acid), 5.56 (d, *J* = 8.5 Hz, 1H, NH), 4.53 (dt, *J* = 8.6, 4.5 Hz, 1H, CHα), 3.21 (AB, *J* = 18.3, 3.8 Hz, 1H, CH_2), 2.97 (AB, *J* = 18.3, 4.3 Hz, 1H, CH_2), 2.19 (s, 3H, CH_3 Me), 1.44 (s, 9H, CH_3 Boc). **^{13}C NMR (101 MHz, $CDCl_3$)** δ 207.2 (CO ketone), 176.2 (CO carboxylic acid), 155.9 (CO Boc), 80.5 (Cq. Boc), 49.4 (CHα), 45.3 (CH_2), 30.0 (CH_3 Me), 28.4 (CH_3 Boc). Spectral data were in accordance with those reported in the literature. [5]

(S)-2-((*Tert*-butoxycarbonyl)amino)-3-(3-methyl-3H-diazirin-3-yl)propanoic acid (4a) [5]

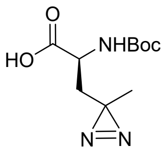

Ketone **S4** (0.90 g, 3.73 mmol) was dissolved in liquid ammonia (18.7 mL) at -30 to -40 °C. The resulting mixture was stirred at the same temperature for 6 hours. After conversion into the imine, hydroxylamine-O-sulfonic acid (0.51 g, 4.49 mmol, 1.2 eq) was dissolved in dry MeOH (6 mL), added dropwise at -50 °C and the reaction mixture was stirred at -30 to -40 °C overnight. Then, ammonia was evaporated by allowing the reaction mixture to reach room temperature and the resulting mixture was filtered over Celite and concentrated under reduced pressure. Without further purification, the crude diaziridine was dissolved in dry MeOH (8.7 mL) and covered from light. Et_3N (1.04 mL, 7.48 mmol, 2.0 eq) and I_2 (1.35 M in dry MeOH, 4 mL, 5.39 mmol, 1.44 eq) were added at 0 °C and the reaction mixture was stirred at room temperature for 2 hours. Then, the reaction mixture was concentrated under reduced pressure, diluted with EtOAc and the organic phase was washed with H_2O (1x). Aq. HCl (1 M) was slowly added to the aqueous phase until pH 1 and extracted with DCM (10x). The combined organic layers were washed with

brine (1x), dried over $MgSO_4$, filtered and concentrated under reduced pressure. Purification by silica gel column chromatography (0% → 20% EtOAc in pentane) afforded diazirine **4a** as a colorless liquid (0.11 g, 0.45 mmol, 12% over 2 steps). **^{1}H NMR (400 MHz, MeOD)** δ 4.04 (dd, *J* = 10.3, 4.5 Hz, 1H, CHα), 1.93 (AB, *J* = 14.9, 4.5 Hz, 1H, CH_2), 1.64 – 1.58 (m, 1H, CH_2), 1.47 (s, 9H, CH_3 Boc), 1.05 (s, 3H, CH_3 Me). **^{13}C NMR (101 MHz, MeOD)** δ 175.1 (CO carboxylic acid), 157.8 (CO Boc), 80.6 (Cq. Boc), 51.1 (CHα), 37.8 (CH_2), 28.7 (CH_3 Boc), 25.0 (Cq. diazirine), 19.9 (CH_3 Me). Spectral data were in accordance with those reported in the literature. [5]

General procedure A = Amide formation

Azidopropylamine (66 mg, 0.66 mmol, 2.45 eq) was dissolved in DCM (0.66 mL). The resulting solution was added to a flask containing carboxylic acid **4b** (100 mg, 0.27 mmol, 1.00 eq), HOBt·H_2O (wetted with not less than 14 wt. % water, 47 mg, 0.30 mmol, 1.10 eq) and EDC·HCl (57 mg, 0.30 mmol, 1.10 eq) in DCM (0.5 mL) and the reaction mixture was stirred at room temperature overnight covered from light. Then, the reaction was carefully quenched with 10 wt. % aq. citric acid. The aqueous phase was extracted with DCM (3x) and the combined organic layers were washed with 10 wt. % aq. citric acid (1x), sat. aq. $NaHCO_3$ (1x) and brine (1x). Next, the organic layer was dried over $MgSO_4$, filtered and concentrated under reduced pressure. Purification by silica gel column chromatography (70% Et_2O in pentane) afforded amide **5b** as a light yellow oil (90 mg, 0.20 mmol, 74%).

General procedure B = Boc deprotection

Carbamate **5a** (44 mg, 0.14 mmol) was dissolved in a mixture of 4M HCl in dioxane (2 mL) and the reaction mixture was stirred at room temperature covered from light until full conversion was confirmed by TLC (1 – 3 hours). Then, the reaction mixture was concentrated under reduced pressure, co-evaporated with toluene (3x) and the crude amine as HCl salt was used in the next step without further purification.

General procedure C = Amide formation

The HCl salt of amine **6b** (52 mg, 0.14 mmol, 1.00 eq) was dissolved in DCM (0.5 mL), neutralized using DiPEA (71 µL, 0.41 mmol, 3.00 eq.) and covered from light. The resulting solution was added to a flask containing biotin-PEG_4-acid **7** (66 mg, 0.14 mmol, 1.00 eq), HOBt·H_2O (wetted with not less than 14 wt. % water, 23 mg, 0.15 mmol, 1.10 eq) and EDC·HCl (28 mg, 0.15 mmol, 1.10 eq) in DCM (0.7 mL) and the reaction mixture was stirred at room temperature overnight. Then, the reaction mixture was concentrated under a flow of nitrogen. Purification by silica gel column chromatography (0% → 8% MeOH in DCM) followed by size exclusion chromatography (MeOH:DCM (1:1, v:v)) afforded amide **2b** as a clear oil (88 mg, 0.11 mmol, 79%).

General procedure D = CuAAC Click Chemistry

1-*O*-Propargyl-α-mono-ADP-ribose **3** [6] (1 mg, 1.67 µmol, 1.00 eq) was dissolved in H_2O (100 µL) and covered from light. Biotinylated photo-crosslinker **2a** (0.044 M in ACN, 63 µL, 2.77 µmol, 1.66 eq) was added and the mixture was diluted with additional H_2O (250 µL). Then, pre-mixed click cocktail (1:1:1 v/v/v, $CuSO_4$ (26 mg/mL in H_2O): sodium ascorbate (120 mg/mL in H_2O): THPTA (44 mg/mL in H_2O), 60 µL) was added and the reaction mixture was shaken at room temperature until LC-MS analysis indicated complete conversion (1 – 19 hours). Next, the reaction mixture was quenched with EDTA (0.5 M in H_2O, 15 µL) and purification by reversed-phase preparative HPLC (A: 50 mM NH_4OAc in milliQ, B: acetonitrile, gradient: 10-50% B in 15 min) afforded probe **1a** as a white solid (0.47 mg, 0.36 µmol, 22%) after repeated lyophilization.

Boc-L-photo-leucine-3-azidopropanamine (5a)

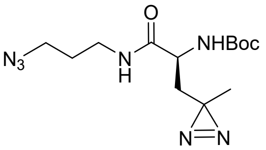

Azidopropylamine (55 mg, 0.55 mmol, 2.45 eq) was dissolved in DCM (0.5 mL) and reacted with carboxylic acid **4a** (55 mg, 0.23 mmol, 1.00 eq), HOBt·H_2O (wetted with not less than 14 wt. % water, 39 mg, 0.25 mmol, 1.1 eq) and EDC·HCl (48 mg, 0.25 mmol, 1.1 eq) in DCM (0.5 mL) according to general procedure A covered from light to obtain the title compound. Purification by silica gel column chromatography (0% → 40% EtOAc in pentane) afforded amide **5a** as a yellow oil (44 mg, 0.14 mmol, 59%). **^{1}H NMR (400 MHz, $CDCl_3$)** δ 6.82 – 6.72 (m, 1H, NH propyl), 5.15 (d, *J* = 7.3 Hz, 1H, NH photo-Leu), 4.09 – 3.96 (m, 1H, CHα photo-Leu), 3.39 – 3.32 (m, 4H, CH_2 propyl), 2.09 – 2.01 (m, 1H, CH_2 photo-Leu), 1.80 (p, *J* = 6.6 Hz, 2H, CH_2 propyl), 1.56 (AB, *J* = 15.6, 9.3 Hz, 1H, CH_2 photo-Leu), 1.47 (s, 9H, CH_3 Boc), 1.07 (s, 3H, CH_3 photo-Leu). **^{13}C NMR (101 MHz, $CDCl_3$)** δ 171.4 (CO amide), 155.7 (CO Boc), 80.8 (Cq. Boc), 51.0 (CHα photo-Leu), 49.2, 37.2 (CH_2 propyl), 37.1 (CH_2 photo-Leu), 28.7 (CH_2 propyl), 28.4 (CH_3 Boc), 23.9 (Cq. diazirine), 20.0 (CH_3 photo-Leu). **HRMS** (ESI) $[M+Na]^+$ calculated for $C_{13}H_{23}N_7O_3Na$ 348.17546; found 348.17520.

Boc-4-benzoyl-L-phenylalanine-3-azidopropanamine (5b)

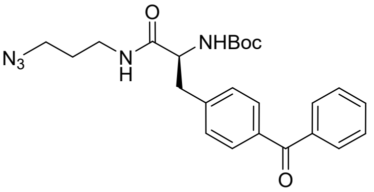

Azidopropylamine (66 mg, 0.66 mmol, 2.45 eq) was dissolved in DCM (0.66 mL) and reacted with carboxylic acid **4b** (100 mg, 0.27 mmol, 1.00 eq), HOBt·H_2O (wetted with not less than 14 wt. % water, 47 mg, 0.30 mmol, 1.10 eq) and EDC·HCl (57 mg, 0.30 mmol, 1.10 eq) in DCM (0.5 mL) according to general procedure A covered from light to obtain the title compound. Purification by silica gel column chromatography (70% Et_2O in pentane) afforded amide **5b** as a light yellow oil (90 mg, 0.20 mmol, 74%). **^{1}H NMR (400 MHz, $CDCl_3$)** δ 7.77 – 7.71 (m, 4H, arom.), 7.58 (tt, *J* = 7.0, 1.2 Hz, 1H, arom.), 7.46 (t, *J* = 7.6 Hz, 2H, arom.), 7.33 (d, *J* = 8.2 Hz, 2H, arom.), 6.86 – 6.48 (m, 1H, NH propyl), 5.43 (d, *J* = 8.3 Hz, 1H, NH 4-Bz-Phe), 4.53 – 4.34 (m, 1H, CHα 4-Bz-Phe), 3.31 – 3.14 (m, 5H, CH_2 propyl, CH_2 4-Bz-Phe), 3.09 (AB, *J* = 13.3, 7.3 Hz, 1H, CH_2 4-Bz-Phe), 1.69 (p, *J* = 6.6 Hz, 2H, CH_2 propyl), 1.40 (s, 9H, CH_3 Boc). **^{13}C NMR (101 MHz, $CDCl_3$)** δ 196.4 (CO ketone), 171.4 (CO amide), 155.6 (CO Boc), 142.0, 137.5, 136.2 (Cq. arom.), 132.5, 130.4, 130.0, 129.4, 128.3 (arom.), 80.4 (Cq. Boc), 55.7 (CHα 4-Bz-Phe), 49.0 (CH_2 propyl), 38.6 (CH_2 4-Bz-Phe), 37.0, 28.6 (CH_2 propyl), 28.3 (CH_3 Boc). **HRMS** (ESI) $[M+H]^+$ calculated for $C_{24}H_{30}N_5O_4$ 452.22923; found 452.22995.

H-L-Photo-leucine-3-azidopropanamine (hydrochloride) (6a)

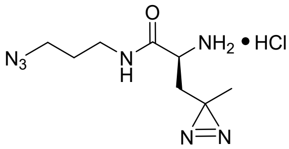

Carbamate **5a** (44 mg, 0.14 mmol) was reacted with 4M HCl in dioxane (2 mL) according to general procedure B covered from light to obtain the title compound. The crude amine as HCl salt was used in the next step without further purification.

H-4-Benzoyl-L-phenylalanine-3-azidopropanamine (hydrochloride) (6b)

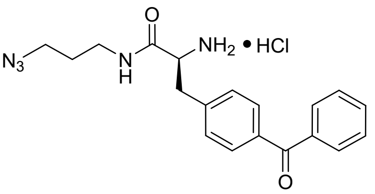

Carbamate **5b** (61 mg, 0.14 mmol) was reacted with 4M HCl in dioxane (0.5 mL) according to general procedure B covered from light to obtain the title compound. The crude amine as HCl salt was used in the next step without further purification.

Biotin-PEG$_4$-L-photo-leucine-3-azidopropanamine (2a)

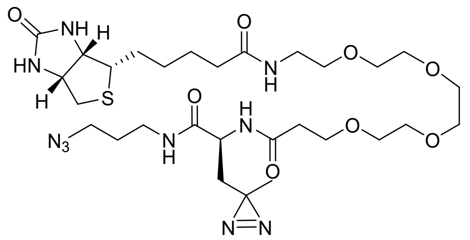

The HCl salt of amine **6a** (35 mg, 0.14 mmol, 1.00 eq) was dissolved in DCM (0.5 mL), neutralized using DiPEA (71 µL, 0.41 mmol, 3.00 eq.) and reacted with biotin-PEG$_4$-acid **7** (66 mg, 0.14 mmol, 1.00 eq), HOBt·H_2O (wetted with not less than 14 wt. % water, 24 mg, 0.15 mmol, 1.10 eq) and EDC·HCl (29 mg, 0.15 mmol, 1.10 eq) in DCM (0.5 mL) according to general procedure C covered from light to obtain the title compound. Purification by silica gel column chromatography (0% → 10% MeOH in DCM) afforded amide **2a** as a yellow oil (52 mg, 0.07 mmol, 55%) containing 1 eq of HOBt for which the yield reported was corrected. **^{1}H NMR (400 MHz, CDCl$_3$)** δ 7.67 (d, *J* = 7.9 Hz, 1H, NH photo-Leu), 7.61 (t, *J* = 5.4 Hz, 1H, NH propyl), 6.96 (t, *J* = 5.7 Hz, 1H, NH biotin), 6.64 (s, 1H, NH carbamide), 5.76 (s, 1H, NH carbamide), 4.53 (dd, *J* = 7.6, 4.9 Hz, 1H, CH biotin), 4.44 (td, *J* = 8.7, 5.9 Hz, 1H, CHα photo-Leu), 4.34 (dd, *J* = 7.2, 5.1 Hz, 1H, CH biotin), 3.80 – 3.75 (m, 2H, CH_2 PEG), 3.65 – 3.62 (m, 12H, CH_2 PEG), 3.58 (t, *J* = 5.0 Hz, 2H, CH_2 PEG), 3.45 – 3.41 (m, 2H, CH_2 PEG), 3.40 – 3.29 (m, 4H, CH_2 propyl), 3.17 – 3.13 (m, 1H, CH-S), 2.91 (AB, *J* = 12.8, 4.8 Hz, 1H, CH_2-S), 2.75 (d, *J* = 12.8 Hz, 1H, CH_2-S), 2.58 – 2.51 (m, 2H, CH_2 PEG), 2.20 (t, *J* = 7.5 Hz, 2H, CH_2 biotin), 2.04 (AB, *J* = 14.9, 5.8 Hz, 1H, CH_2 photo-Leu), 1.79 (p, *J* = 6.7 Hz, 2H, CH_2 propyl), 1.73 – 1.61 (m, 4H, CH_2 biotin), 1.56 (AB, *J* = 14.6, 8.7 Hz, 1H, CH_2 photo-Leu), 1.46 – 1.43 (m, 2H, CH_2 biotin), 1.06 (s, 3H, CH_3 photo-Leu). **^{13}C NMR (101 MHz, CDCl$_3$)** δ 173.5, 172.0, 171.4 (CO amide), 164.2 (CO carbamide), 70.5, 70.5, 70.4, 70.3, 70.1, 70.0, 67.3 (CH_2 PEG), 62.0, 60.3 (CH biotin), 55.8 (CH-S), 49.7 (CHα photo-Leu), 49.1 (CH_2 propyl), 40.6 (CH_2-S), 39.3 (CH_2 PEG), 36.8 (CH_2 propyl), 36.8 (CH_2 photo-Leu), 35.8 (CH_2 biotin), 28.6 (CH_2 propyl), 28.2, 28.2, 25.5 (CH_2 biotin), 24.0 (Cq. diazirine), 19.8 (CH_3 photo-Leu). **HRMS** (ESI) $[M+H]^+$ calculated for $C_{29}H_{51}N_{10}O_8S$ 699.36066; found 699.36070.

Biotin-PEG$_4$-4-benzoyl-L-phenylalanine-3-azidopropanamine (2b)

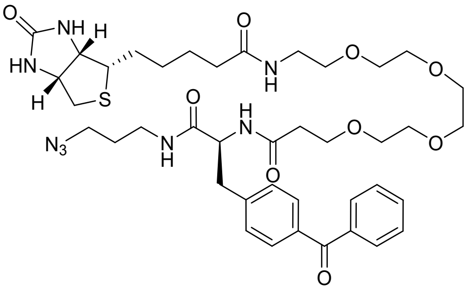

The HCl salt of amine **6b** (52 mg, 0.14 mmol, 1.00 eq) was dissolved in DCM (0.5 mL), neutralized using DiPEA (71 µL, 0.41 mmol, 3.00 eq.) and reacted with biotin-PEG$_4$-acid **7** (66 mg, 0.14 mmol, 1.00 eq), HOBt·H_2O (wetted with not less than 14 wt. % water, 23 mg, 0.15 mmol, 1.10 eq) and EDC·HCl (28 mg, 0.15 mmol, 1.10 eq) in DCM (0.7 mL) according to general procedure C covered from light to obtain the title compound. Purification by silica gel column chromatography (0% → 8% MeOH in DCM) followed by size exclusion chromatography (MeOH:DCM (1:1, v:v)) afforded amide **2b** as a clear oil (88 mg, 0.11 mmol, 79%). **^{1}H NMR (400 MHz, CDCl$_3$)** δ 7.78 – 7.71 (m, 5H, NH 4-Bz-Phe, arom.), 7.61 – 7.56 (m, 1H, arom.), 7.48 (t, *J* = 7.6 Hz, 2H, arom.), 7.43 – 7.35 (m, 3H, NH propyl, arom.), 6.98 (t, *J* = 5.3 Hz, 1H, NH biotin), 6.70 (s, 1H, NH carbamide), 5.83 (d, *J* = 11.0 Hz, 1H, NH carbamide), 4.76 (q, *J* = 7.6 Hz, 1H, CHα 4-Bz-Phe), 4.54 – 4.49 (m, 1H, CH biotin), 4.35 – 4.30 (m, 1H, CH biotin), 3.68 – 3.55 (m, 16H, CH_2 PEG), 3.44 – 3.38 (m, 2H, CH_2 PEG), 3.31 – 3.07 (m, 7H, CH_2 propyl, CH_2 4-Bz-Phe, CH-S), 2.90 (AB, *J* = 12.8, 4.7 Hz, 1H, CH_2-S), 2.74 (d, *J* = 12.8 Hz, 1H, CH_2-S), 2.54 – 2.41 (m, 2H, CH_2 PEG), 2.18 (t, *J* = 7.6 Hz, 2H, CH_2 biotin), 1.74 – 1.59 (m, 6H, CH_2 biotin, CH_2 propyl), 1.48 – 1.38 (m, 2H, CH_2 biotin). **^{13}C NMR (101 MHz, CDCl$_3$)** δ 196.4 (CO ketone), 173.4, 171.9, 171.5 (CO amide), 164.2 (CO carbamide), 142.3, 137.6, 136.1 (Cq. arom.), 132.5, 130.3, 130.0, 129.4, 128.4 (arom.), 70.5, 70.5, 70.5, 70.4, 70.2, 70.0, 67.3 (CH_2 PEG), 62.0, 60.2 (CH biotin), 55.8 (CH-S), 54.4 (CHα 4-Bz-Phe), 48.9 (CH_2 propyl), 40.7 (CH_2-S), 39.3 (CH_2 PEG), 38.0 (CH_2 4-Bz-Phe), 36.8 (CH_2 PEG), 36.7 (CH_2 propyl), 35.8 (CH_2 biotin), 28.6 (CH_2 propyl), 28.3, 28.2, 25.5 (CH_2 biotin). **HRMS** (ESI) $[M+H]^+$ calculated for $C_{40}H_{57}N_8O_9S$ 825.39637; found 825.39774.

Biotin-PEG$_4$-L-photo-leucine-propanamine-α-mono-ADP-ribose (1a)

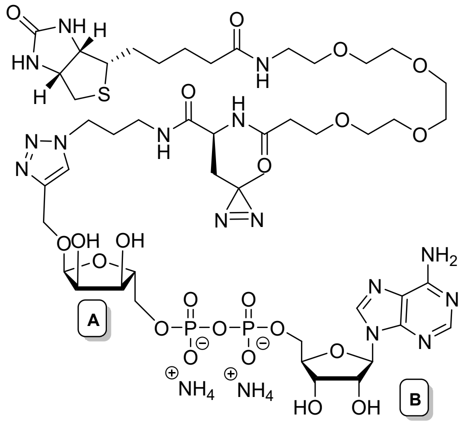

1-*O*-Propargyl-α-mono-ADP-ribose **3** [6] (1 mg, 1.67 µmol, 1.00 eq) was dissolved in H_2O (100 µL) and reacted with biotinylated photo-crosslinker **2a** (0.044 M in ACN, 63 µL, 2.77 µmol, 1.66 eq) upon addition of H_2O (250 µL) and pre-mixed click cocktail (1:1:1 v/v/v, $CuSO_4$ (26 mg/mL in H_2O): sodium ascorbate (120 mg/mL in H_2O): THPTA (44 mg/mL in H_2O), 60 µL) according to general procedure D covered from light to obtain the title compound. Purification by reversed-phase preparative HPLC (A: 50 mM NH_4OAc in milliQ, B: acetonitrile, gradient: 10-50% B in 15 min) afforded probe **1a** as a white solid (0.47 mg, 0.36 µmol, 22%) after repeated lyophilization. **^{1}H NMR (600 MHz, D_2O)** δ 8.50 (s, 1H, H2-B), 8.21 (s, 1H, H8-B), 7.93 (s, 1H, CH triazole), 6.11 (d, *J* = 6.0 Hz, 1H, H1'-B), 5.05 (d, *J* = 4.3 Hz, 1H, H1-A), 4.75 – 4.74 (m, 1H, H2'-B), 4.71 – 4.69 (m, 1H, CH_2 triazole), 4.61 – 4.54 (m, 2H, CH_2 triazole , CH biotin), 4.51 (dd, *J* = 5.0, 3.4 Hz, 1H, H3'-B), 4.45 – 4.32 (m, 4H, CH_2 propyl, CH biotin, H4'-B), 4.26 – 4.13 (m, 4H, H4-A, H5'-B, CHα photo-Leu), 4.13 – 4.06 (m, 2H, H2-A, H3-A), 4.05 – 3.97 (m, 2H, H5-A), 3.80 (t, *J* = 6.1 Hz, 2H, CH_2 PEG), 3.67 – 3.62 (m, 12H, CH_2 PEG), 3.60 (t, *J* = 5.3 Hz, 2H, CH_2 PEG), 3.36 (t, *J* = 5.4 Hz, 2H, CH_2 PEG), 3.28 (dt, *J* = 9.7, 5.3 Hz, 1H, CH-S), 3.23 – 3.15 (m, 2H, CH_2 propyl), 2.95 (AB, *J* = 13.0, 5.0 Hz, 1H, CH_2-S), 2.74 (d, *J* = 13.1 Hz, 1H, CH_2-S), 2.65 – 2.57 (m, 2H, CH_2 PEG), 2.24 (t, *J* = 7.3 Hz, 2H, CH_2 biotin), 2.10 (p, *J* = 6.9 Hz, 2H, CH_2 propyl), 1.98 – 1.95 (m, 1H, CH_2 photo-Leu), 1.70 – 1.52 (m, 5H, CH_2 photo-Leu, CH_2 biotin), 1.40 – 1.33 (m, 2H, CH_2 biotin), 1.03 (s, 3H, CH_3 photo-Leu). **^{13}C NMR (151 MHz, D_2O)** δ 177.8, 174.9, 173.8 (CO amide), 166.2 (CO carbamide), 156.5 (C4-B), 153.7 (C8-B), 140.7 (C2-B), 125.8 (CH triazole), 119.5 (C5-B), 102.3 (C1-A), 87.6 (C1'-B), 84.8 (C4'-B), 84.2 (C4-A), 75.2 (C2'-B), 71.8 (C2-A), 71.3 (C3'-B), 70.5, 70.5, 70.4 (CH_2 PEG), 70.4 (C3-A), 70.3, 69.7, 67.4 (CH_2 PEG), 66.5 (C5-A), 66.1 (C5'-B), 62.9 (CH biotin), 61.3 (CH_2 triazole), 61.1 (CH biotin), 56.2 (CH-S), 50.9 (CHα photo-Leu), 48.7 (CH_2 propyl), 40.6 (CH_2-S), 39.8 (CH_2 PEG), 37.3 (CH_2 propyl), 36.7 (CH_2 photo-Leu), 36.6 (CH_2 PEG), 36.3 (CH_2 biotin), 29.7 (CH_2 propyl), 28.7, 28.5, 26.0 (CH_2 biotin), 25.2 (Cq. diazirine), 19.6 (CH_3 photo-Leu). **^{31}P NMR (202 MHz, D_2O)** δ -10.65 (pyrophosphate). **LC-MS** (10→50% ACN [0.1% TFA]): Rt = 3.7 min, m/z: 648.6. **HRMS** (ESI) $[M+2H]^{2+}$ calculated for $C_{47}H_{77}N_{15}O_{22}P_2S$ 648.72763; found 648.72755.

Biotin-PEG$_4$-4-benzoyl-L-phenylalanine-propanamine-α-mono-ADP-ribose (1b)

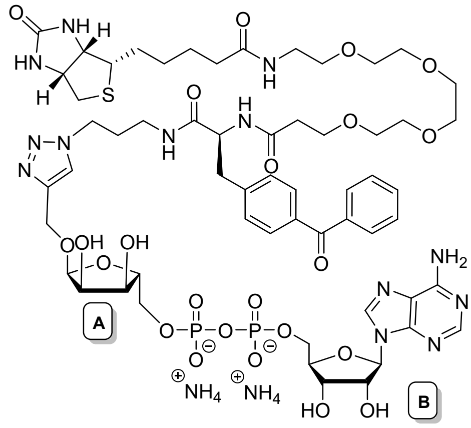

1-*O*-Propargyl-α-mono-ADP-ribose **3** [6] (1.97 mg, 3.12 µmol, 1.00 eq) was dissolved in H_2O (184 µL) and reacted with biotinylated photo-crosslinker **2b** (0.056 M in ACN, 111 µL, 6.24 µmol, 2.00 eq) upon addition of H_2O (468 µL) and pre-mixed click cocktail (1:1:1 v/v/v, $CuSO_4$ (26 mg/mL in H_2O): sodium ascorbate (120 mg/mL in H_2O): THPTA (44 mg/mL in H_2O), 112 µL) according to general procedure D covered from light to obtain the title compound. Purification by HW-40 gel filtration (0.15 M NH_4OAc in 10% ACN/MilliQ) followed by reversed-phase preparative HPLC (A: 50 mM NH_4OAc in MilliQ, B: 75% ACN/MilliQ, gradient: 15-40% B) afforded probe **1b** as a white solid (0.80 mg, 0.55 µmol, 18%) after repeated lyophilization. **^{1}H NMR (500 MHz, D_2O)** δ 8.50 (s, 1H, H2-B), 8.19 (s, 1H, H8-B), 7.84 (s, 1H, CH triazole), 7.73 – 7.66 (m, 3H, arom.), 7.66 – 7.60 (m, 2H, arom.), 7.55 – 7.50 (m, 2H, arom.), 7.43 (d, *J* = 8.5 Hz, 2H, arom.), 6.08 (d, *J* = 5.8 Hz, 1H, H1'-B), 4.99 (d, *J* = 4.1 Hz, 1H, H1-A), 4.71 (t, *J* = 5.5 Hz, 1H, H2'-B), 4.66 – 4.60 (m, 2H, CHα 4-Bz-Phe, CH_2 triazole), 4.57 – 4.52 (m, 2H, CH biotin, CH_2 triazole), 4.52 – 4.50 (m, 1H, H3'-B), 4.37 – 4.33 (m, 2H, CH biotin, H4'-B), 4.24 – 4.20 (m, 2H, H5'-B), 4.17 (m, 1H, H4-A), 4.15 – 4.10 (m, 2H, H2-A, H3-A), 4.10 – 4.05 (m, 2H, CH_2 propyl), 4.05 – 4.02 (m, 2H, H5-A), 3.76 – 3.70 (m, 2H, CH_2 PEG), 3.64 – 3.57 (m, 14H, CH_2 PEG), 3.35 (t, *J* = 5.1 Hz, 2H, CH_2 PEG), 3.29 – 3.22 (m, 2H, CH-S, CH_2 propyl), 3.15 (d, *J* = 7.8 Hz, 2H, CH_2 4-Bz-Phe), 3.08 – 3.03 (m, 1H, CH_2

propyl), 2.92 (AB, J = 13.1, 5.0 Hz, 1H, CH_2-S), 2.72 (d, J = 12.8 Hz, 1H, CH_2-S), 2.60 – 2.52 (m, 2H, CH_2 PEG), 2.21 (t, J = 7.4 Hz, 2H, CH_2 biotin), 1.93 – 1.86 (m, 2H, CH_2 propyl), 1.67 – 1.50 (m, 4H, CH_2 biotin), 1.37 – 1.30 (m, 2H, CH_2 biotin). **^{13}C NMR (126 MHz, D_2O)** δ 199.5 (CO ketone), 176.8, 173.9, 172.5 (CO amide), 164.7 (CO carbamide), 151.4 (C8-B), 148.9 (Cq. triazole), 143.9 (C6-B), 142.4 (Cq. arom.), 140.2 (C2-B), 136.4, 135.3 (Cq. arom.), 133.5, 130.8, 130.1, 129.4, 128.5 (arom.), 124.7 (CH triazole), 101.4 (C1-A), 87.0 (C1'-B), 84.0 (C4'-B), 83.4 (C4-A), 74.5 (C2'-B), 71.0 (C2-A), 70.4 (C3'-B), 69.7, 69.6, 69.6 (CH_2 PEG), 69.5 (C3-A), 69.5, 69.4, 68.9, 66.6 (CH_2 PEG), 65.6 (C5-A), 65.2 (C5'-B), 62.1 (CH biotin), 60.4 (CH_2 triazole), 60.2 (CH biotin), 55.3 (CH-S), 55.0 (CHα 4-Bz-Phe), 47.6 (CH_2 propyl), 39.7 (CH_2-S), 38.9 (CH_2 PEG), 37.3 (CH_2 4-Bz-Phe), 36.2 (CH_2 propyl), 35.7 (CH_2 PEG), 35.4 (CH_2 biotin), 28.8 (CH_2 propyl), 27.9, 27.7, 25.1 (CH_2 biotin). **^{31}P NMR (202 MHz, D_2O)** δ -10.51, -10.62, -10.73, -10.83 (pyrophosphate). **LC-MS** (10→50% ACN [0.1% TFA]): Rt = 5.2 min, m/z: 1422.8. **HRMS** (ESI) $[M+2H]^{2+}$ calculated for $C_{58}H_{83}N_{13}O_{23}P_2S$ 711.74549; found 711.74635.

Cell culture

HeLa (human cervical adenocarcinoma) were maintained at 37°C in a humidified atmosphere containing 5% CO_2, using Dulbecoo's Modified Eagle Medium (DMEM, ThermoFisher) supplemented with 10% fetal bovine serum (FBS, Serana) and 1% Penicillin/Streptomycin (15140-122, ThermoFisher). Cells were routinely checked and tested negative for *Mycoplasma* contamination.

Preparation of whole cell extract

At 80-90% confluency cells were harvested by trypsinization (trypsin, ThermoFisher) and pelleted by centrifuging at 400g for 5 minutes at 4°C. All subsequent steps regarding the lysis were performed at 4°C or on ice. The cell pellet was washed three times with PBS and subsequently lysed in 2.5x the pellet volume of lysis buffer (50mM Tris-HCl pH 8.0, 150mM NaCl, 1.5mM $MgCl_2$, 0.5% NP40, 1µl benzonase (Sigma-Aldrich), 1x Complete Protease Inhibitor Cocktail (#04693132001, Roche), 1x PhosSTOP™ (4906845001, Roche), 1mM DTT, 10µM olaparib (AZD2281, Selleckchem). Resuspended cells were passed five times through a syringe (30G) and incubated in rotation for 30 minutes, followed by 30 minutes centrifugation at 10000 rpm. Supernatant was kept aside in a new tube. The remaining pellet of unlysed cells was resuspended with lysis buffer (0.5x the original pellet volume) and passed 10 times through a 30G syringe. 1µl of benzonase was added and samples were incubated for 30 minutes in rotation, then 30 minutes centrifugation at 10000rpm. Supernatant was added to the tube with the previously collected supernatant. The lysate was filtered (0.22µm filter) and centrifuged again for 10 minutes at 10000rpm. The supernatant was collected, after which EDTA was added to a final concentration of 2mM and glycerol to 10% v/v. Extracts were snapfrozen and stored at -80°C.

UV irradiation and subsequent streptavidin pull-down

Before incubation with the probe and UV irradiation, whole cell extract (1.5mg per replicate) was mixed with binding buffer (50mM Tris-HCl pH 8.0, 150mM NaCl, 2mM EDTA, 0.1% NP40, Complete protease inhibitor (#04693132001, Roche), PhosSTOP™ (4906845001, Roche), and 0.5mM DTT) to a final volume of 500µl, at 4°C (all subsequent steps up until washing at this temperature or on ice). Each sample was prepared in technical triplicates, for label-free quantification (LFQ) purposes. Protein extract mixes were incubated with 3µM of each probe for 70 minutes in rotation in the dark to prevent any residual light exciting the probe. Samples were then UV irradiated for 20 minutes at 365nm using an RMR-600 Photochemical Reactor (Rayonet) and equilibrated on ice afterwards for 5 minutes. Samples were then incubated with streptavidin beads (Cytiva, Streptavidin Sepharose High performance #17511301) for 30 minutes in rotation. Samples were then washed three times with a stringent wash buffer (8M urea, 50mM Tris-HCl pH 8.0, 1% NP40, 0.1%SDS, Complete protease inhibitor, PhosSTOP™), now all at room temperature and 5 minutes in rotation each. This was followed by three washes with binding buffer, each in rotation for 5 minutes as well. Finally, residual salts and detergents were washed

away by four washes of PBS. Bound proteins in each sample were subjected to on-bead trypsin digestion as described in “On-bead digestion”.

For pull-downs performed in filter plates, the final reaction volume was reduced to 150µl while maintaining the same probe concentration (3µM), incubation time and irradiation conditions as in the 500µl reactions. Accordingly, half the amount of streptavidin beads was used per sample. Filter plate wells (MSHVN4550, Merck) were prepared for use by adding 50µl of 70% ethanol and cleared using a vacuum manifold (Multiscreen Vacuum Manifold, Merck), followed by two washes with 200µl binding buffer. Streptavidin beads were added to each well and residual liquid was removed by vacuum. Subsequently, 150µl of UV-irradiated sample was added and incubated for 30min at 450rpm. Wells were washed three times with 200µl wash buffer, four times with 200µl binding buffer, and six times with 200µl PBS. On-bead digestion was performed directly in the filter plate.

To compare compound 1a/2a directly to the previously described mono-ADP-ribose probe, the same steps were performed with the exception of exposing the probes to UV light.[1] Instead of washing with a stringent wash buffer, samples were washed with a mild wash buffer ((50mM Tris-HCl pH 8.0, 150mM NaCl, 2mM EDTA, 0.25% NP40, Complete protease inhibitor (#04693132001, Roche), PhosSTOP™ (4906845001, Roche), and 0.5mM DTT) and washing was done at 4°C.

For validation of known and putative mono-ADP-ribose interactors with immunoblotting, reactions were scaled down to a final volume of 350µl. Each sample contained 1.5mg of whole cell protein together with 3µM of probe. All subsequent steps followed the workflow described above for the comparison with the mono-ADP-ribose probe. After removal of residual supernatant with 30G syringes, samples were processed for western blotting as detailed in the “Immunoblotting” section.

On-bead digestion

On-bead digestion was performed as previously described.[7] In short, remaining supernatant after PBS washing was removed using 30G syringes, followed by elution (2M urea, 50mM Tris pH 8.5, 10mM DTT) and incubated for 20 minutes in a Thermomixer at room temperature at 1250 rpm. Iodacetamide (Sigma-aldrich) was added to a final concentration of 55mM and incubated in the dark for 10 minutes, 1250 rpm. 250ng of trypsin was added to each sample, incubated for 2 hours, again in a Thermomixer at 1250 rpm. Samples were centrifuged for 2 minutes at 2000 rpm and the supernatant was collected. Beads were washed once more with elution buffer (10 minutes in Thermomixer, then centrifugation). The eluate was collected and added to the previous eluate. An additional 200ng of trypsin was added to each sample for overnight digestion at room temperature. The following day samples were acidified (pH < 2) with 10% v/v trifluoroacetic acid and peptide desalting using StageTips.

For the filter plate samples, the same steps were followed aside from the removal of remaining liquid with 30G syringes, and incubation was performed on a plate shaker at 450rpm. To ensure no supernatant was lost, the filter plate was added on top of a flat-bottom 96-wells plate immediately, after which the elution buffer was added to each well. To collect the supernatant, the filter plate and collection plate together were centrifuged for 5min at 500g.

Label-free quantification by LC-MS/MS

C18 5µm trap column (300µm x 5mm, Thermo Scientific), before separation on the analytical column (AUR325075b18TS, 1.7µm/75µm x 250mm, Thermo Scientific) mounted into an Easyspray ion source (Thermo Scientific) with 1500V spray voltage applied. The column was heated at 50°C and the flow rate was set to 0.5µL/min at the start of the method to minimize delay time. Solvent A was 0.1% formic acid/water and solvent B was 0.1% formic acid/80% acetonitrile. Peptides were eluted at a flow rate of 0.4 µL/min in a 36-min effective gradient, containing a non-linear increase from 1% to 45% solvent B and a 0.4-min ramp to 99% solvent B at 0.5 µL/min flow rate at the end. The column was washed for 3.9 min. at 0.5 µL/min at 99%B and finally equilibrated using the "fast equilibration" script in combined control mode with a 1500 bar pressure limit. The Orbitrap Astral was run in data-independent acquisition (DIA) mode, with full MS scans being collected in the Orbitrap analyzer with 240,000 resolution at m/z 200 over a 380-980 m/z range. Default charge state was 2+, the normalized AGC

target was set to 500% (equivalent to 5e6 charges) and the maximum injection time was set to 5 ms. For DIA MS2, a normalized HCD collision energy of 25% was applied to a 380-980 m/z precursor range using non-overlapping isolation windows of 2Th, with window placement optimization turned on. Scans were acquired in the Astral analyzer over a 100-1000 m/z range, with the normalized AGC target set to 500% (equivalent to 5e4 charges) with a maximum injection time of 3 ms. The mass spectrometry proteomics data have been deposited to the ProteomeXchange Consortium via the PRIDE[8] partner repository with the dataset identifier PXD065574.

Mass spectrometry data analysis

Raw mass spectrometry spectra were processed using DIA-NN software (version 1.9.2) according to the developers guidelines.[9] For figure 2, files were processes in Perseus; MaxLFQ intensity values from DIA-NN were log_2 transformed, followed by filtering to detect proteins present in all replicates of at least one experimental triplicate (version 1.6.15.0).[10] Missing values in the dataset were imputed following a normal distribution (shift = 1.8 and width. = 0.3) with the assumption that these proteins were just below the detection limit. Final data processing and visualization was done in R. Gene set enrichment analysis (GSEA) was conducted using the fgsea R package, with genes ranked by log_2 fold change. Only proteins that were identified as statistically significant were included in the analysis. Gene sets were sourced from the MSigDB Gene Ontology Biological Process collection. Pathways with adjusted p-values below 0.05 were considered significantly enriched. Only the top 10 biological processes (based on normalized enrichment score) were included for final data visualization.

Recombinant protein purification

PARP9 MD1 with His-MBP tag was expressed in *Escherichia coli* BL21 (DE3) cells after induction with 1mM IPTG for 16h at 18°C. Cells were harvested by centrifugation at 4000g for 15min. After harvesting, cells were resuspended in 20mM Tris-HCl, 200mM NaCl pH 8.0 buffer supplemented with 5µg/ml DNase and cOmplete, EDTA-free protease inhibitor cocktail (Sigma-Aldrich) and lysed by sonication. The purification was done by affinity chromatography using Chelating Sepharose Fast Flow (Cytiva) charged with Ni^{2+}. The washing steps included washing with 20mM Tris-HCl, 1.5M NaCl pH 8.0 buffer and 20mM Tris-HCl, 200mM NaCl and 20mM imidazole pH 8.0 buffer. Protein was then eluted with 20mM Tris-HCl, 200mM NaCl and 300mM imidazole pH 8.0 buffer. His-MBP tag was not removed to allow for visualization with the anti-His-tag antibody. The vector for PARP9 MD1 production was provided by the Georgios Spyroulias lab (University of Petras).

Validation of direct interaction with recombinant proteins

For validation experiments, recombinant CTBP1 (Novus Biologicals, # NBP1-41161) and recombinant PARP9 macrodomain 1 (see "Recombinant protein purification") were diluted in PBS to a final amount of per reaction. Each reaction (18µl total volume) contained 3µM of either the mono-ADPr photoaffinity probe (compound 1a) or the corresponding control probe (compound 2a). Samples were kept on ice for 10min, followed by 30min UV irradiation in an RMR-600 photochemical reactor (Rayonet). PCR tubes were laid flat with their lids open and positioned approximately 1cm from the lamps. After irradiation, samples were processed for immunoblotting (see following section).

Immunoblotting

Protein separation was done with SDS-PAGE by using a 4-15% TGX gradient gel (Bio-rad). Proteins were transferred via dry transfer (iBlot 3.0, Invitrogen) to a 0.22µm nitrocellulose membrane (iBlot 3.0 transfer stacks, Invitrogen) using the low range setting. Membranes were blocked with 5% non-fat dried milk in PBS-T and incubated for 2 hours at room temperature with the primary antibody: CTBP1 polyclonal antibody (Proteintech #10972-1-AP) or THE™ His Tag Antibody (GenScript, #A00186-100). Following this, membranes were washed three times with PBS-T and incubated with the streptavidin antibody (IRDye 680RD Streptavidin, Licor, #926-

68079, or IRDye 800CW Streptavidin, Licor, # 926-32230) and the secondary antibody (IRDye 800CW Donkey anti-Rabbit, Licor, #925-32213, or IRDye 680RD Goat anti-Mouse, Licor, #926-68180) for 45 minutes at room temperature. Membranes were finally washed three times with PBS-T and imaged using an Odyssey Clx Imager.

3. Characterization Data

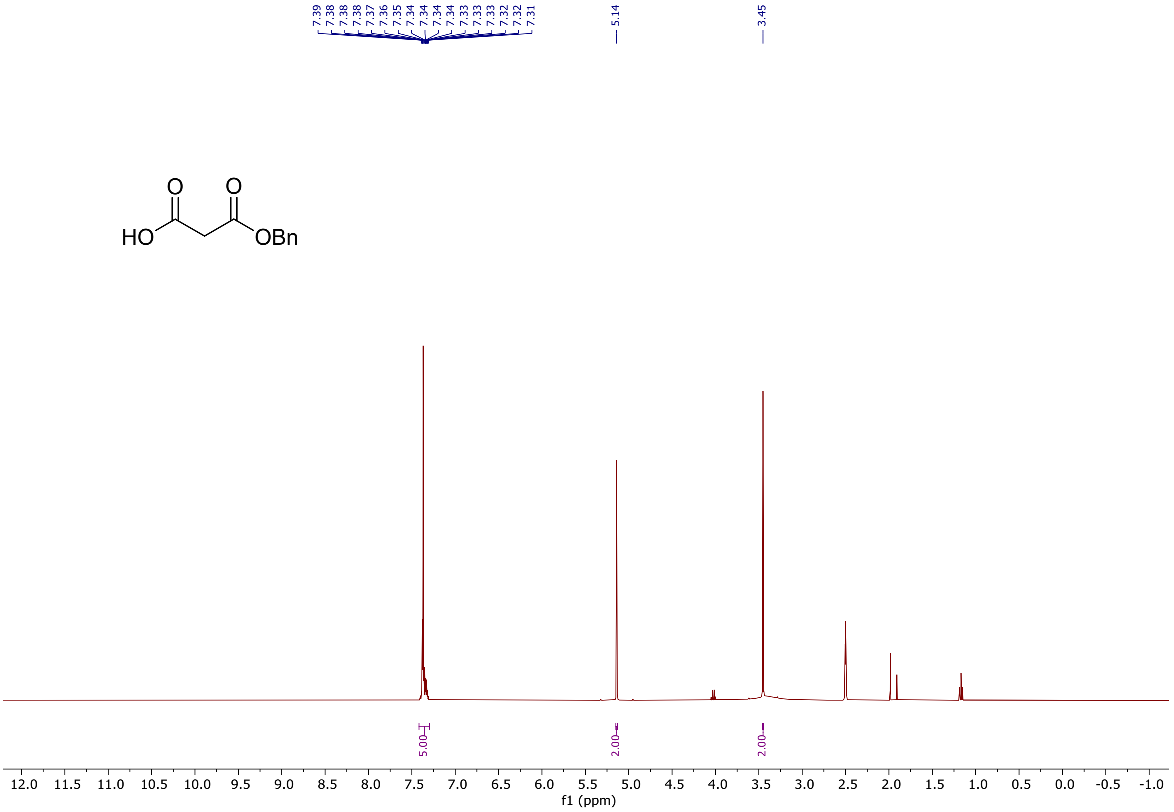

Figure S4: ^{1}H NMR Spectrum of Compound **S1**

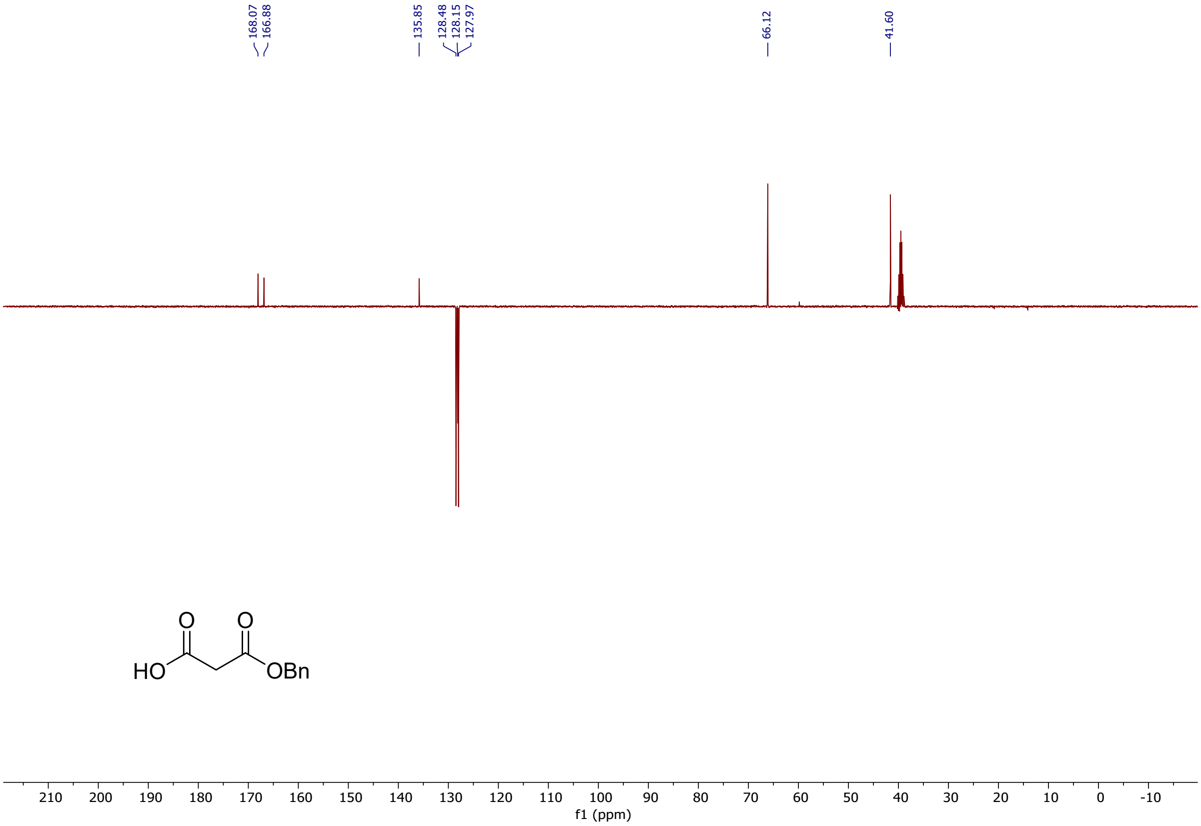

Figure S5: ^{13}C NMR Spectrum of Compound **S1**

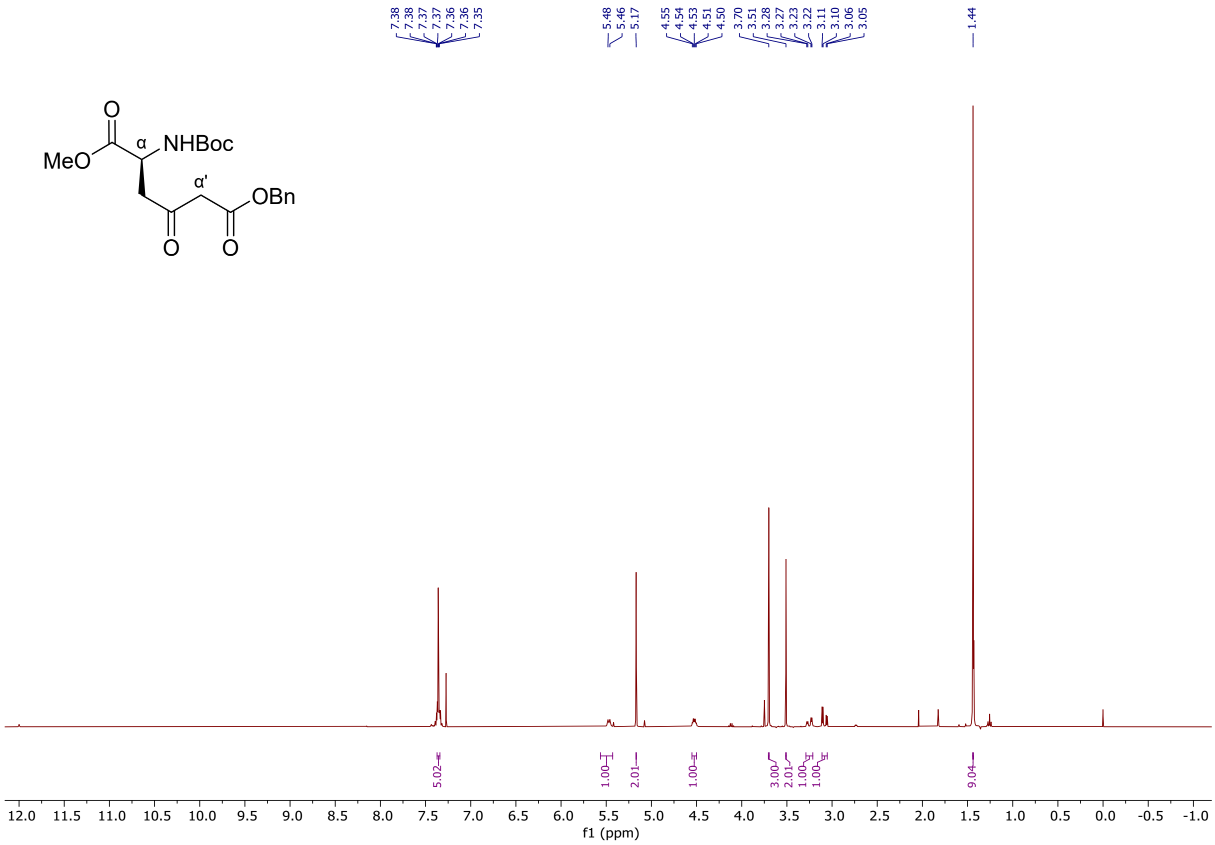

Figure S6: ^{1}H NMR Spectrum of Compound **S2**

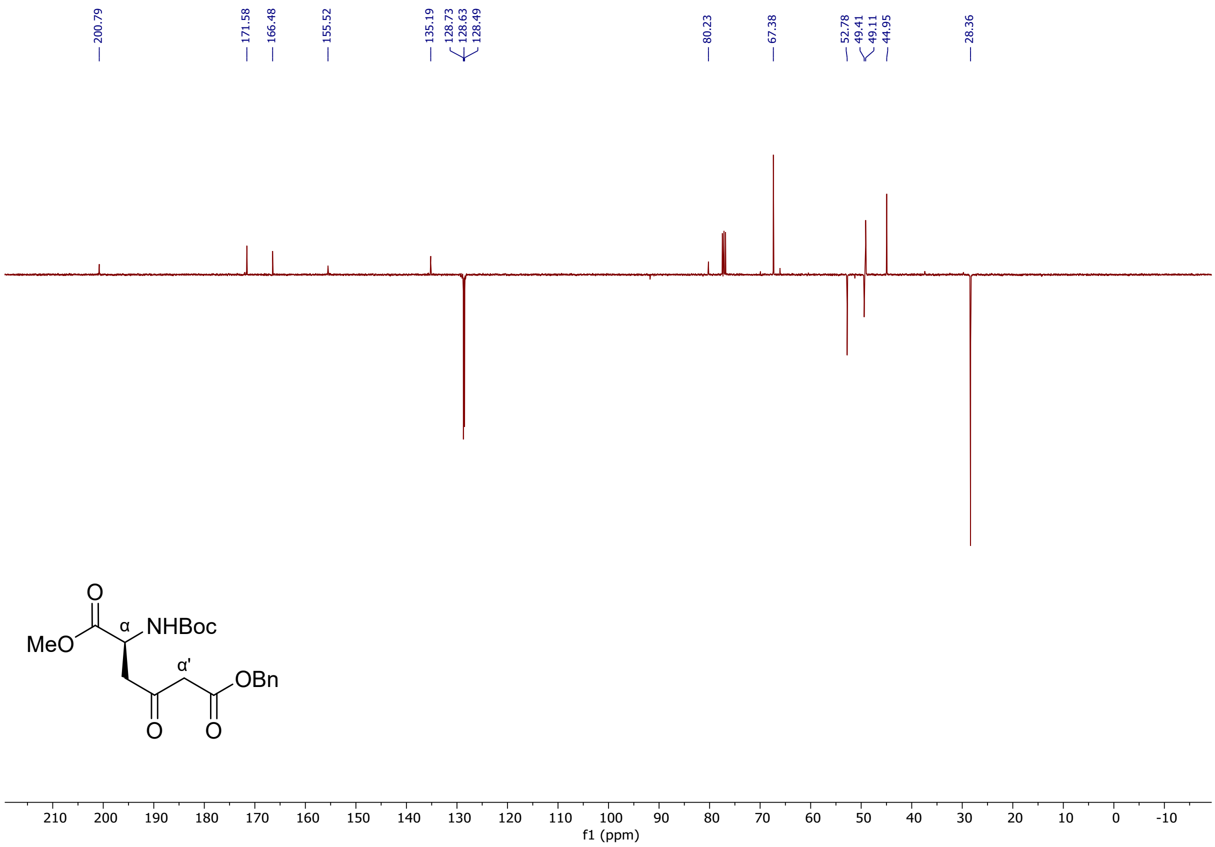

Figure S7: ^{13}C NMR Spectrum of Compound **S2**

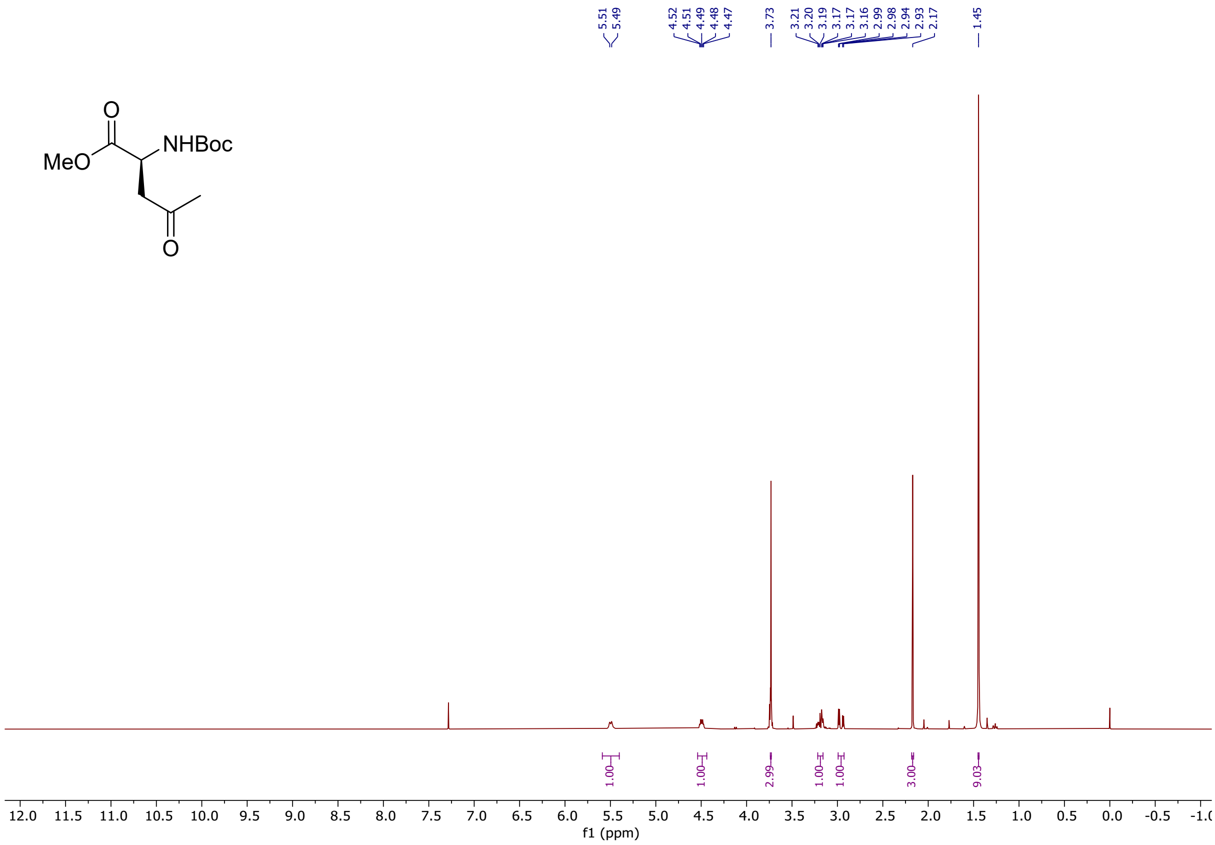

Figure S8: ^{1}H NMR Spectrum of Compound **S3**

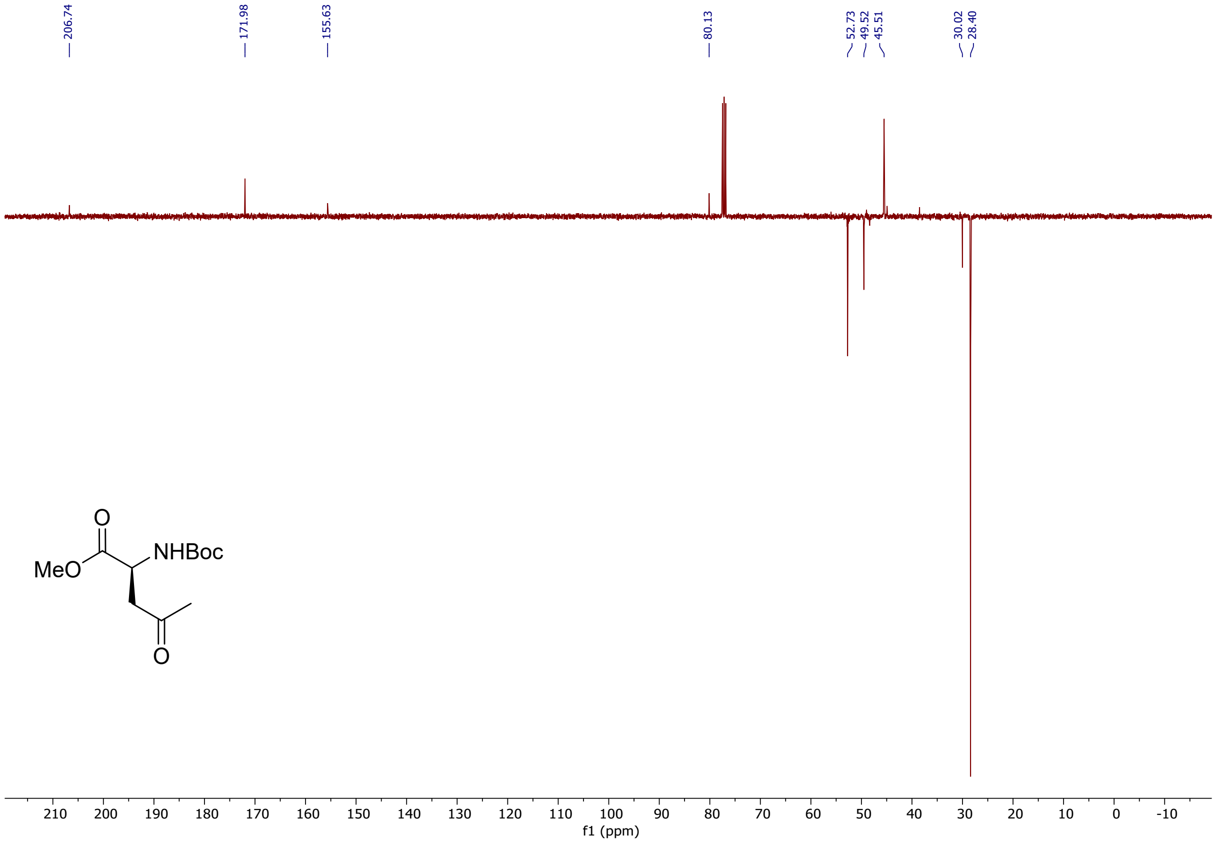

Figure S9: ^{13}C NMR Spectrum of Compound **S3**

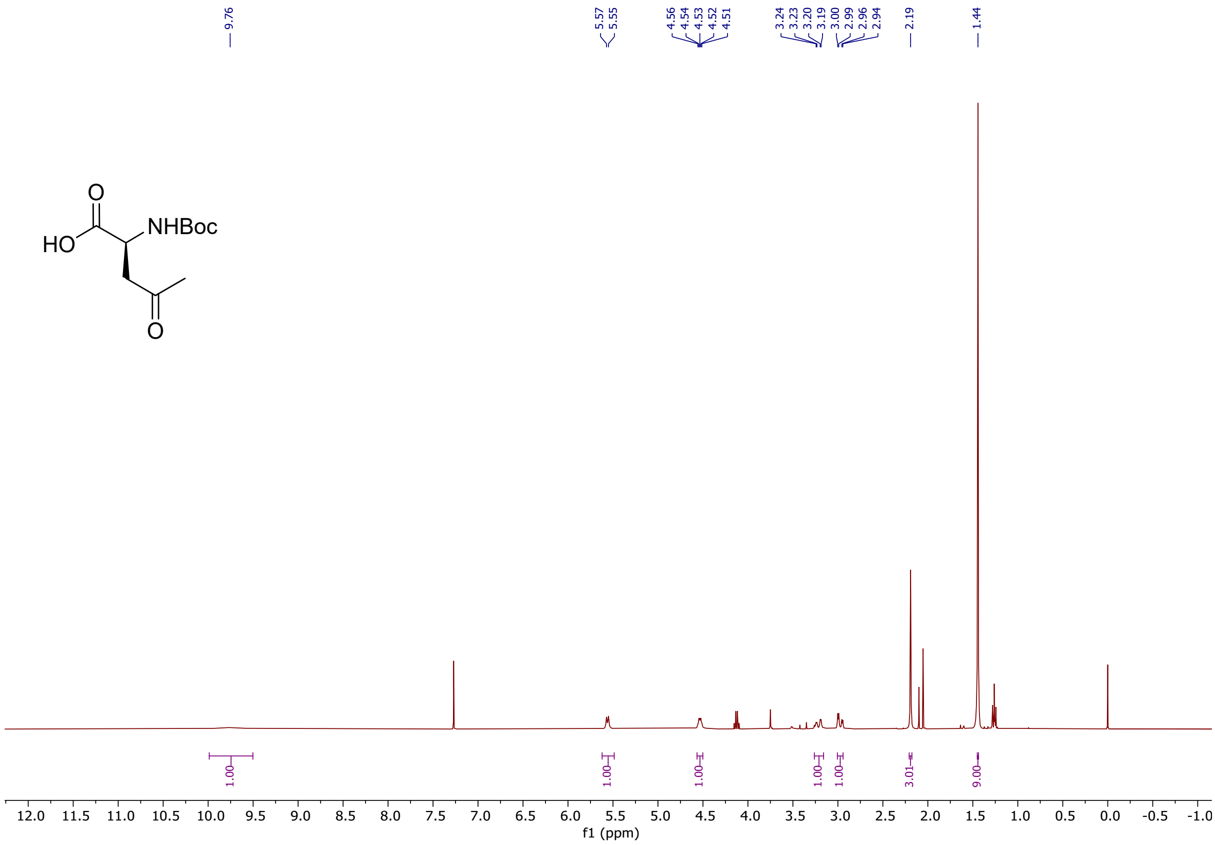

Figure S10: ^{1}H NMR Spectrum of Compound **S4**

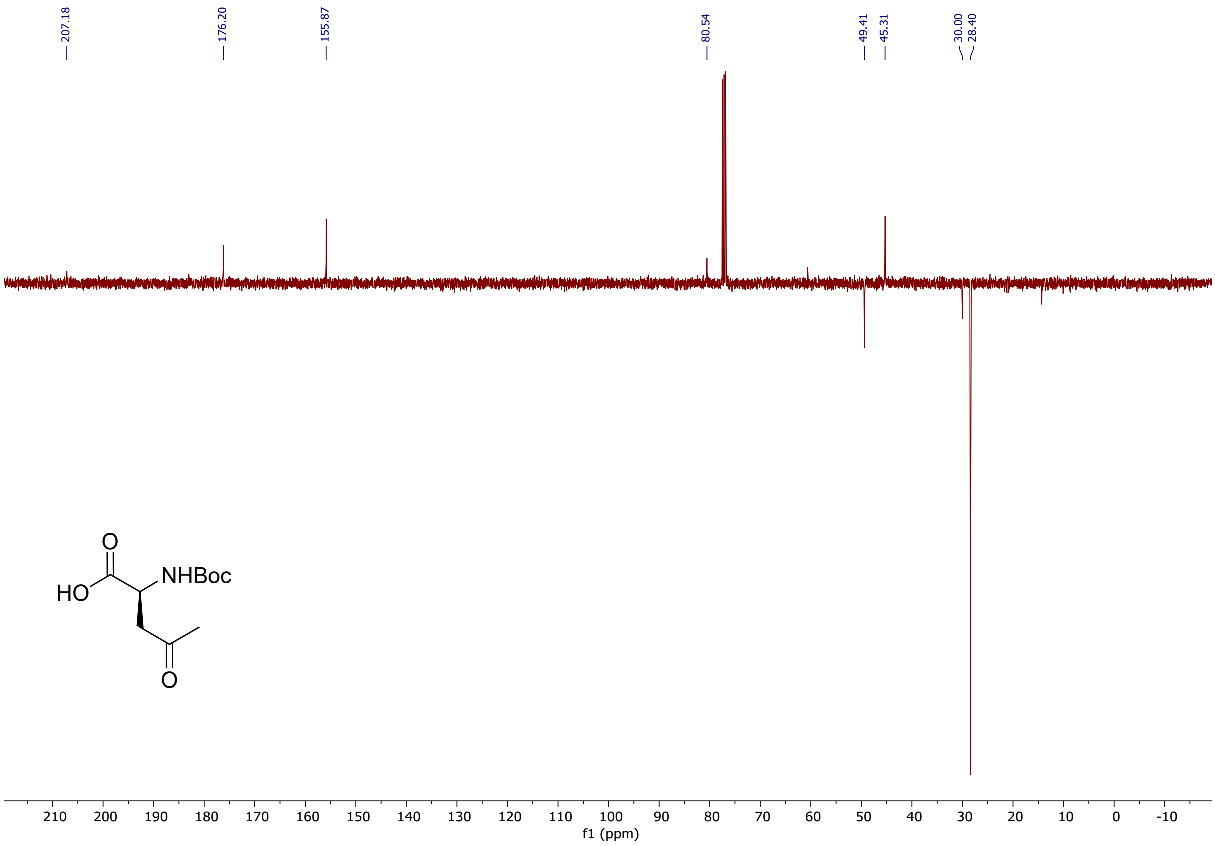

Figure S11: ^{13}C NMR Spectrum of Compound **S4**

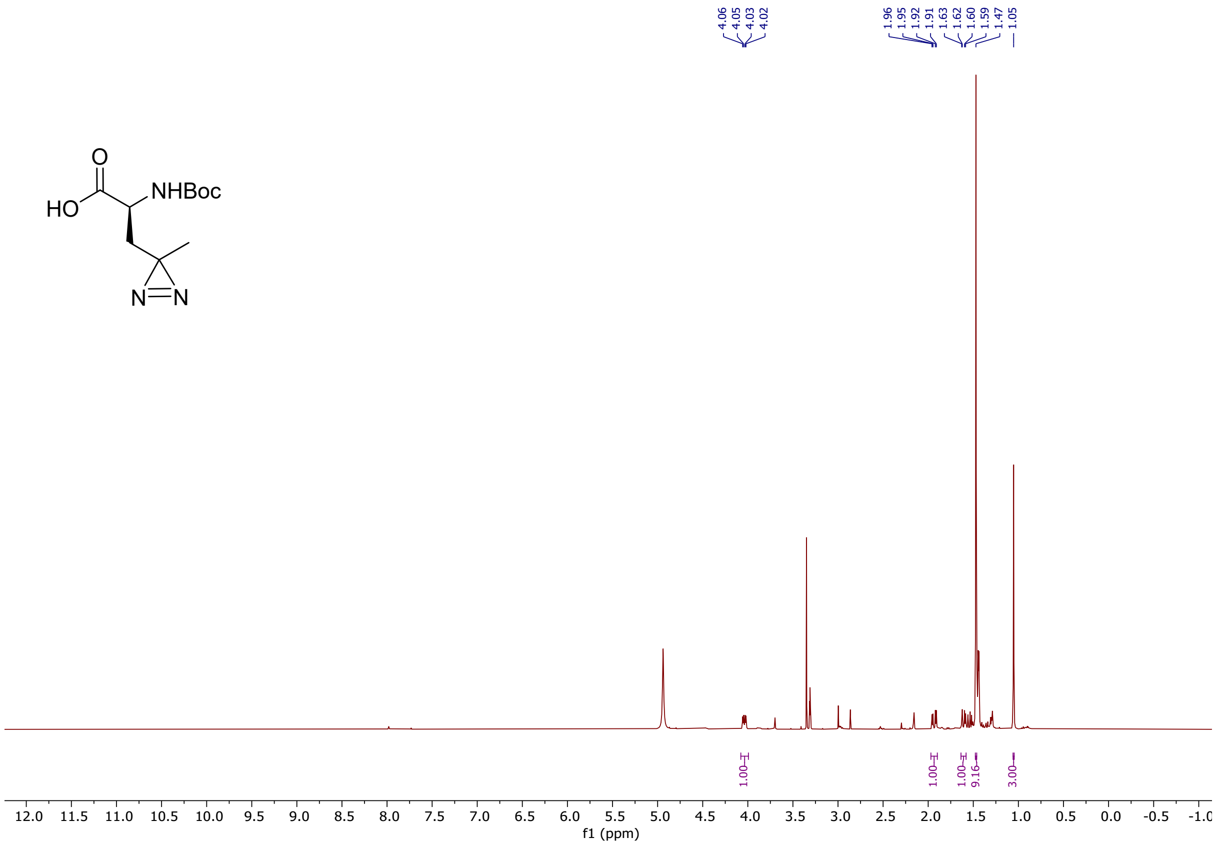

Figure S12: ^{1}H NMR Spectrum of Compound **4a**

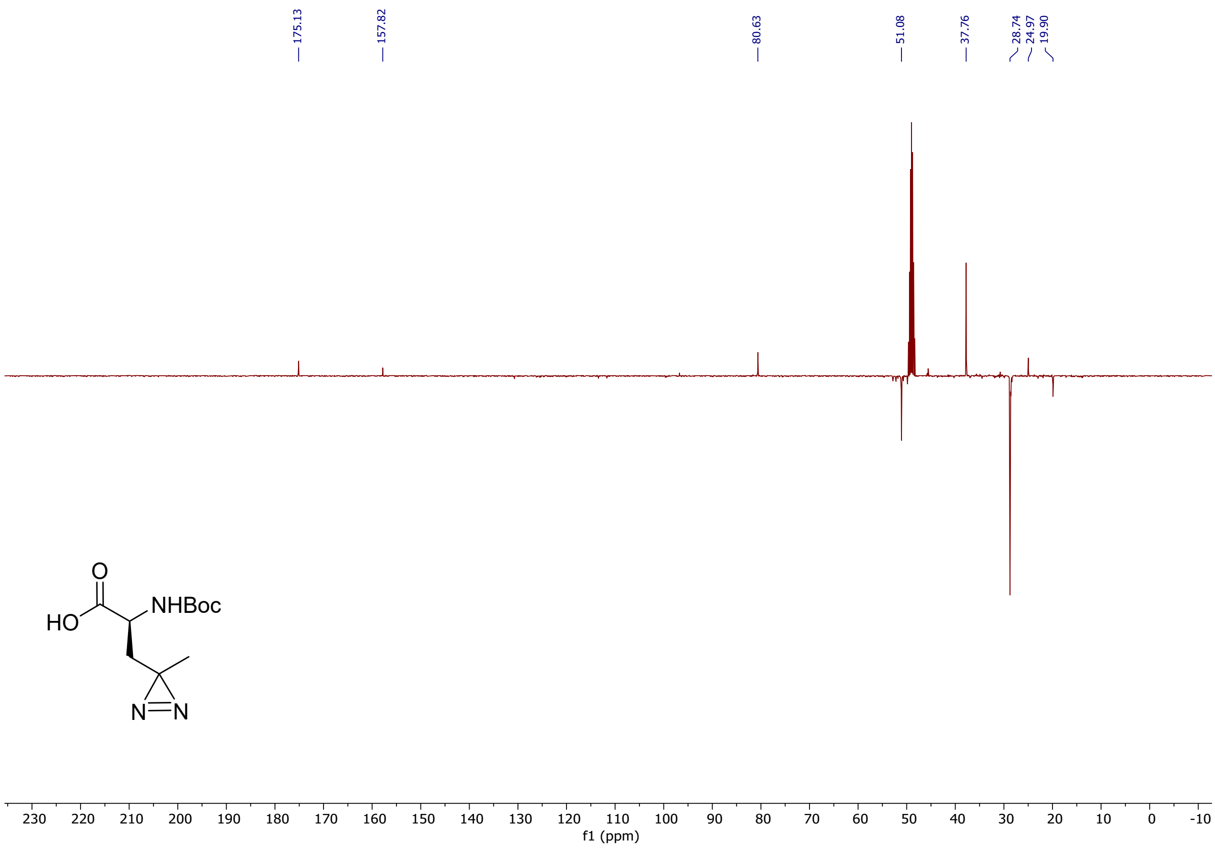

Figure S13: ^{13}C NMR Spectrum of Compound **4a**

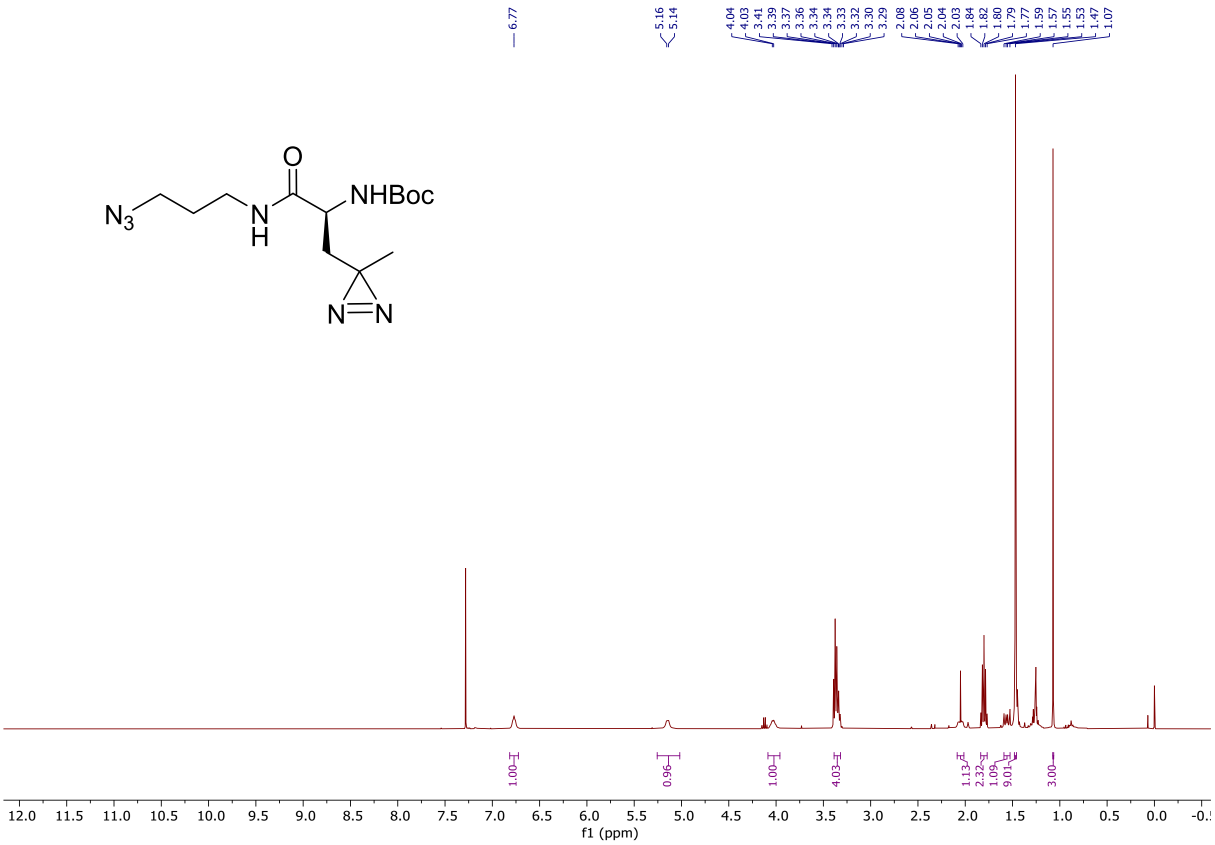

Figure S14: ^{1}H NMR Spectrum of Compound **5a**

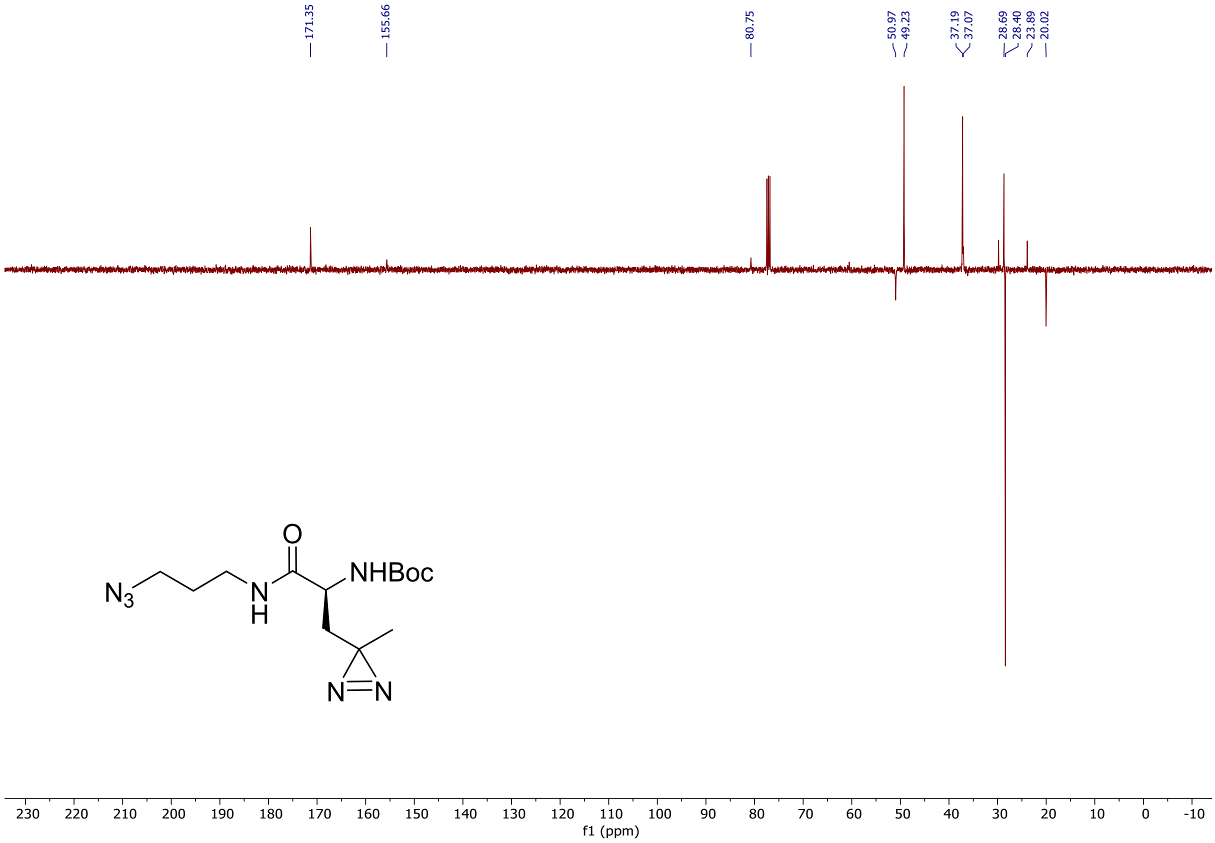

Figure S15: ^{13}C NMR Spectrum of Compound **5a**

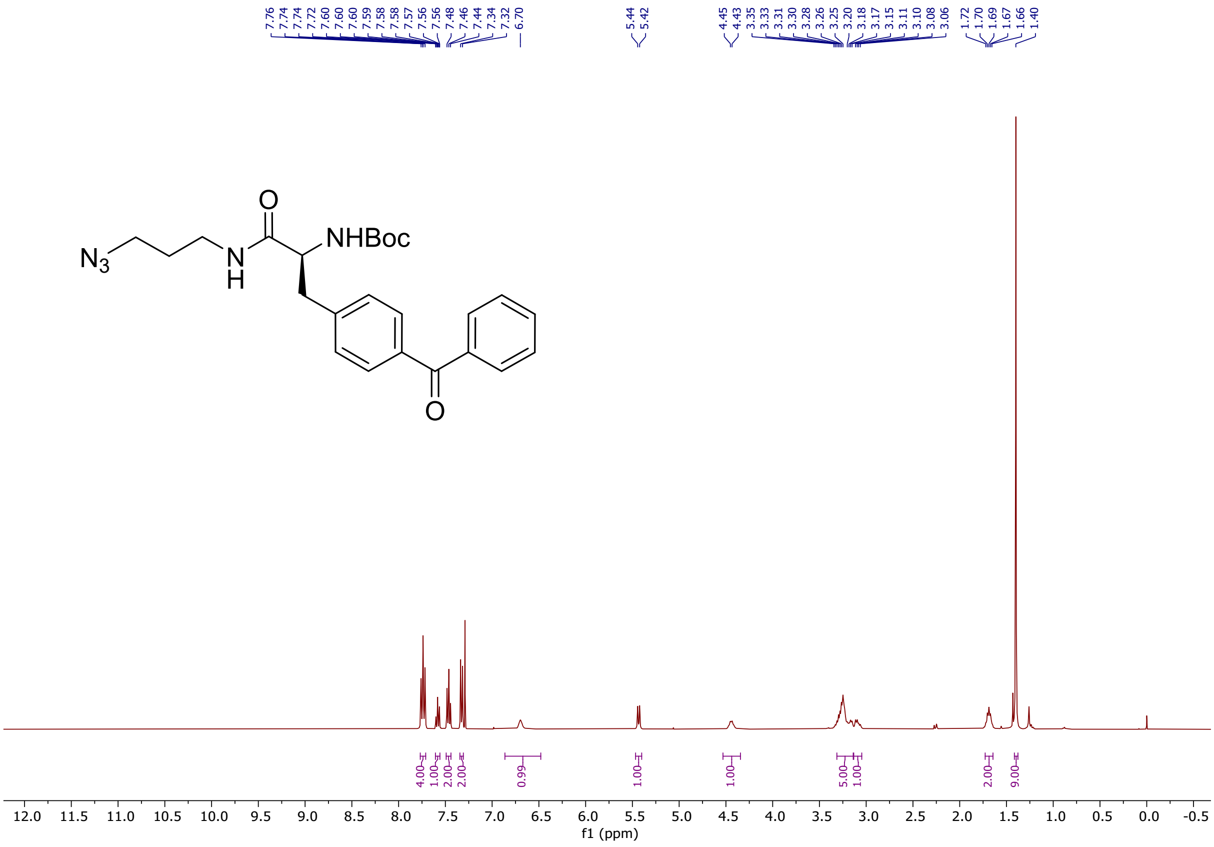

Figure S16: ^{1}H NMR Spectrum of Compound **5b**

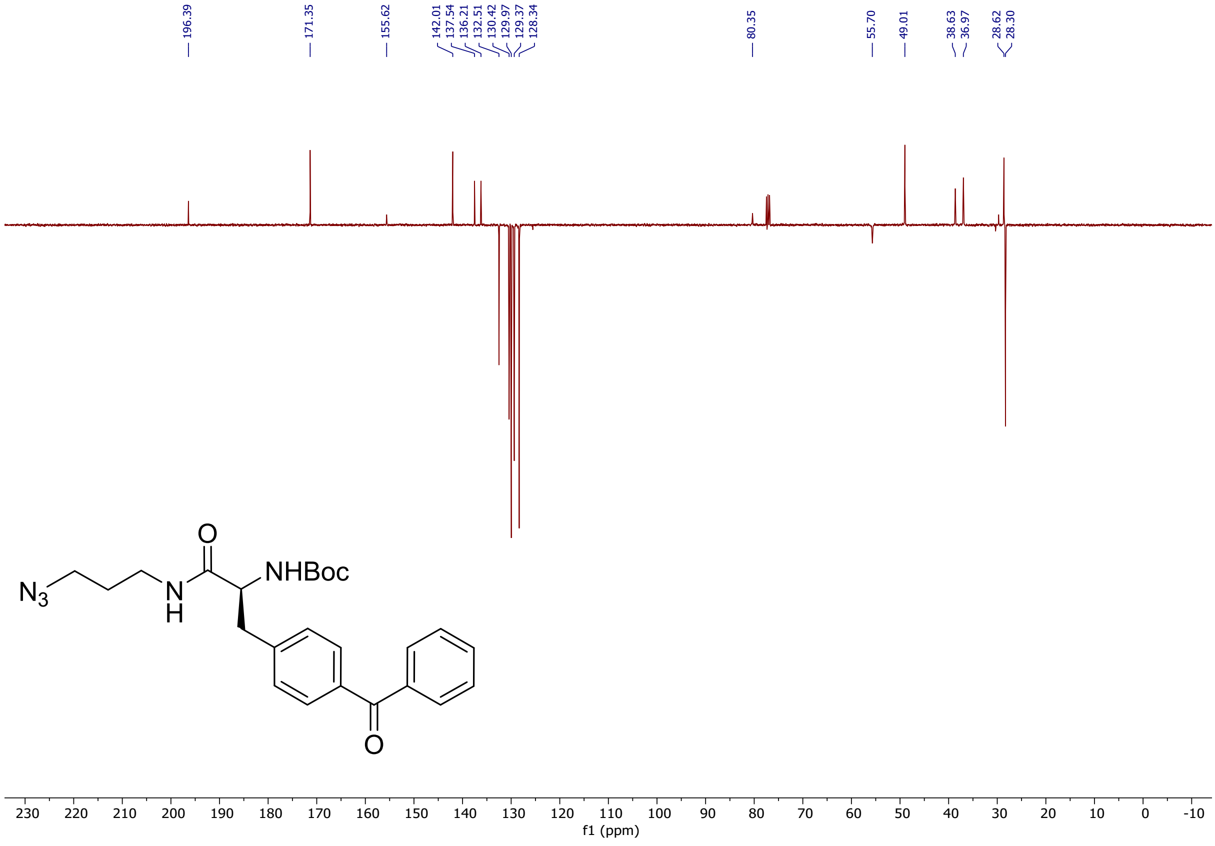

Figure S17: ^{13}C NMR Spectrum of Compound **5b**

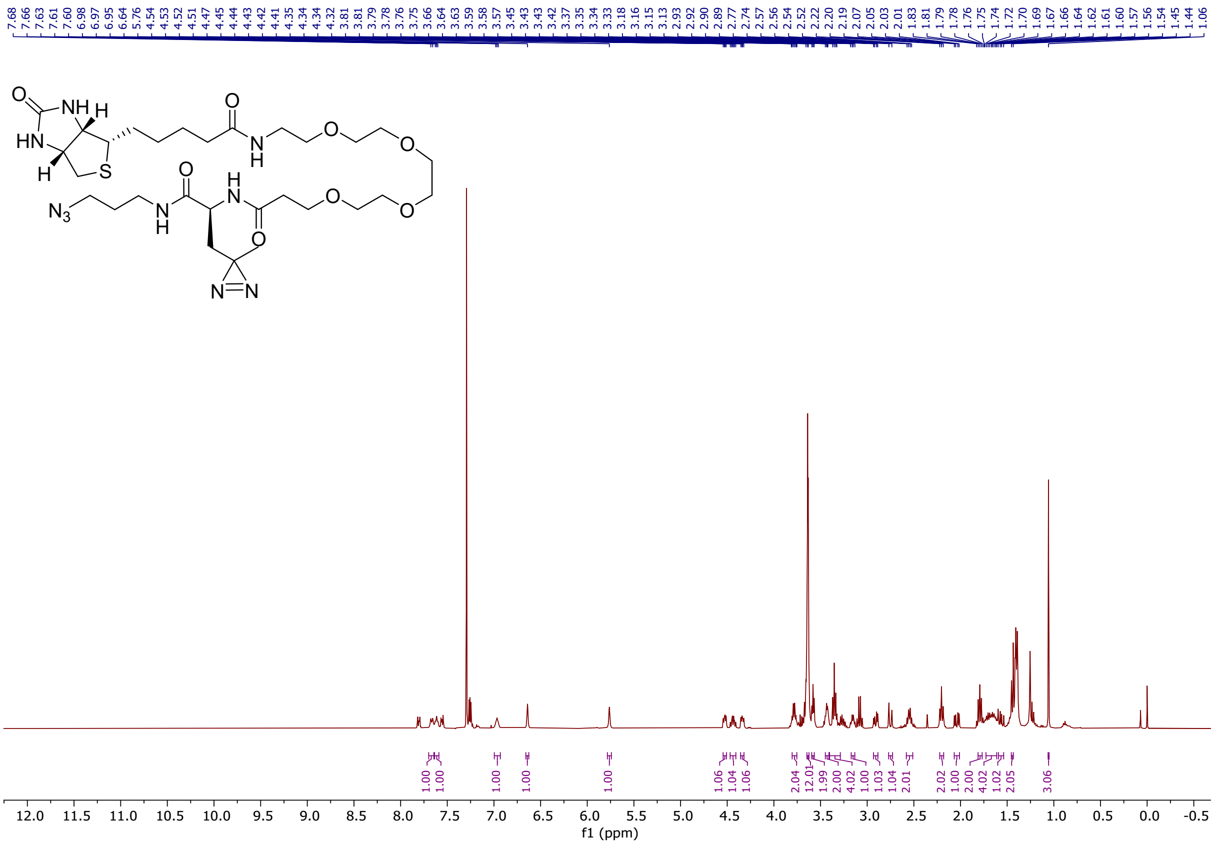

Figure S18: ^{1}H NMR Spectrum of Compound **2a**

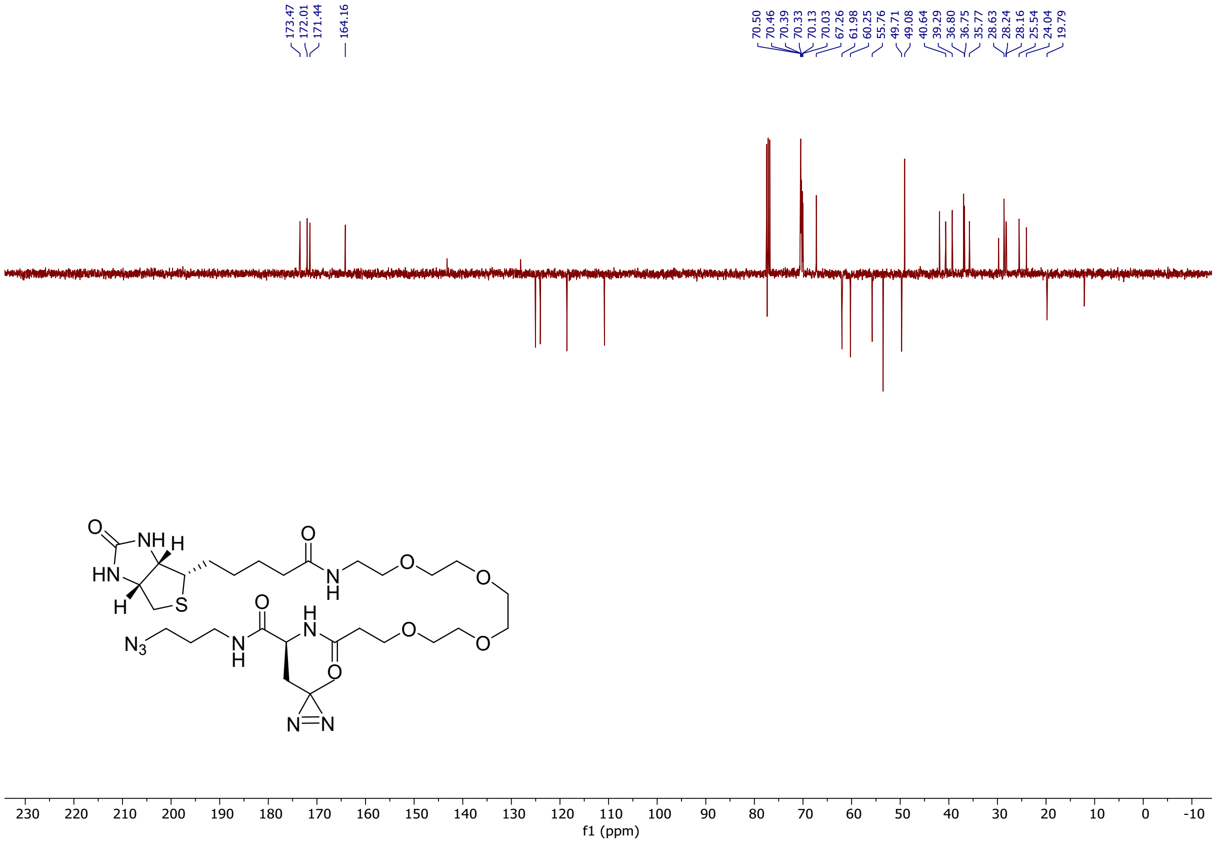

Figure S19: ^{13}C NMR Spectrum of Compound **2a**

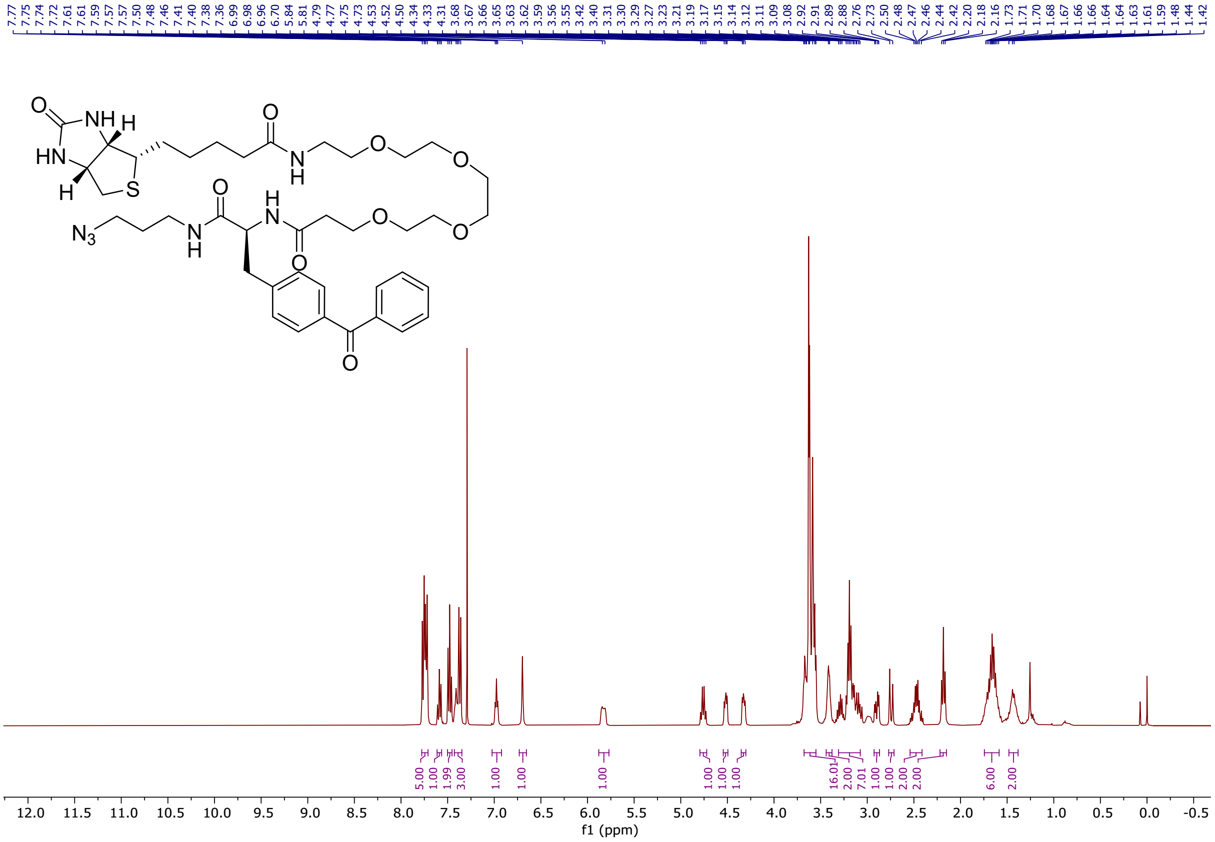

Figure S20: ^{1}H NMR Spectrum of Compound **2b**

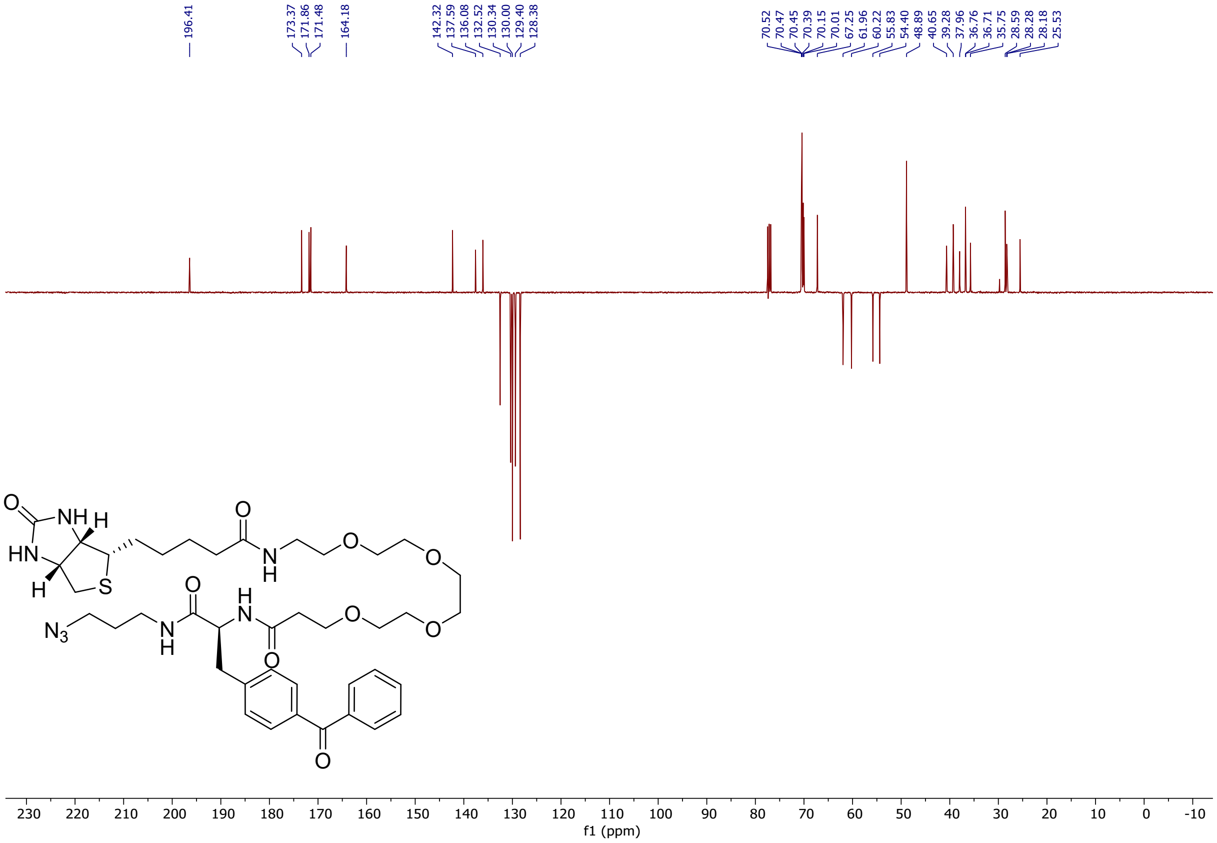

Figure S21: ^{13}C NMR Spectrum of Compound **2b**

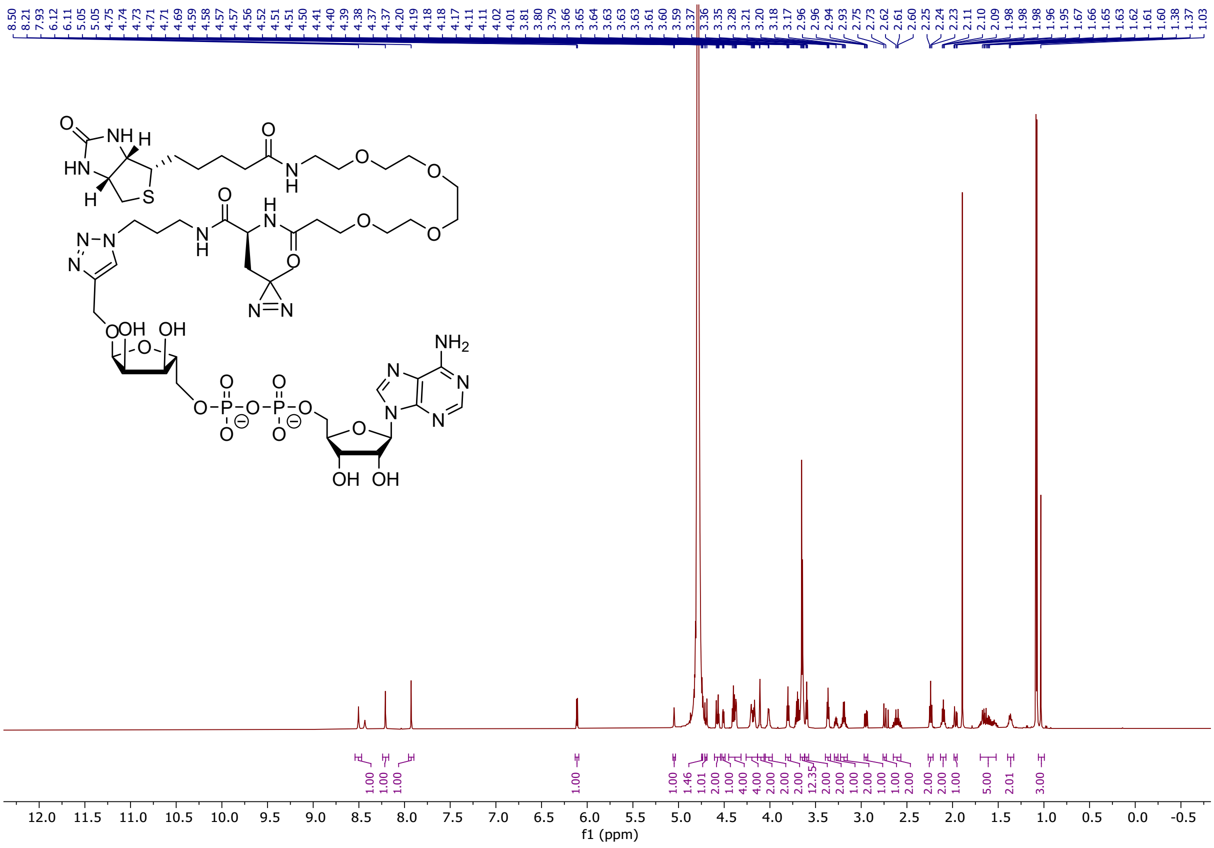

Figure S22: ^{1}H NMR Spectrum of Compound **1a**

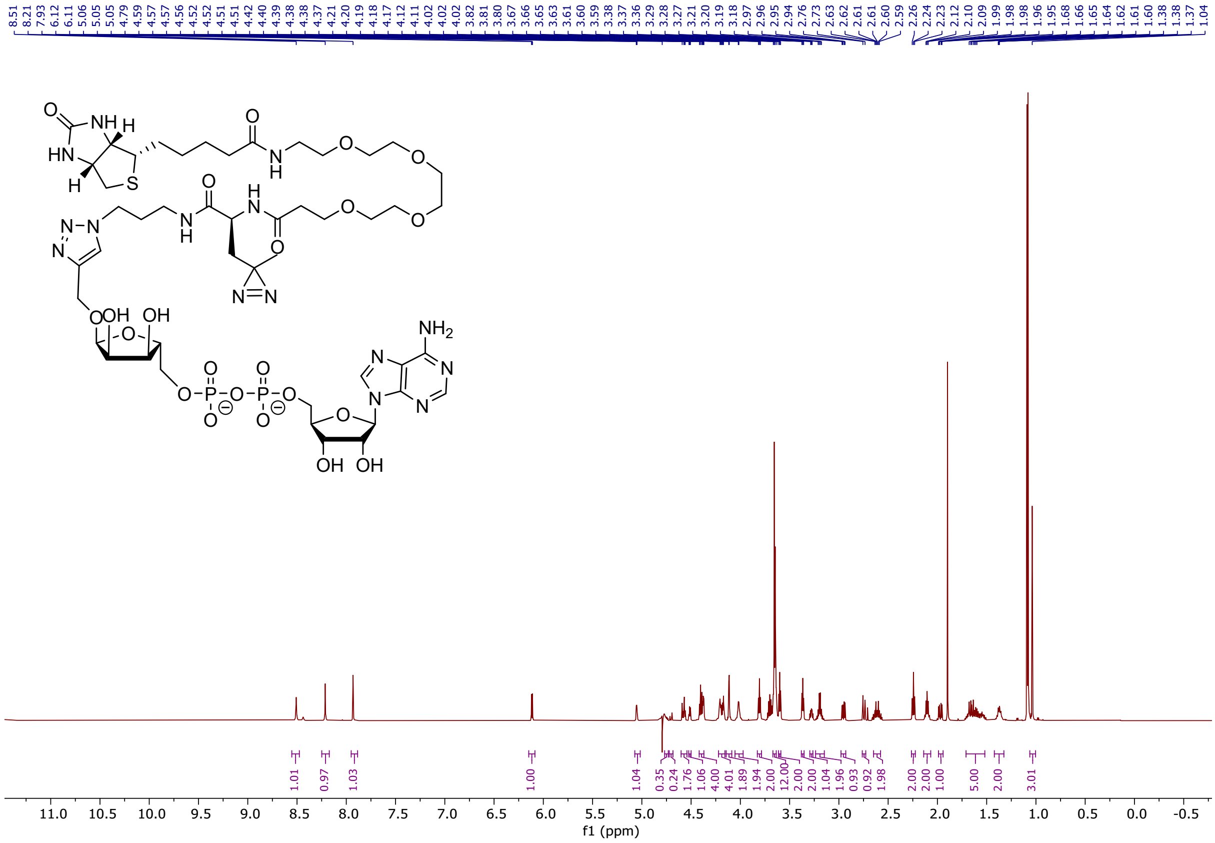

Figure S23: ^{1}H NMR Spectrum (presat) of Compound **1a**

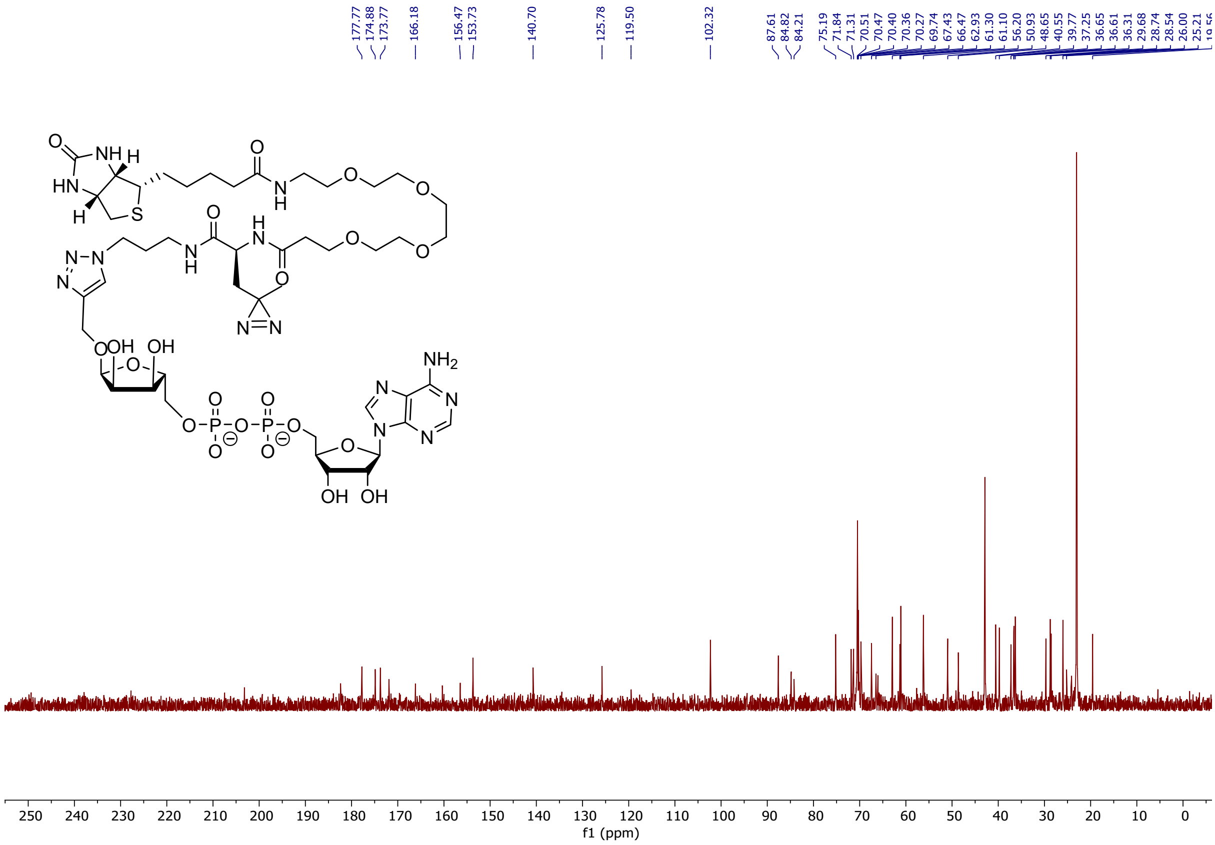

Figure S24: ^{13}C NMR Spectrum of Compound **1a**

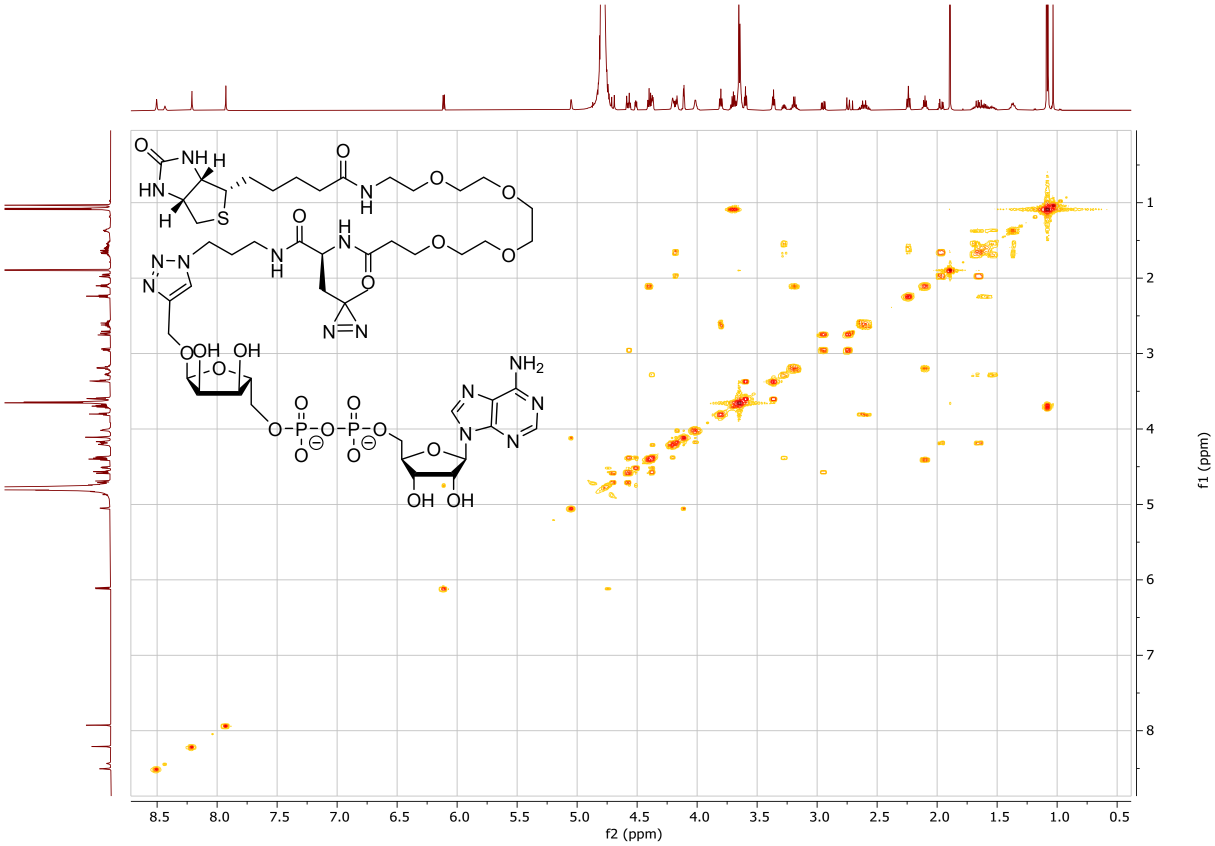

Figure S25: COSY Spectrum of Compound **1a**

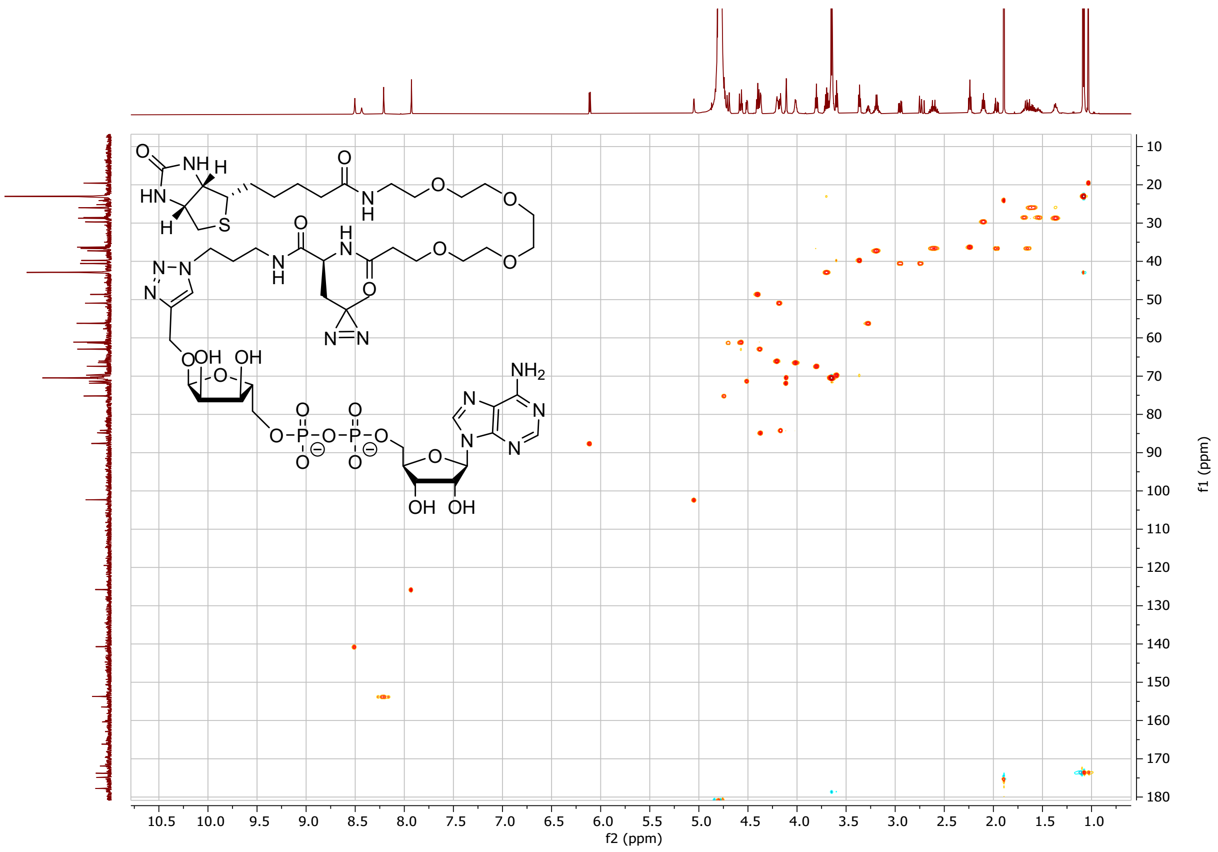

Figure S26: HSQC Spectrum of Compound **1a**

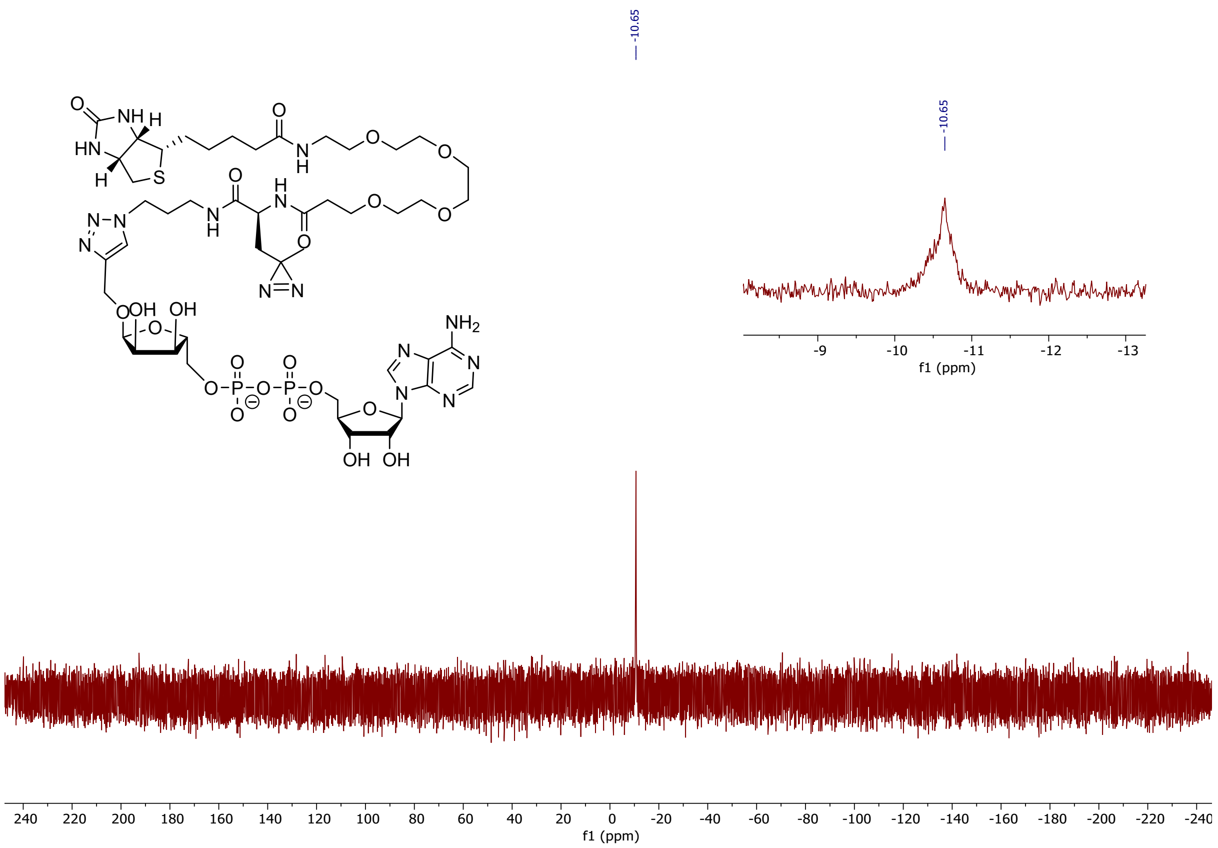

Figure S27: ^{31}P NMR Spectrum of Compound **1a**

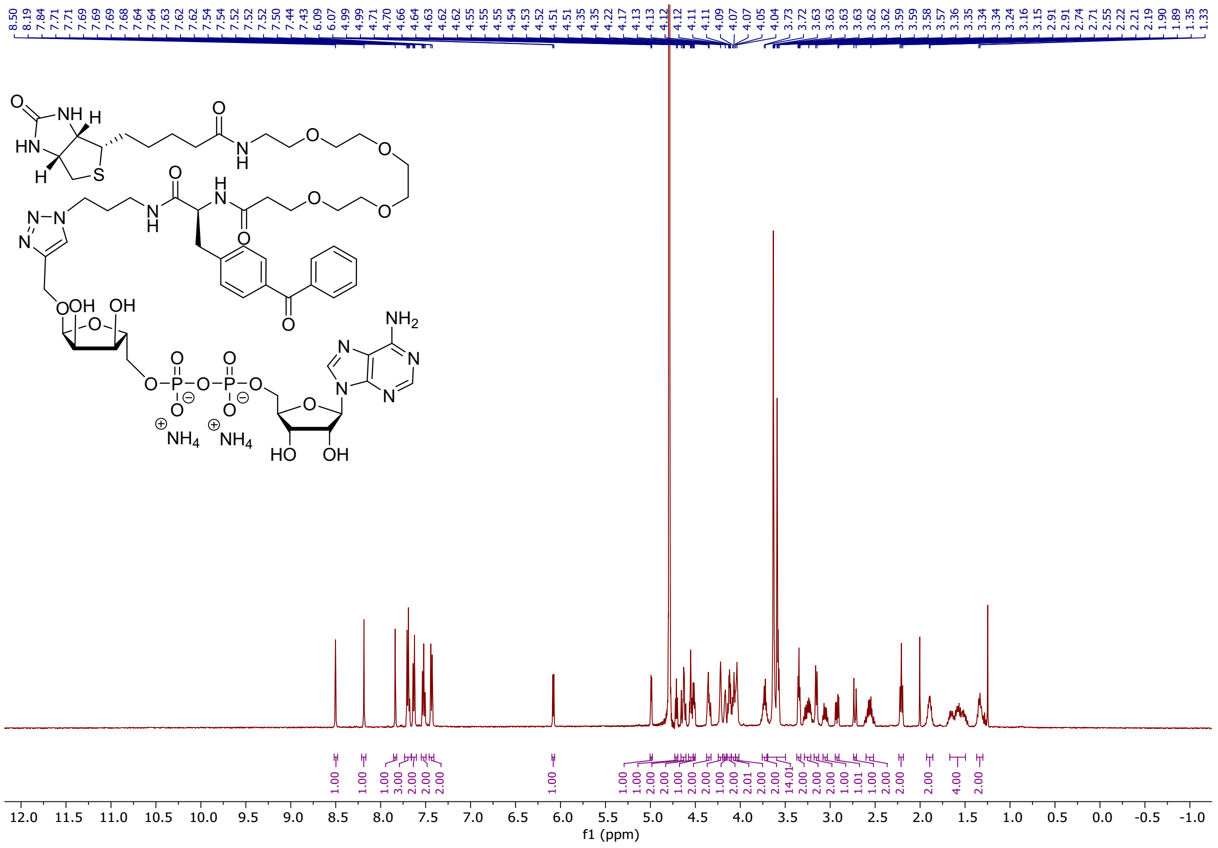

Figure S28: ^{1}H NMR Spectrum of Compound **1b**

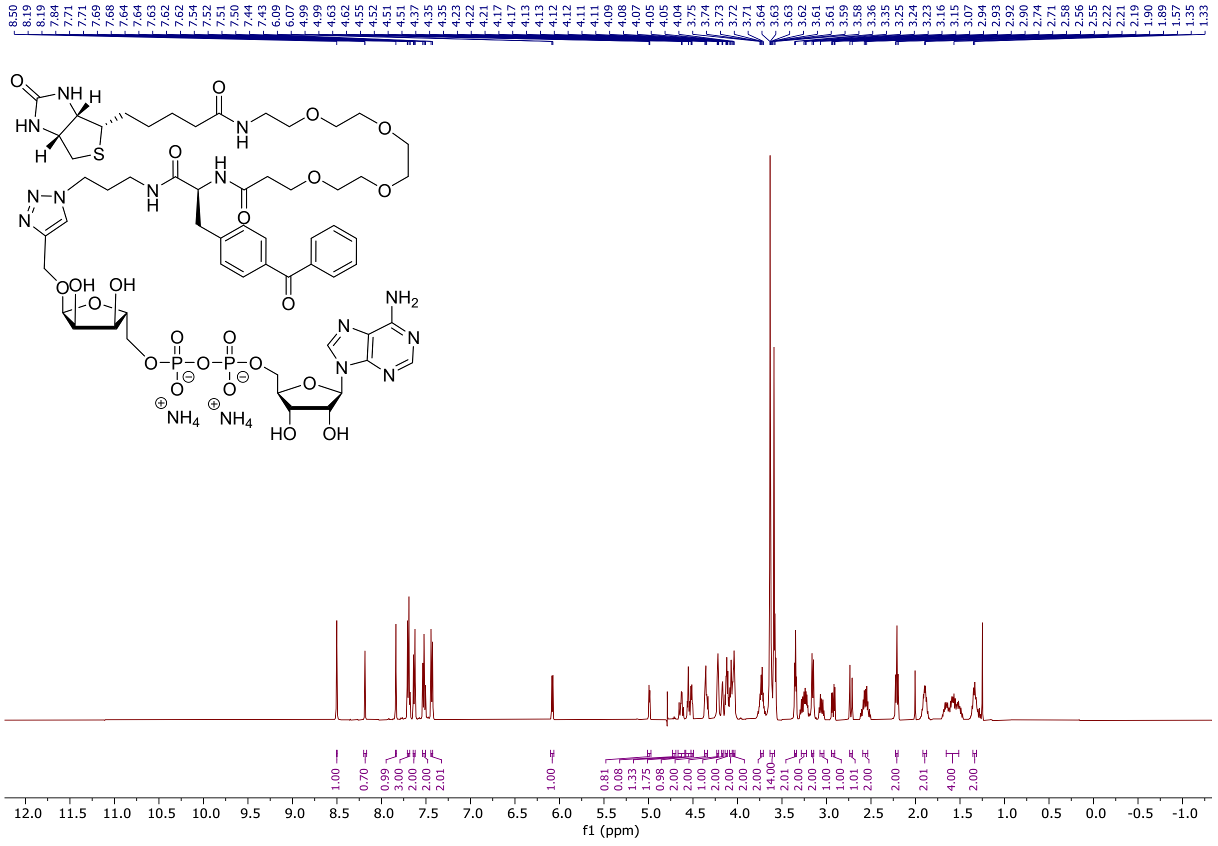

Figure S29: ^{1}H NMR Spectrum (presat) of Compound **1b**

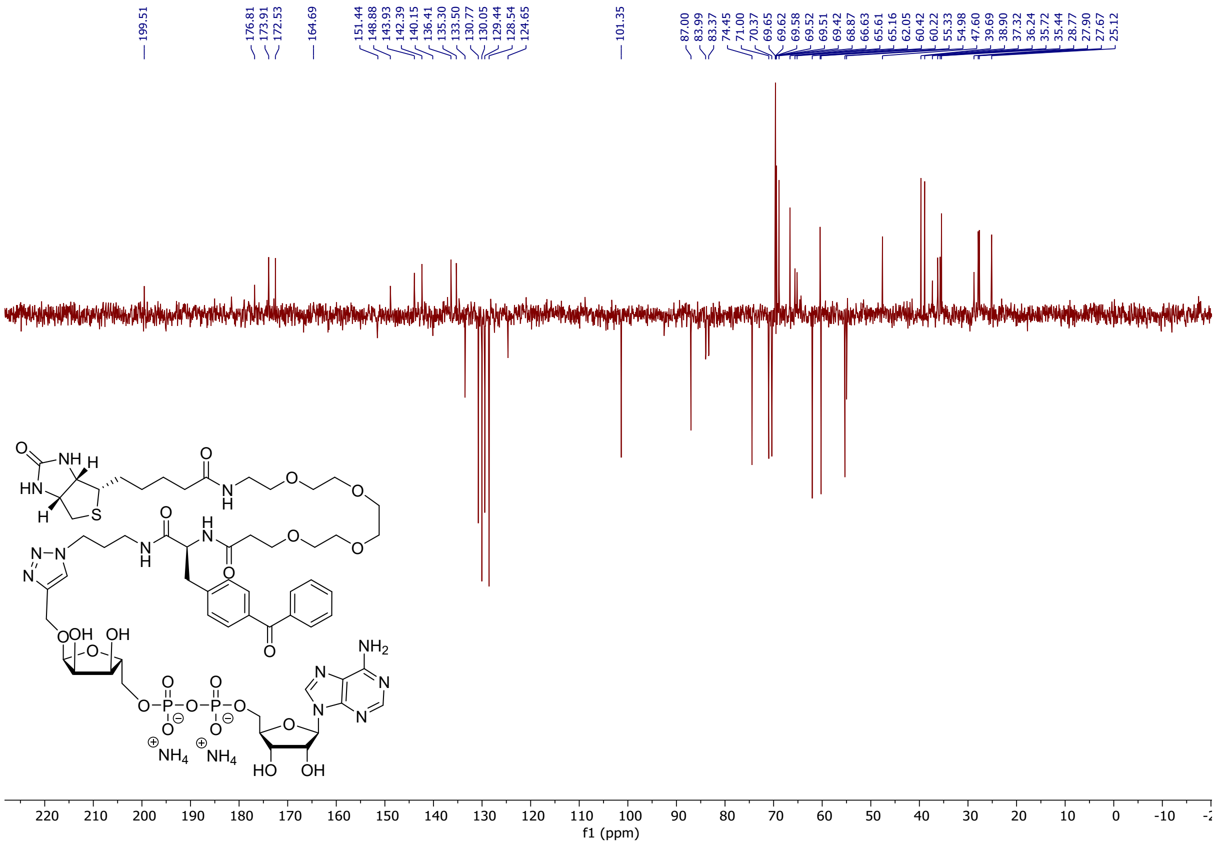

Figure S30: ^{13}C NMR Spectrum of Compound **1b**

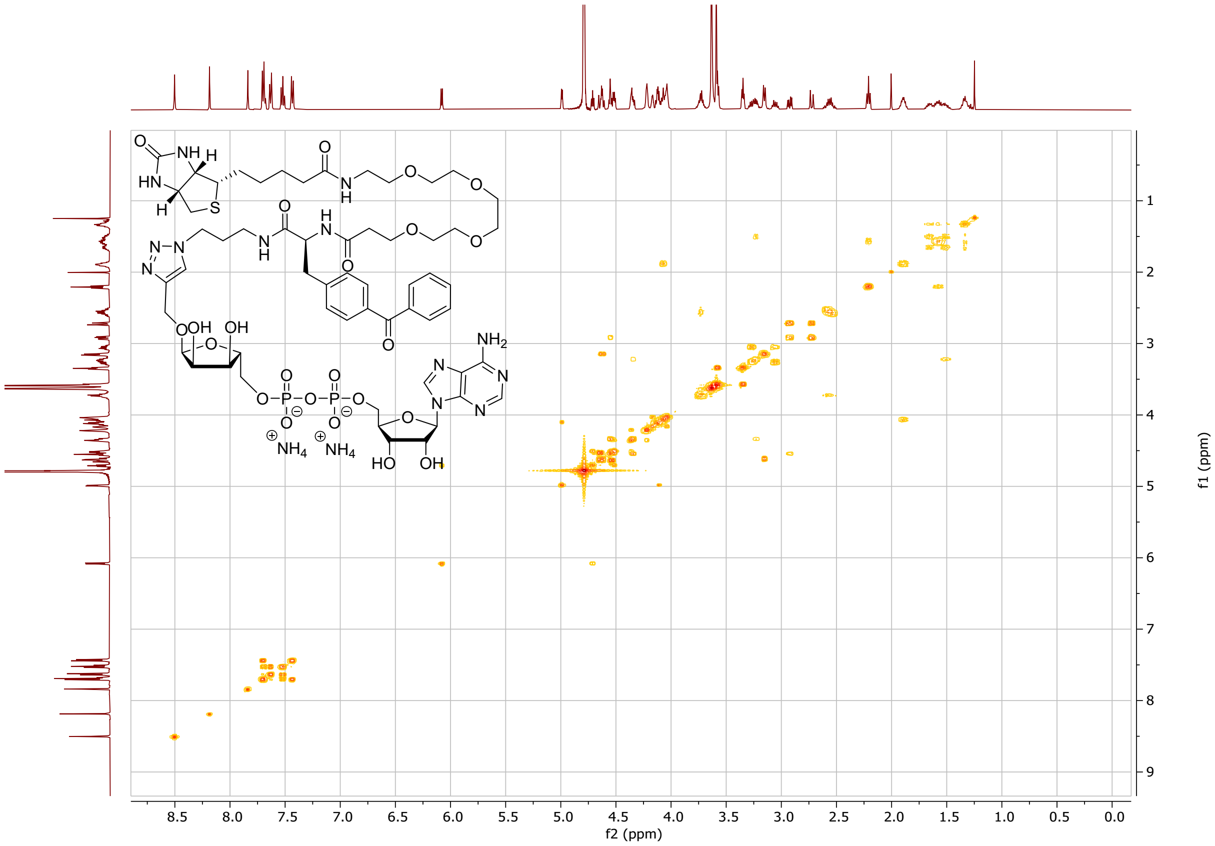

Figure S31: COSY Spectrum of Compound **1b**

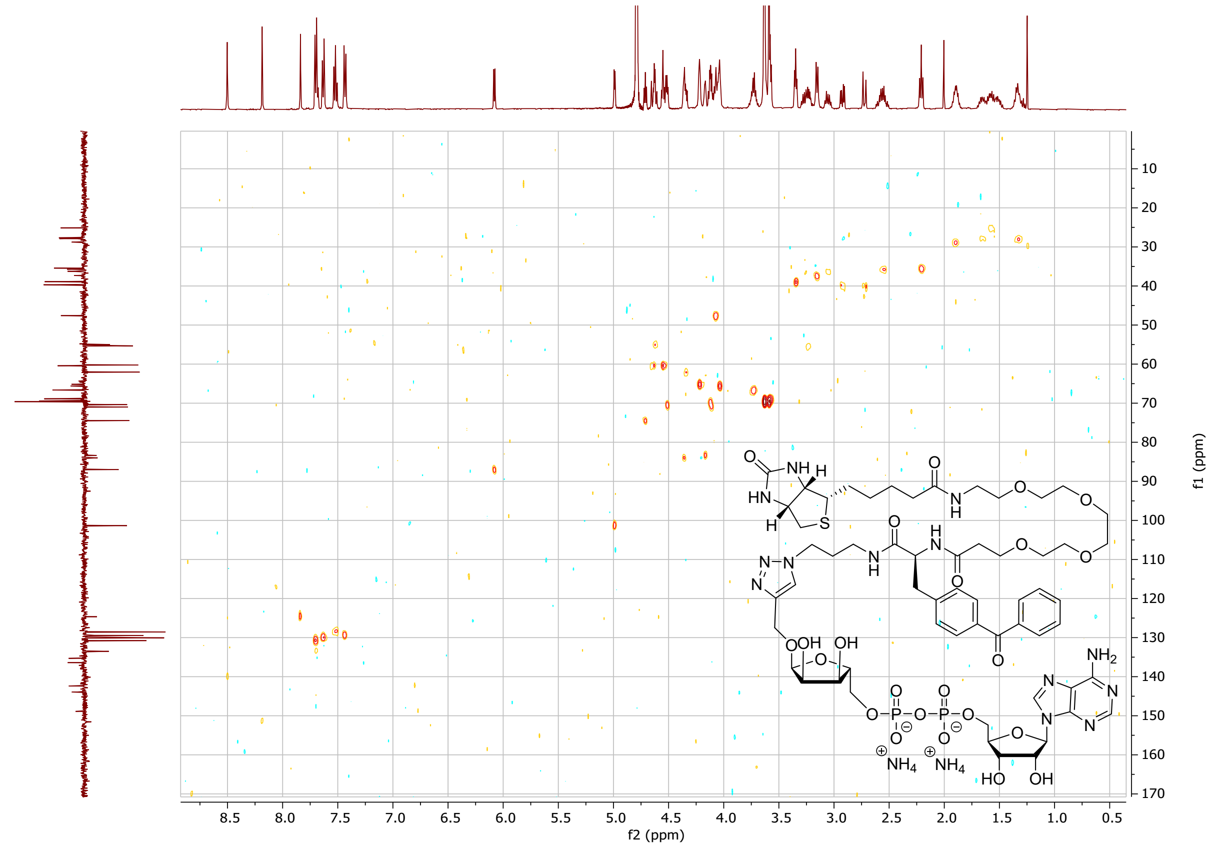

Figure S32: HSQC Spectrum of Compound **1b**

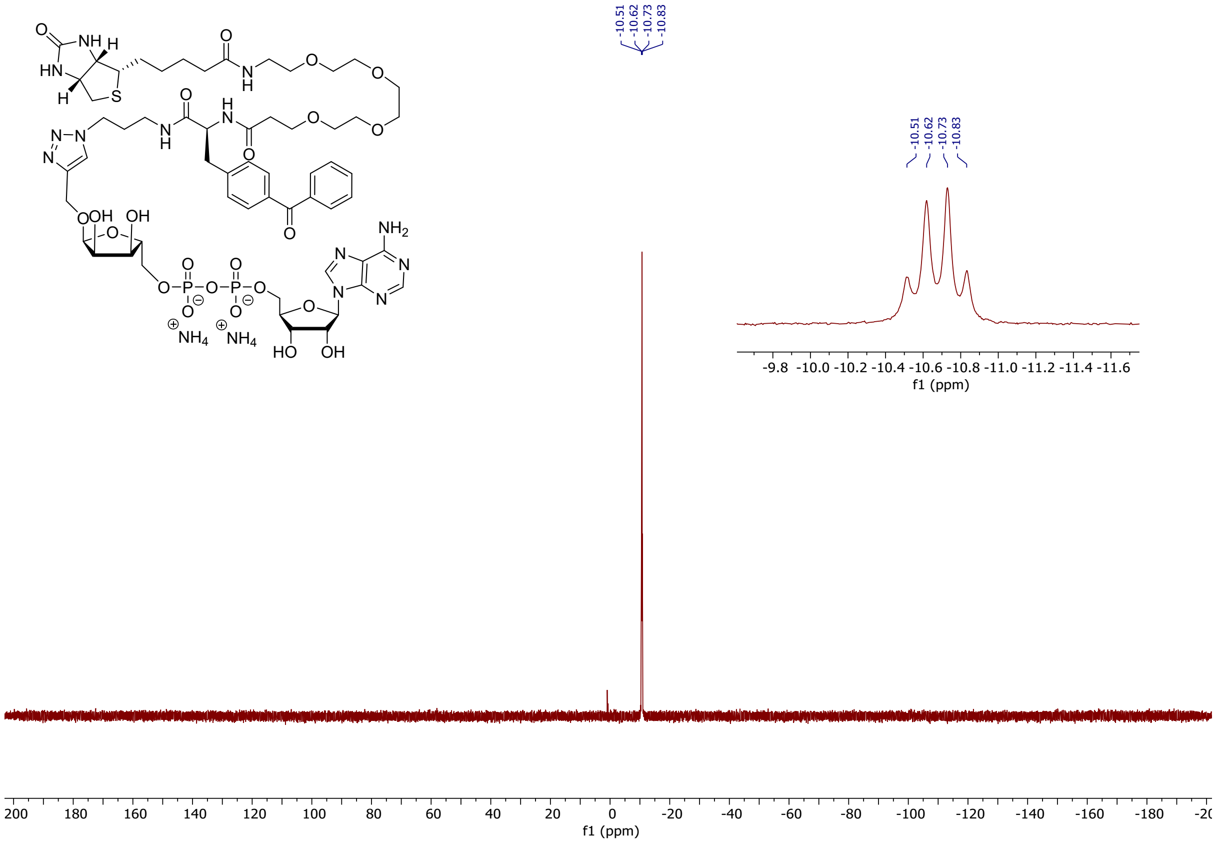

Figure S33: ^{31}P NMR Spectrum of Compound **1b**

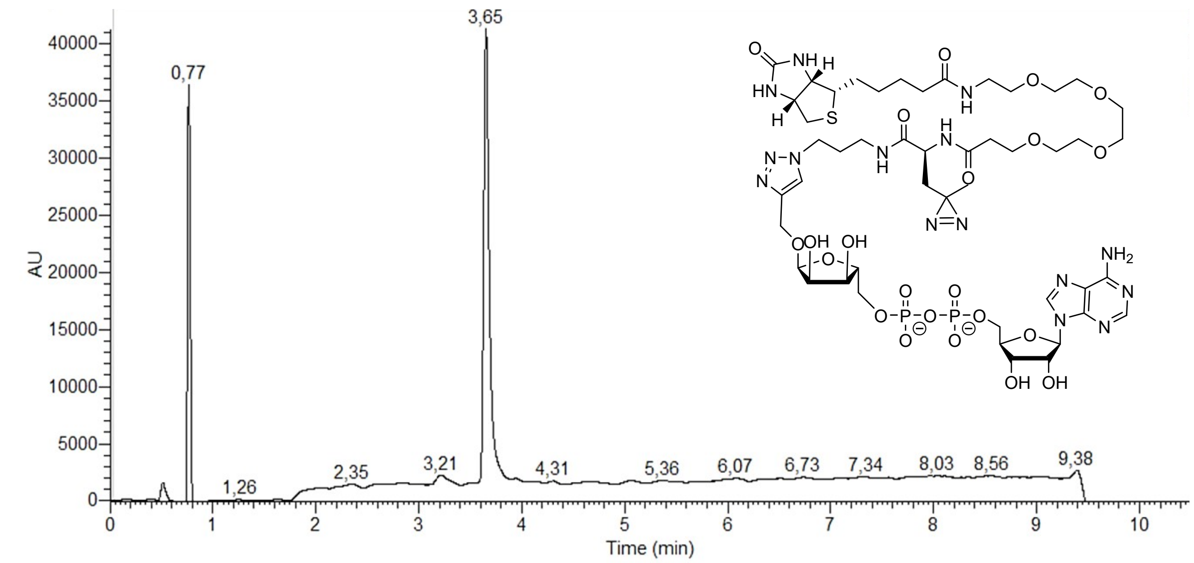

Figure S34: LCMS Analysis of Compound **1a**

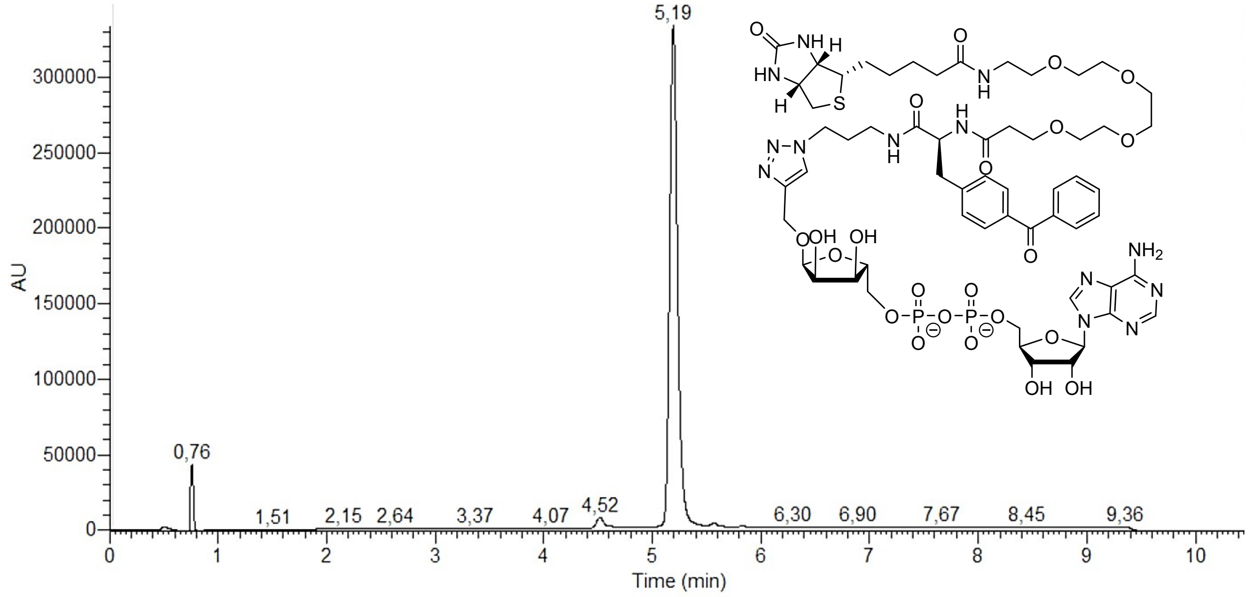

Figure S35: LCMS Analysis of Compound **1b**

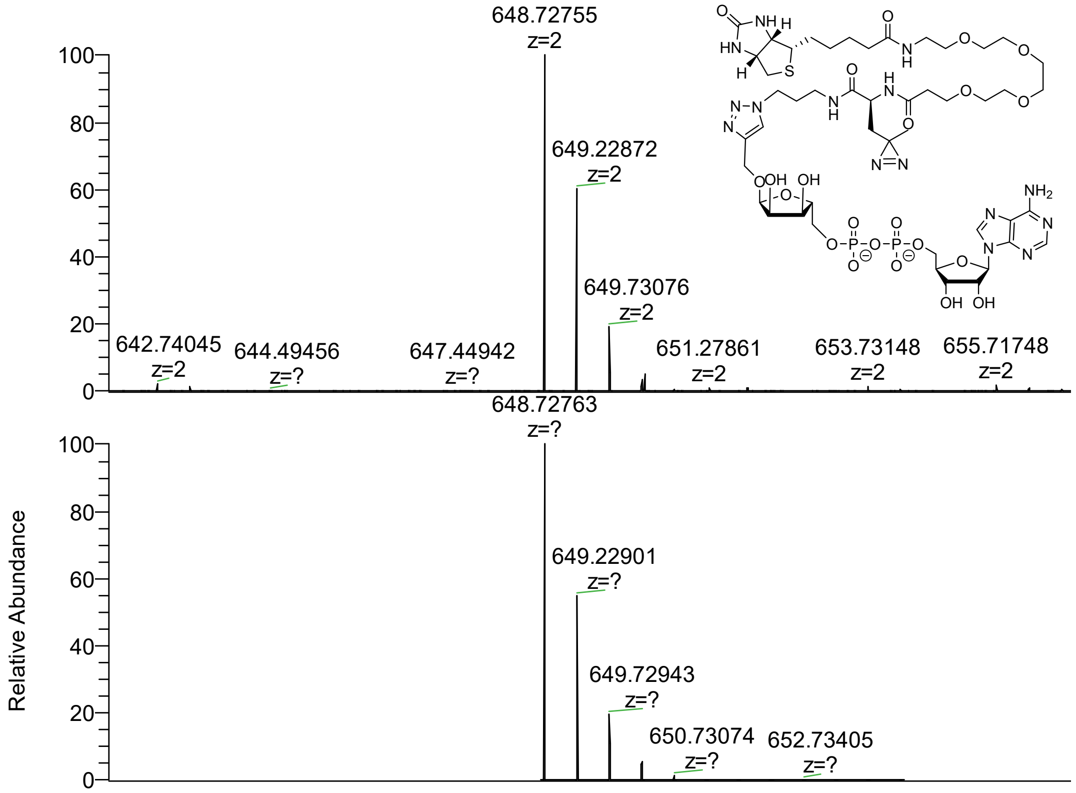

Figure S36: HRMS Data of Compound **1a**

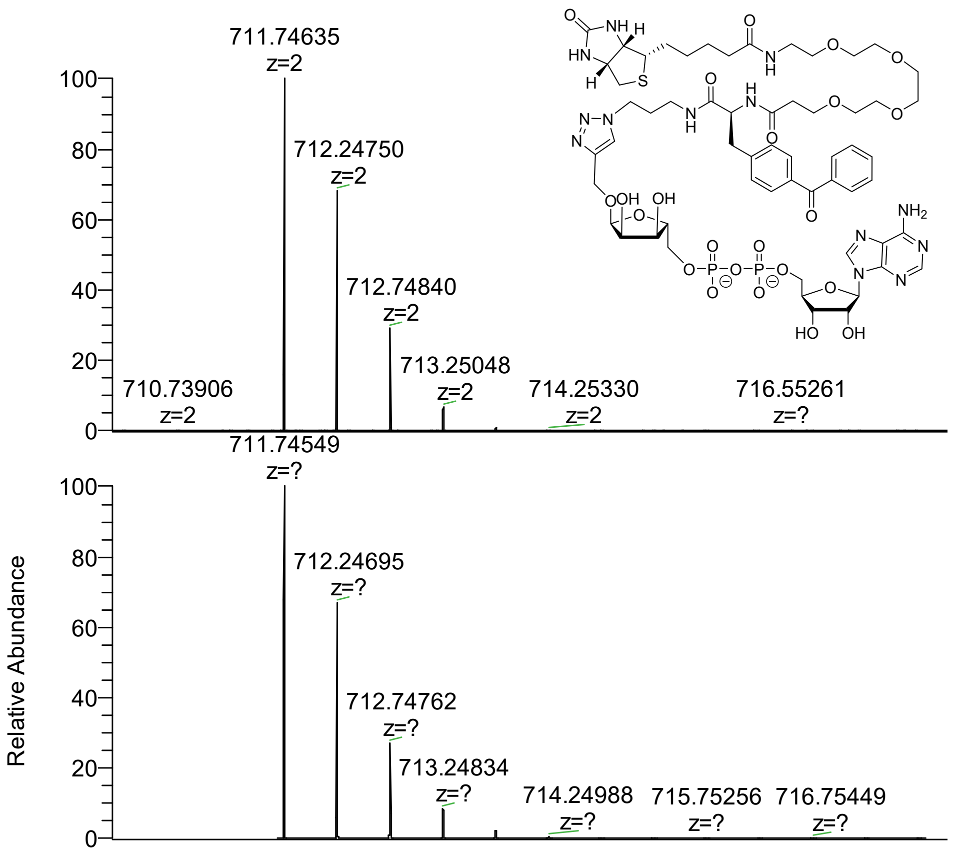

Figure S37: HRMS Data of Compound **1b**

4. Additional References

1 K. W. Kliza, Q. Liu, L. W. M. Roosenboom, P. W. T. C. Jansen, D. V. Filippov and M. Vermeulen, *Mol. Cell.*, 2021, **81**, 4552-4567.e8.

2 M. Dasovich, M. Q. Beckett, S. Bailey, S. E. Ong, M. M. Greenberg and A. K. L. Leung, *J. Am. Chem. Soc.*, 2021, **143**, 3037–3042.

3 A. T. Lam, X. N. Zhang, V. V. Courouble, T. S. Strutzenberg, H. Pei, B. L. Stiles, S. G. Louie, P. R. Griffin and Y. Zhang, *ACS Chem. Biol.*, 2021, **16**, 389–396.

4 B. Gu Kang, S.-U. Kang, J. Jin Kim, J.-S. Kwon, J.-P. Gagné, S. Yun Lee, S. Kim, K. S. Lee, S. Ha, J. Seop Jeong, Y.-I. Lee, H. Zhu, D. Kim, G. G. Poirier, H. Chul Kang, V. L. Dawson and T. M. Dawson, *Nucleic Acids Res.*, 2025, **53**, gkaf300.

5 T. Yang, Z. Liu and X. D. Li, *Chem. Sci.*, 2015, **6**, 1011–1017.

6 Q. Liu, H. A. V. Kistemaker, S. Bhogaraju, I. Dikic, H. S. Overkleeft, G. A. van der Marel, H. Ovaa, G. J. van der Heden van Noort and D. V. Filippov, *Angew. Chem. Int. Ed.*, 2018, **57**, 1659–1662.

7 C. G. Spruijt, F. Gnerlich, A. H. Smits, T. Pfaffeneder, P. W. T. C. Jansen, C. Bauer, M. Münzel, M. Wagner, M. Müller, F. Khan, H. C. Eberl, A. Mensinga, A. B. Brinkman, K. Lephikov, U. Müller, J. Walter, R. Boelens, H. van Ingen, H. Leonhardt, T. Carell and M. Vermeulen, *Cell*, 2013, **152**, 1146–1159.

8 Y. Perez-Riverol, C. Bandla, D. J. Kundu, S. Kamatchinathan, J. Bai, S. Hewapathirana, N. S. John, A. Prakash, M. Walzer, S. Wang and J. A. Vizcaíno, *Nucleic Acids Res.*, 2025, **53**, D543–D553.

9 V. Demichev, C. B. Messner, S. I. Vernardis, K. S. Lilley and M. Ralser, *Nat. Methods*, 2020, **17**, 41–44.

10 S. Tyanova, T. Temu, P. Sinitcyn, A. Carlson, M. Y. Hein, T. Geiger, M. Mann and J. Cox, *Nat. Methods*, 2016, **13**, 731–740.
